# Single-Step Synthesis of 1,2-Cyclopentanediones by Dehydrogenative Annulation of Ethylene Glycol with Secondary Alcohols

**DOI:** 10.1021/jacs.5c14910

**Published:** 2025-11-25

**Authors:** Lijun Lu, Jie Luo, Michael Montag, Yael Diskin-Posner, David Milstein

**Affiliations:** † Department of Molecular Chemistry and Materials Science, 34976 Weizmann Institute of Science, Rehovot 76100, Israel; ‡ Department of Chemical Research Support, Weizmann Institute of Science, Rehovot 76100, Israel

## Abstract

Alcohol coupling reactions that are induced by alcohol dehydrogenation, and generate hydrogen or water as the only byproducts, have become a well-recognized way to carry out important synthetic transformations, such as esterification and alkylation, in a green and atom-economical fashion. Herein, we report a new type of alcohol–alcohol coupling reaction that involves the dehydrogenative annulation of ethylene glycol with secondary alcohols to give 1,2-cyclopentanedione derivatives in a single synthetic step. This process, which is catalyzed by a pincer complex of earth-abundant manganese, represents a new approach for constructing structurally complicated products from inexpensive, readily available alcohols.

## Graphical abstract

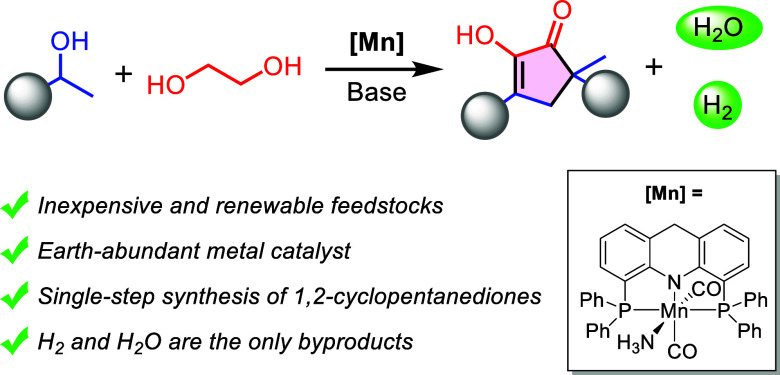

## Introduction

The acceptorless dehydrogenative coupling of alcohols with each other or with other functional groups, catalyzed by metal complexes, has developed over the past two decades to become a powerful synthetic technique for the construction of C–C and C–heteroatom bonds.^1–4^ This kind of coupling reaction requires no preactivated substrates, no sacrificial hydrogen acceptors and no oxidants, and releases H_2_ as the sole byproduct.^5,6^ It can offer a green and economical means of synthesizing valuable chemicals, especially if readily available alcohols from renewable sources are used as the only starting materials. Nevertheless, the selectivity of such alcohol–alcohol cross-coupling reactions is a highly challenging aspect, because of competition from homocoupling and other side reactions.

Dehydrogenative cross-coupling of two different alcohols can result in esterification. In 2012, we reported the selective generation of cross-esters from primary and secondary alcohols, catalyzed by a Ru–pincer complex (Figure 1a).^7^ The observed chemoselectivity of this catalytic system was attributed to the difference in rate between the desired cross-esterification of the primary and secondary alcohols, and the undesired dehydrogenation of the secondary alcohol into ketone, which is a slower process. Moreover, any self-esterification of the primary alcohol in this system can be counteracted in situ through transesterification with the secondary alcohol, thereby affording the requested cross-ester.^8^ More recently, we showed that a Mn–pincer complex can catalyze the dehydrogenative cross-coupling of two different primary alcohols into the corresponding cross-esters.^9^ It should be noted that replacing precious metal catalysts, like those of ruthenium, with catalysts of earth-abundant, low-toxicity base metals, such as manganese, is a highly desirable goal, in and of itself, from a sustainability perspective.^10,11^

**Figure 1:**
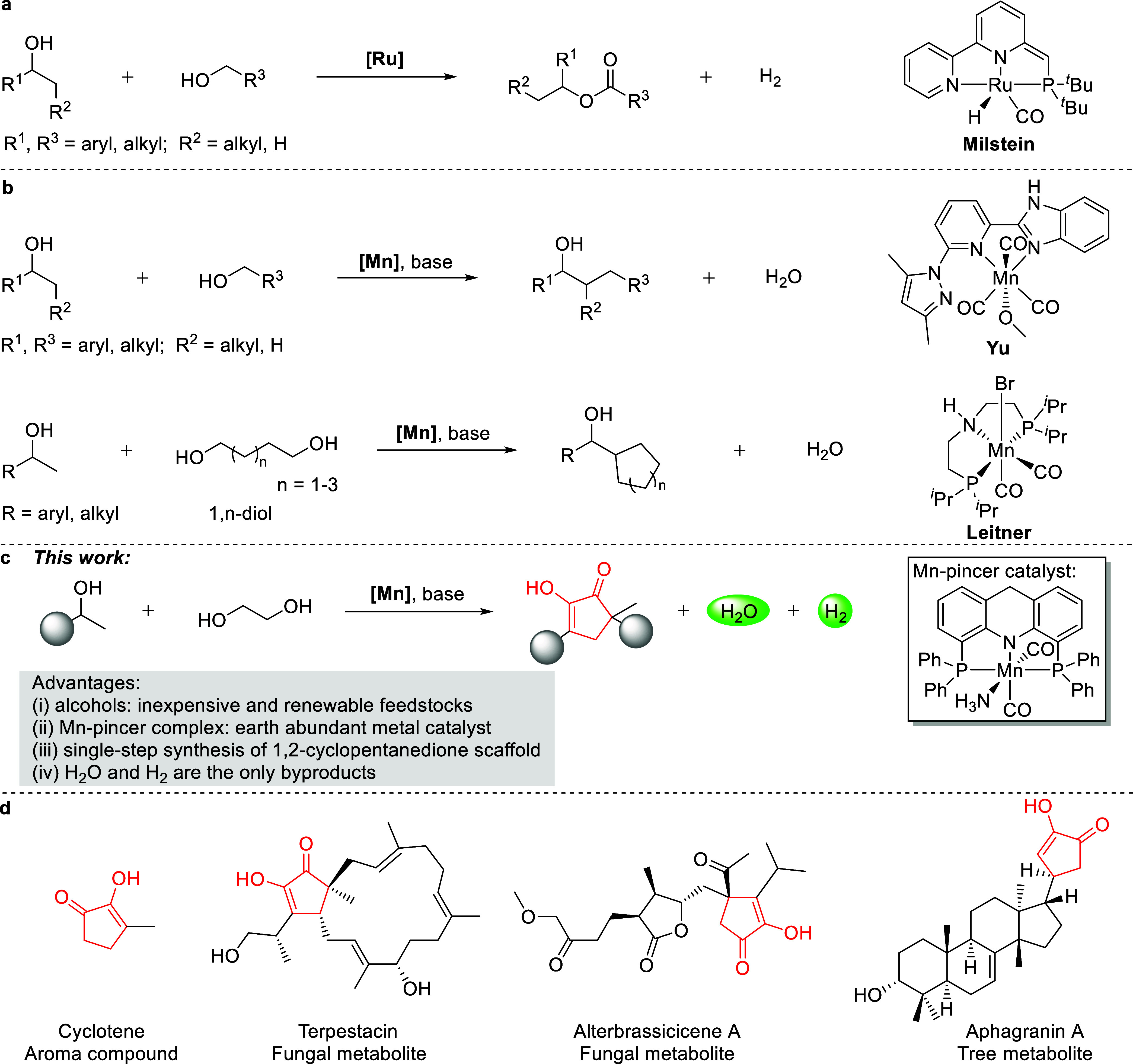
Cross-coupling reactions between primary and secondary alcohols. (a) Dehydrogenative cross-esterification between primary and secondary alcohols. (b) β-Alkylation of secondary alcohols with primary alcohols. (c) Mn-catalyzed annulation of ethylene glycol and secondary alcohols into 1,2-cyclopentanediones (this work). (d) Representative structures with a 1,2-cyclopentanedione core.

Another synthetic approach involving alcohol–alcohol cross-coupling, which is also induced by alcohol dehydrogenation, is the alkylation of one alcohol by another alcohol.^12^ This C–C bond formation reaction is made possible by the so-called hydrogen borrowing methodology, also known as hydrogen autotransfer.^13–16^ This type of redox-neutral protocol is atom-economical and highly environmentally benign, often producing water as the only byproduct. In this context, the use of primary alcohols for the β-alkylation of secondary alcohols has been a topic of interest over the past two decades.^17^ It was first reported in 2005 by Yamaguchi and co-workers, who employed an iridium catalyst in the absence of any hydrogen donor or acceptor.^18^ Subsequently, complexes of more abundant 3d transition metals, such as chromium,^19–21^ iron,^22,23^ cobalt^24–26^ and nickel,^27–30^ were also reported to catalyze such reactions. Manganese complexes that can catalyze the same kind of reaction were first described in 2018 by Yu and co-workers (Figure 1b).^31^ Soon thereafter, the groups of Rueping, Leitner and Kempe separately achieved similar transformations using Mn­(I) complexes of PNN- and PNP-type pincer ligands.^32–34^ In 2019, Leitner and co-workers described the utilization of a Mn­(I)-PNP pincer complex as catalyst for secondary alcohol alkylation, but in this case they used primary diols as alkylating agents, instead of monoalcohols, thereby allowing them to synthesize substituted cycloalkanes via intramolecular double β-alkylation (Figure 1b).^35^ An intermolecular variant of such a double β-alkylation was recently reported by Liu and co-workers, who used Mn­(I)-PNP complexes as catalysts for the multicomponent coupling of secondary and primary alcohols.^36^ It should also be noted that in some catalytic systems, β-alkylation of secondary alcohols with primary ones can lead to monoketone products.^37–39^

In the present report, we introduce a new type of alcohol–alcohol cross-coupling reaction, whereby a secondary alcohol combines with a primary diol to selectively give a cyclic 1,2-diketone instead of undergoing esterification or alkylation. Thus, we show that a Mn­(I)-PNP pincer complex can catalyze the coupling of ethylene glycol (EG) with a variety of secondary alcohols to afford derivatives of 1,2-cyclopentanedione in a single synthetic step that involves an unprecedented dehydrogenative annulation reaction, with H_2_ and water being the only byproducts (Figure 1c). The 1,2-cyclopentanedione scaffold is a core structure in various natural products and aroma compounds (Figure 1d),^40–43^ and can also function as a carboxylic acid bioisostere, in the context of medicinal chemistry.^44^ It should be noted that when such a cyclic diketone possesses an α-hydrogen atom, the 1-keto-2-enol tautomer is often the predominant one, rather than the parent diketone.^45^ The syntheses of such 5-membered cyclic diketones usually require specialized starting materials or multistep procedures,^46–49^ thereby limiting their practical utility. Our new method, which relies on readily available alcohols as starting materials, and can also employ ketones instead of secondary alcohols, does not produce noxious waste and offers an efficient, atom-economical and environmentally benign alternative to existing procedures for the synthesis of such complicated molecular frameworks.

## Results and Discussion

We have previously demonstrated that EG can be used as a readily available feedstock material for hydrogen storage and synthetic applications, made possible through dehydrogenative coupling reactions catalyzed by Ru- and Mn-pincer complexes.^50–54^ As part of our ongoing efforts to utilize EG in this context, we examined its coupling with secondary alcohols, beginning with 1-phenylethanol (PE). Previous reports indicate, either explicitly or implicitly, that these two alcohols can be directly coupled with each other through three types of reactions, namely, esterification,^55^ single β-alkylation,^35^ or double β-alkylation^31^ (Figure 2a).

**Figure 2:**
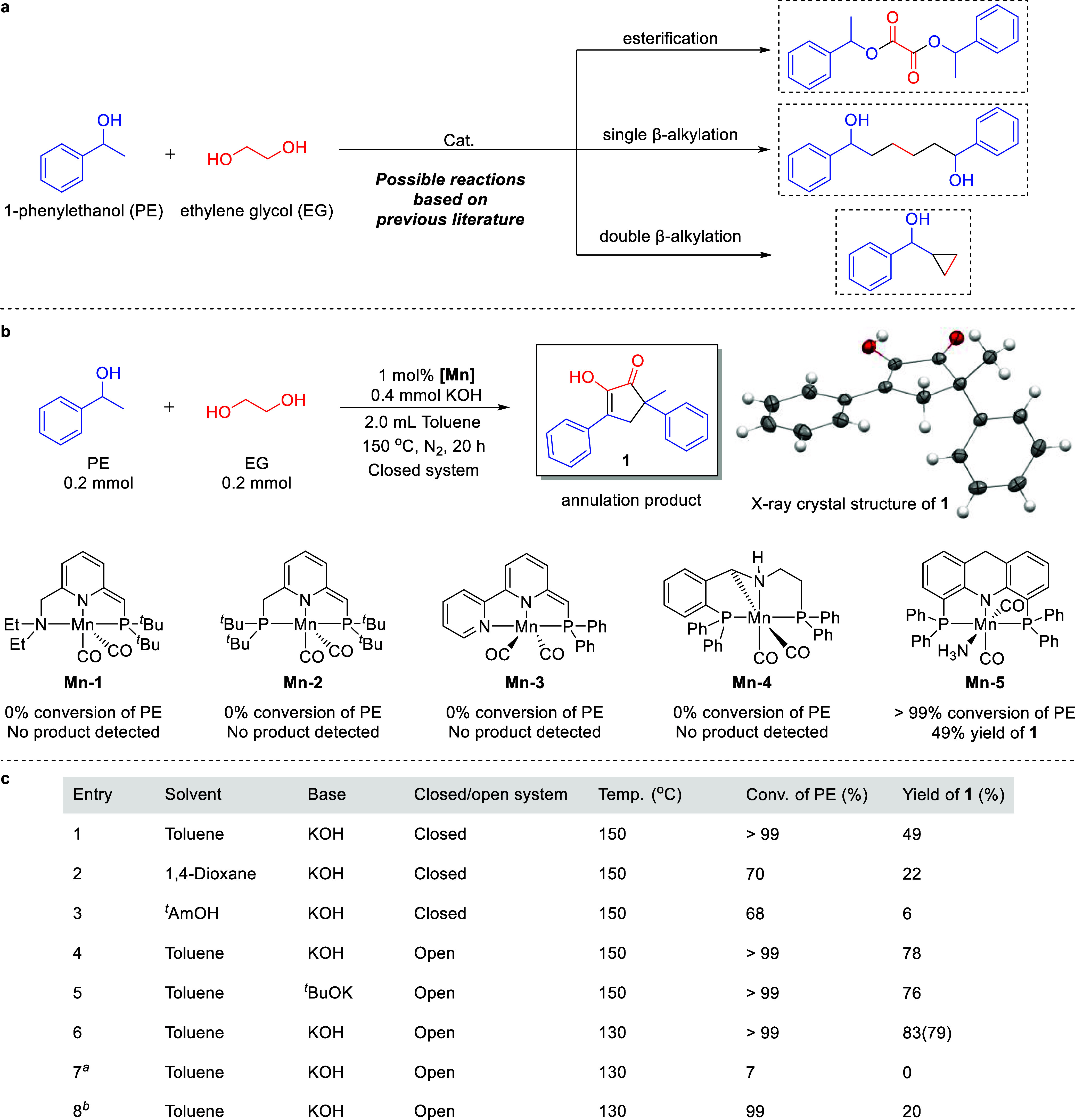
Catalytic cross-coupling between ethylene glycol and 1-phenylethanol. (a) Possible direct coupling reactions between EG and PE, based on previous reports. (b) Complexes investigated as potential catalysts and the molecular structure of product **1** (thermal ellipsoids in the crystal structure are set at the 50% probability level); the reaction involving each of the complexes was conducted by combining EG (0.2 mmol), PE (0.2 mmol), Mn complex (1.0 mol % vs PE), KOH (0.4 mmol) and toluene (2.0 mL), and heating the mixture in a sealed glass tube at 150 °C for 20 h. (c) Optimization of catalytic conditions, including solvent, base, closed/open system and temperature; each of the reactions was conducted by combining EG (0.2 mmol), PE (0.2 mmol), **Mn**–**5** (1.0 mol % vs PE), KOH (0.4 mmol) and toluene (2.0 mL), and heating the mixture at the specified temperature for 20 h, either in a sealed glass tube (closed system), or in a Schlenk tube under N_2_ flow (open system); yields were determined by ^1^H NMR spectroscopy, using dibromomethane as internal standard (isolated yield is noted in parentheses). ^*a*^0.2 mmol of KOH instead of 0.4 mmol. ^*b*^0.1 mmol of EG instead of 0.2 mmol.

In an attempt to couple EG and PE, we first screened a number of Mn-pincer complexes previously developed by our group, i.e., **Mn**–**1**,^56^ **Mn**–**2**,^57^ **Mn**–**3**,^9^ **Mn**–**4**,^58^ and **Mn**–**5** ^53^ (Figure 2b). These complexes have been shown to catalyze alcohol coupling through redox-neutral metal–ligand cooperation involving either dearomatization/aromatization cycling (**Mn**–**1**, **Mn**–**2** and **Mn**–**3**) or amido/amino interconversion (**Mn**–**4** and **Mn**–**5**). Each of these potential catalysts was applied in toluene at a loading of 1.0 mol % (vs PE), together with 0.2 mmol of EG, 0.2 mmol of PE and 0.4 mmol of KOH, and the resulting slurry was stirred for 20 h at a nominal temperature of 150 °C, in a sealed glass tube. Under these conditions, the first four complexes were found to be inactive toward EG-PE coupling. However, when **Mn**–**5** was employed as catalyst, a coupling product was observed, but it was different from the esterification or β-alkylation products shown in Figure 2a. Unexpectedly, it was found to be the annulation product **1** (Figure 2b), which was obtained in 49% yield (Figure 2c; table entry 1). The structure of this new organic compound, which is the enol form of the corresponding 1,2-cyclopentanedione, was confirmed by nuclear magnetic resonance (NMR) spectroscopy and X-ray crystallography (Figure 2b; see Supporting Information). Using the same catalyst and substrates, we then set to optimize the reaction conditions, in an effort to improve the yield of **1**. When toluene was replaced with other solvents, i.e., dioxane or ^*t*^AmOH (^*t*^Am = *tert*-amyl), the yield dropped dramatically, to 22 and 6%, respectively (Figure 2c; entries 2 and 3). By contrast, repeating the reaction in toluene, albeit in an open system under N_2_ flow, so that H_2_ gas can escape the system as it is generated, enabled us to increase the yield of **1** to 78% (entry 4). Replacing KOH with ^*t*^BuOK in the same open catalytic system gave **1** in similar yield (76%; entry 5). Interestingly, lowering the nominal reaction temperature to 130 °C resulted in a higher yield of 83% (79% isolated yield, entry 6). By contrast, decreasing the amount of KOH to 0.2 mmol (1.0 equiv per PE) was found to be highly detrimental to this transformation, with none of the desired product being detected after heating at 130 °C (entry 7). Finally, when the amount of EG was reduced to 0.1 mmol (0.5 equiv per PE), the yield of **1** fell to only 20% (entry 8).

With the optimized catalytic conditions in hand (Figure 2c; table entry 6), we explored the substrate scope of our Mn-catalyzed annulation of EG with secondary alcohols (Figure 3). To this end, a series of secondary aryl alcohols was investigated, each of which was mixed with 1.0 equiv of EG, 2.0 equiv of KOH, **Mn**–**5** at 1.0 mol % loading (vs secondary alcohol), and toluene as solvent, and the resulting mixture was stirred at 130 °C for 20 h, in an open system under N_2_. As noted above, under these conditions PE reacted with EG to give product **1** in 79% isolated yield. When the amount of these substrates was scaled-up 30-fold, over 0.5 g of this product was obtained, representing 65% yield. As shown in Figure 3, other secondary alcohols afforded the corresponding 1,2-cyclopentanediones in fair-to-good yields. Thus, PE with a methyl substituent at either the *para* or *meta* position, as well as a variant bearing a *tert*-butyl group at the *para* position, were transformed into the respective products, **2**, **3** and **4**, in 70–74% yield. A phenyl-substituted variant, 1-(4-phenylphenyl)­ethanol, gave the corresponding product, **5**, in 60% yield. PE exhibiting the electron-donating groups *para*-alkoxy, -phenoxy, -methylthio and -dimethylamino, or the electron-withdrawing substituents *meta*-F, *para*-Cl and *meta*-CF_3_, afforded the desired products, **6**–**13**, in 55–78% yield. Two polycyclic aromatic alcohols, 1-(2-naphthyl)­ethanol and 1-(3-phenanthryl)­ethanol, were also converted into the corresponding annulation products, **14** and **15**, which were obtained in 65 and 61% yield, respectively. Importantly, replacing each of the above secondary alcohols with its respective ketone afforded the same products, but in consistently higher yields, ranging from 65 to 85% (**1**–**15**). Lastly, as proof of concept for the potential application of our methodology to the synthesis of complex natural products, we utilized EG and the commercially available ketone 3-acetyl-1-methylindole for the single-step synthesis of compound **16** (Scheme 1), which is reminiscent of the fungal metabolite Asterredione.^59,60^ Taken together, the above examples demonstrate that our new synthetic strategy can provide a simple, economical way to construct 1,2-cyclopentanedione derivatives, which have thus far been synthesized using complicated substrates and reaction conditions.^61,62^ It should be noted that aliphatic secondary alcohols and ketones were found to be incompatible with the present catalytic system (see Supporting Information).

**Figure 3:**
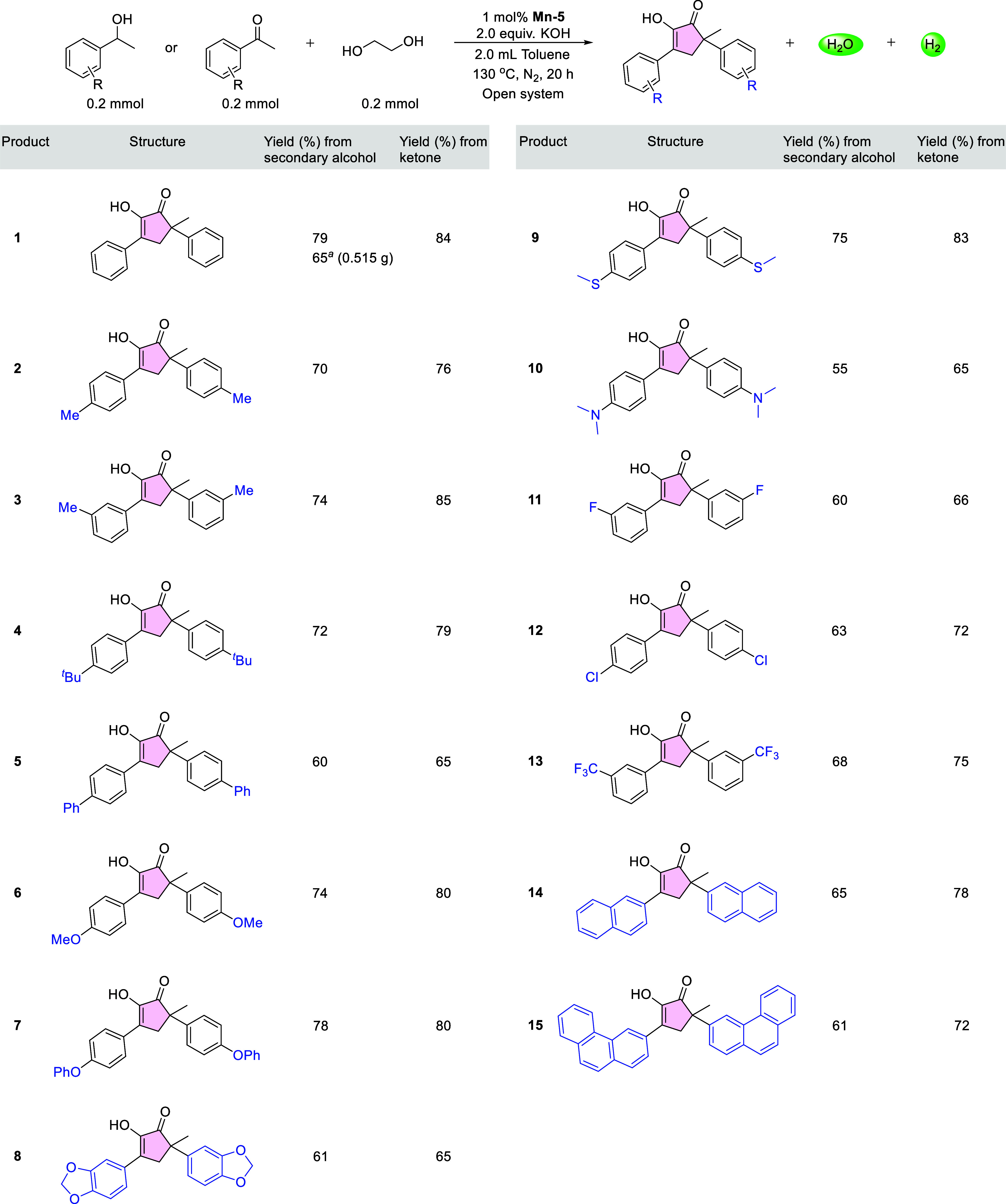
Substrate scope of ethylene glycol annulation with secondary alcohols or ketones catalyzed by **Mn**–**5**. Each of the reactions presented in this figure was conducted using a secondary alcohol or ketone (0.2 mmol), EG (0.2 mmol), **Mn**–**5** (1.0 mol % vs secondary alcohol or ketone), KOH (0.4 mmol) and toluene (2.0 mL), and heating the resulting mixture at 130 °C for 20 h in a Schlenk tube connected to an N_2_ line. The displayed yields are those of isolated products. ^*a*^ The amount of substrates was scaled up 30-fold to 6.0 mmol of PE and 6.0 mmol of EG, giving 0.515 g of product **1**.

**Scheme 1:**
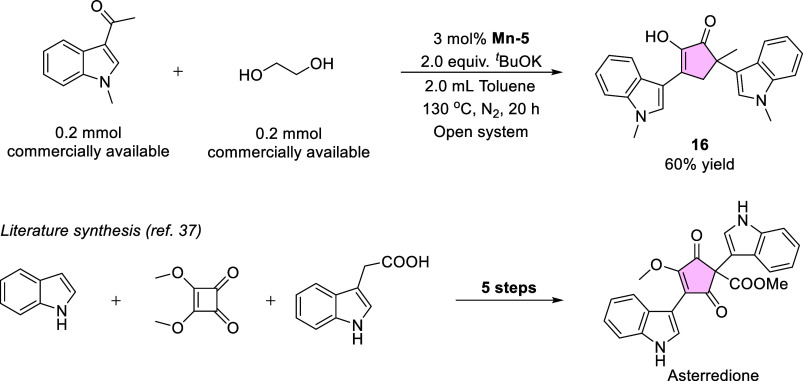
Single-step Synthesis of a Compound Similar to the Fungal Metabolite Asterredione

In an attempt to elucidate the mechanism of these ring-forming transformations, several control experiments were conducted (Scheme 2). The coupling of EG and PE was repeated under the optimized catalytic conditions, but in a closed system, thereby allowing us to directly observe the generation of H_2_ by gas chromatography and H_2_O by ^1^H NMR spectroscopy (see Supporting Information). We also reran this experiment in an open system, and analyzed the composition of organic products. The desired annulation product **1** was obtained in 80% yield, together with acetophenone and 1,3-diphenyl-1-butanone (**17**) in 2 and 7% yield, respectively (Scheme 2a). As can be seen in Figure 3, acetophenone was also found to be a suitable substrate for our Mn-catalyzed annulation with EG, affording compound **1** in 84% yield. Based on these results, we surmised that in the initial stage of catalysis, **Mn**–**5** dehydrogenates PE into acetophenone. Once generated, this ketone is expected to undergo base-catalyzed self-ketol condensation–a well-established transformation that affords the highly conjugated chalconoid product 1,3-diphenyl-2-buten-1-one (**18**; Scheme 2b).^63^ Although the latter was not directly observed in the reaction mixture containing product **1**, its presence was supported by control experiments. Thus, when we repeated the catalytic reaction under the optimized conditions, but with only 0.5 equiv of EG relative to PE, compound **1** was obtained in only 20% yield, and was accompanied by chalconoid **18** in 7% yield, its hydrogenated derivative **17** in 29% yield, and acetophenone in 30% yield. In a separate experiment, EG was directly combined with 0.5 equiv of **18**, and the mixture was subjected to the same catalytic conditions, affording **1** in 55% yield. These results strongly indicate that **18** is indeed an intermediate in the annulation of EG with PE.

**Scheme 2:**
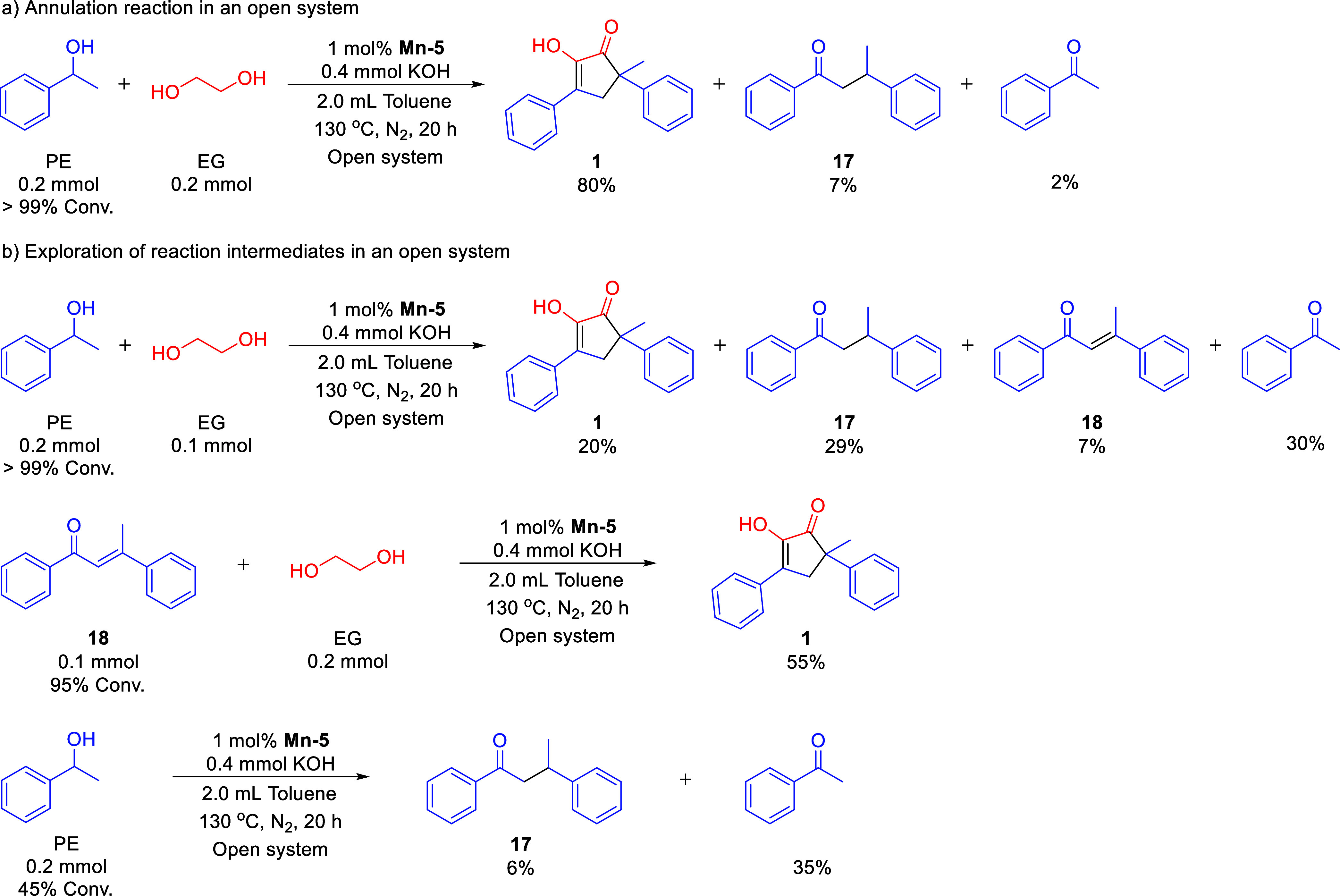
Mechanistic Control Experiments for the Annulation of Ethylene Glycol with 1-Phenylethanol Catalyzed by Mn-5 under Alkaline Conditions

The fact that compound **17** was observed alongside **1** implies that **18** is hydrogenated into **17** through a side-reaction mediated by **Mn**–**5**, involving hydrogen extracted from the alcohol. This was further corroborated when the optimized catalytic conditions were applied to PE alone, leading to the formation of acetophenone in 35% yield, alongside **17** in 6% yield, with no observable quantities of intermediate **18** or product **1**. Interestingly, in this case, as well as in the above-mentioned experiment involving 0.5 equiv of EG vs PE, a significant amount of acetophenone was generated, but did not self-condense into the chalconoid. We attribute this to lack of sufficient EG. This diol is expected to enhance the solubility of KOH in the reaction medium through increased polarity, and possibly chelation of the potassium cations, and its absence lowers the solubility of the base, thereby hampering the self-condensation of acetophenone.

Lastly, we performed a catalytic run involving EG and PE, with time-resolved in situ sampling to analyze the composition of the reaction mixture over time (see Supporting Information). In this experiment, we observed the gradual consumption of PE and concurrent formation of product **1**, with little accumulation of acetophenone and side-product **17**, and no significant buildup of intermediate **18**. In the early stages of the reaction, the conversion of PE into acetophenone is very slow, requiring ∼2 h for the ketone to become detectable, and this suggests that PE dehydrogenation is the rate-determining step of the entire annulation process. This conclusion is supported by our computational results (see below).

Based on the above results, as well as our previous work,^53^ we propose a plausible mechanism for the Mn-catalyzed annulation of EG with secondary alcohols under alkaline conditions, as outlined in Scheme 3. As mentioned above, PE is initially dehydrogenated by **Mn**–**5** to generate acetophenone (**I**), which then converts into intermediate **II** (compound **18**) through base-catalyzed self-condensation. In a similar manner, EG is dehydrogenated into glycolaldehyde (**III**),^64^ which is then deprotonated and proceeds to couple with **II** through Michael addition,^65,66^ thereby affording intermediate **IV**. Subsequently, this intermediate is isomerized into species **VII** via two alternative pathways. One pathway (*a*) involves the dehydrogenation of **IV** into intermediate **V**, which is catalyzed by **Mn**–**5** and is followed by hydrogenation of **V** into **VII**, promoted by the hydrogenated variant of the catalyst, **[Mn**–**5]­H**_**2**_. The second pathway (*b*) involves the hydrogenation of **IV** into intermediate **VI**, mediated by **[Mn**–**5]­H**_**2**_, after which **VI** is dehydrogenated by **Mn**–**5** to give **VII**. Subsequently, **VII** undergoes cyclization into the desired product **1** through base-induced ketol condensation involving intermediate **VIII**.

**Scheme 3:**
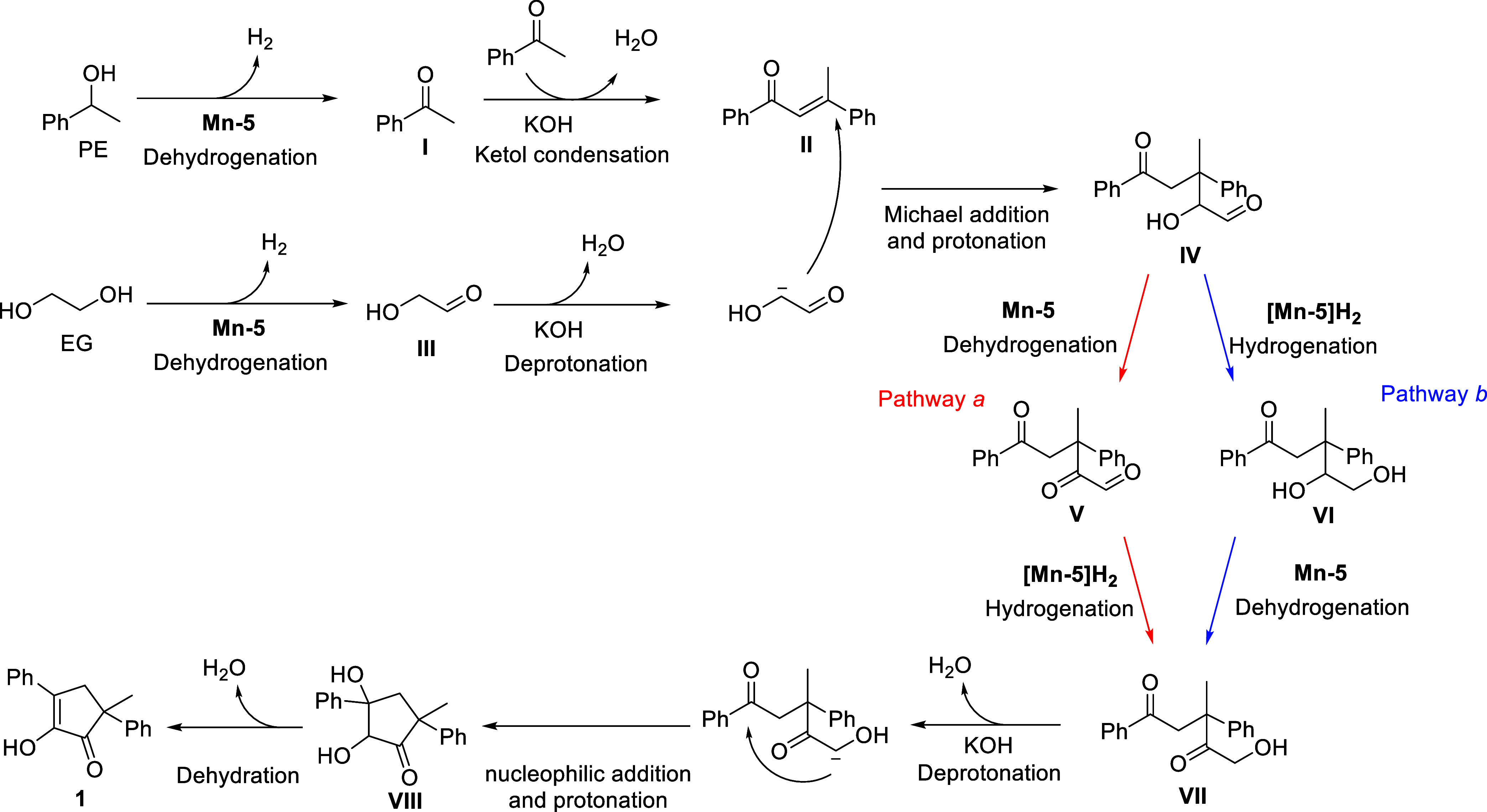
Proposed Mechanism for the Annulation of Ethylene Glycol with 1-Phenylethanol Catalyzed by Mn-5 under Alkaline Conditions

The proposed mechanism was further explored computationally, using density functional theory (DFT) calculations. This allowed us to construct reaction profiles for the various processes, as depicted in Figure 4. Since toluene is our experimental solvent of choice, we first calculated the profiles using this nonpolar medium as an implicit solvent. However, the reaction mixture also contains alcohols, which increase its effective polarity. Therefore, for comparative purposes, we also calculated all profiles using the highly polar EG instead of toluene. The polarity of the liquid reaction mixture, which varies as the catalytic reaction progresses, is expected to be between those two extremes.

**Figure 4:**
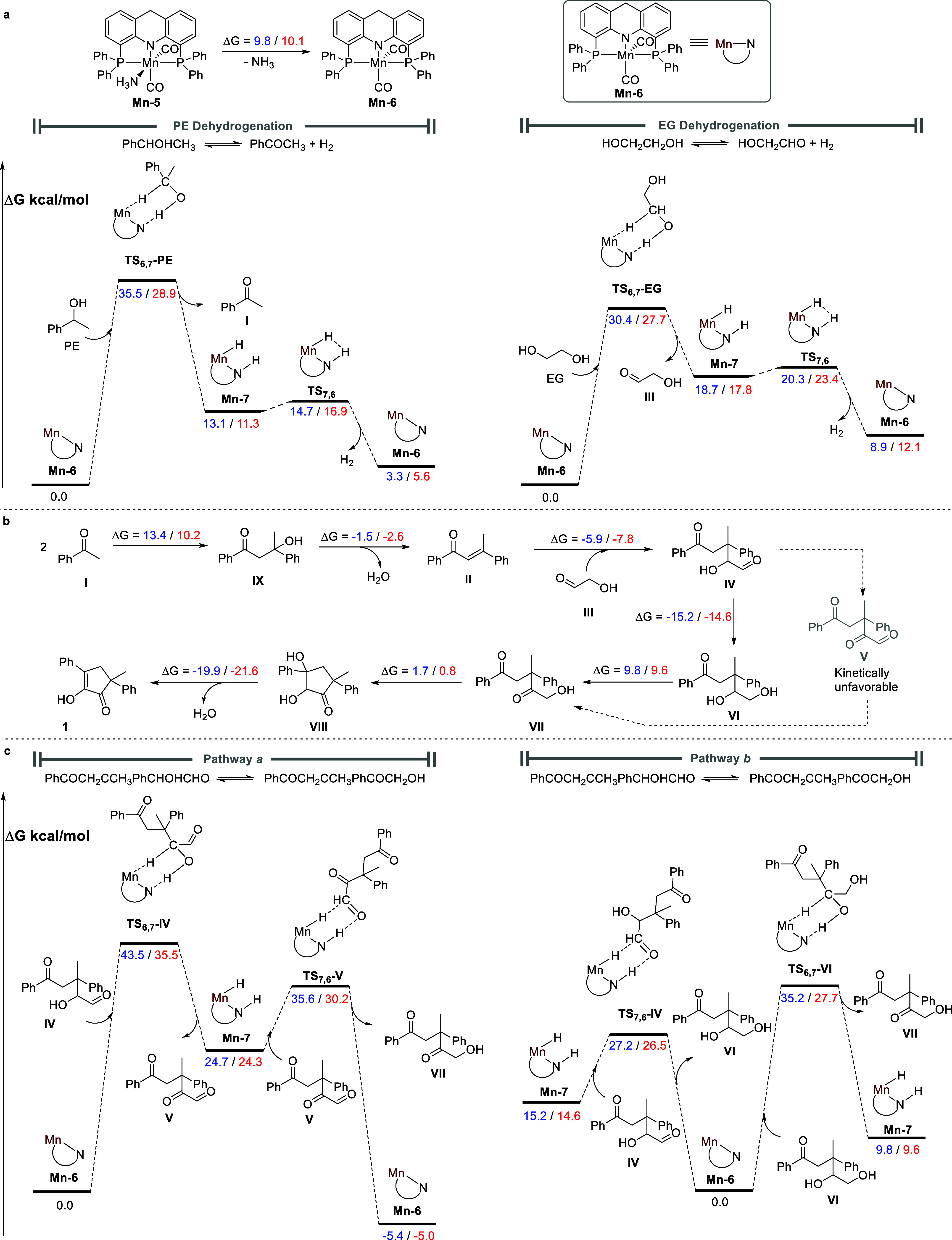
Computed reaction profiles. (a) Mn-catalyzed dehydrogenation of alcohols. (b) Base-promoted annulation process. (c) Mn-catalyzed isomerization of intermediate **IV** into **VII**. All of the noted values are free energies in kcal/mol; in each pair of energy values, the one on the left (blue) was calculated using toluene as an implicit solvent and the one on the right (red) was calculated using EG as an implicit solvent.

As has been previously demonstrated, the catalytically active species is not **Mn**–**5**, but rather the five-coordinate complex **Mn**–**6** (Figure 4a), which forms in situ by dissociation of the ammonia ligand, and is ∼10 kcal/mol less stable than **Mn**–**5**, but still energetically accessible.^53^ The coordinatively unsaturated species **Mn**–**6** interacts with PE across the Mn–N bond, thereby dehydrogenating the alcohol into acetophenone, with concomitant formation of the Mn–H complex **Mn**–**7**, which is the aforementioned species **[Mn**–**5]­H**_**2**_. An outer-sphere transition state, **TS**_**6**,**7**_-**PE**, is associated with this step, exhibiting an activation barrier [ΔΔ*G* ^⧧^(**TS**_**6**,**7**_-**PE**)] of 35.5 kcal/mol in toluene, and 28.9 kcal/mol in EG as solvent. This appears to be the rate-determining step of the entire annulation process, in line with our experimental findings (see above). The dehydrogenation of EG into glycolaldehyde proceeds along a closely analogous pathway, albeit with a lower kinetic barrier of ΔΔ*G* ^⧧^(**TS**_**6**,**7**_-**EG**) = 30.4 kcal/mol in toluene, and 27.7 kcal/mol in EG. In both cases, the H_2_-adduct **Mn**–**7**, formed upon alcohol dehydrogenation, can release H_2_ to regenerate **Mn**–**6**, in a process that is highly facile, with ΔΔ*G* ^⧧^(**TS**_**7**,**6**_) = 1.6 kcal/mol in toluene, and 5.6 kcal/mol in EG. Furthermore, the dehydrogenation of both EG and PE is endergonic, to the tune of 8.9 and 3.3 kcal/mol, respectively, in toluene, and 12.1 and 5.6 kcal/mol in EG as solvent. It appears that both reactions are driven by the high substrate concentrations, as well as the release of H_2_ from the reaction mixture, due to the low solubility of this gas.

According to our proposed mechanism, the ring-forming reaction between acetophenone and glycolaldehyde proceeds through a series of steps, most of which do not involve the Mn catalyst. The calculated free energies of these steps are presented in Figure 4b. As noted above, two acetophenone molecules combine into the corresponding β-hydroxy ketone **IX**, which subsequently dehydrates to afford intermediate **II** (compound **18**), in a process that is overall thermodynamically uphill by 11.9 kcal/mol in toluene, and 7.6 kcal/mol in EG. This coupling product is then nucleophilically attacked by glycolaldehyde to generate intermediate **IV**, in a reaction that is energetically downhill by 5.9 kcal/mol in toluene, and 7.8 kcal/mol in EG. Although we cannot rule out the existence of parallel reactions involving glycolaldehyde, such as its self-aldol condensation, or cross-aldol condensation with acetophenone, we have no experimental indications for these reactions. Moreover, as mentioned above, the formation of acetophenone through PE dehydrogenation is ∼6 kcal/mol more favorable than EG dehydrogenation into glycolaldehyde, and therefore the concentration of glycolaldehyde in the reaction mixture is expected to be orders of magnitude lower than that of acetophenone, especially at early stages of the catalytic process. Hence, the self-condensation of acetophenone into the chalconoid intermediate is likely to precede any reaction involving glycolaldehyde.

The isomerization of intermediate **IV** into **VII** may proceed through two alternative routes involving dehydrogenation and hydrogenation mediated by **Mn**–**6** and **Mn**–**7**, respectively, and their corresponding reaction profiles are depicted in Figure 4c. Both pathways are exergonic by ∼5 kcal/mol, but they differ markedly in their kinetic attributes. Pathway *a* begins with the outer-sphere dehydrogenation of **IV** by **Mn**–**6** to give intermediate **V** and **Mn**–**7**, followed by outer-sphere hydrogenation of **V** by **Mn**–**7** to afford **VII** and regenerate **Mn**–**6**. This pathway exhibits a prohibitively high overall kinetic barrier [ΔΔ*G* ^⧧^(**TS**_**6**,**7**_-**IV**)] of 43.5 kcal/mol in toluene, or 35.5 kcal/mol in EG, which is practically insurmountable at 130 °C. Pathway *b* proceeds via analogous outer-sphere mechanisms, but in reverse order, i.e., **Mn**–**7** hydrogenates **IV** to afford intermediate **VI** and **Mn**–**6**, which then react with each other to give **VII** and **Mn**–**7**. The apparent activation energy of this pathway [ΔΔ*G* ^⧧^(**TS**_**6**,**7**_-**VI**)] is 35.2 kcal/mol in toluene, which is still high, but much lower than that of pathway *a*. In EG, however, this kinetic barrier is only 27.7 kcal/mol, which can be easily overcome under the experimental reaction conditions. Hence, pathway *b* is the kinetically preferred route, based on our DFT calculations. Finally, the annulation sequence concludes as **VII** undergoes ring closure to afford intermediate **VIII** in a slightly endergonic process (Δ*G* < 2 kcal/mol), followed by dehydration into product **1**. The latter step exhibits a very large drop in free energy, amounting to 19.9 kcal/mol in toluene, and 21.6 kcal/mol in EG, which constitutes the primary driving force for the entire annulation reaction.

Finally, in an attempt to rationalize the fact that **Mn**–**5** is catalytically active vis-à-vis the annulation reaction, whereas the other four Mn­(I) complexes are not, we undertook a comparative study involving **Mn**–**5** and **Mn**–**2** (see Supporting Information for further details). Both complexes feature PNP-type pincer ligands, but they exhibit different modes of metal–ligand cooperation, namely, amido/amino interconversion for **Mn**–**5** (as in **Mn**–**4**) and dearomatization/aromatization for **Mn**–**2** (as in **Mn**–**1** and **Mn**–**3**). In order to facilitate the comparison, we replaced the P^*t*^Bu_2_ groups of **Mn**–**2** with PPh_2_ groups, and examined the catalytic activity of the resulting complex, **Mn**-**2**-**Ph**. Unlike **Mn**–**2**, its Ph-substituted variant was found to be active, albeit less than **Mn**–**5**, affording product **1** in 22% yield. We also replaced the PPh_2_ groups of **Mn**–**5** with P^*i*^Pr_2_ groups, but the obtained complex, **Mn**-**5**-^*i*^ **Pr**, was found to be catalytically inactive under the optimized reaction conditions. A computational examination of **Mn**-**5**-^*i*^ **Pr** shows that PE dehydrogenation by this ^*i*^Pr-substituted complex, which is a prerequisite for the annulation reaction, is kinetically less feasible than it is for the Ph-substituted **Mn**–**5**, with the former exhibiting a decidedly higher activation barrier, i.e., 39.5 vs 35.5 kcal/mol in toluene. Furthermore, close examination of the transition states for this dehydrogenation step reveals that the bulky ^*i*^Pr substituents of **Mn**-**5**-^*i*^ **Pr** block the approach of the PE substrate to the catalytically active Mn–N fragment of the complex, whereas the Ph substituents of **Mn**–**5** enable better substrate access. It is possible that the relative electron deficiency of the PPh_2_ groups, as compared to the dialkylphosphine ones, also plays a role in enhancing the catalytic activity. Thus, our experimental and computational results demonstrate that the substituents on the pincer phosphine groups, which affect both the steric and electronic properties of the complexes, as well as the exact mode of metal–ligand cooperation, are key factors that influence the ability of the catalyst to promote the dehydrogenative annulation of EG with secondary alcohols.

## Conclusions

In conclusion, we have introduced a new methodology for the synthesis of 1,2-cyclopentanediones through the single-step annulation of ethylene glycol with secondary alcohols or ketones, catalyzed by a Mn­(I)-pincer complex, with H_2_ and water as the only byproducts. This alcohol–alcohol cross-coupling pathway differs from the previously reported procedures, which typically result in alcohol esterification or alkylation, and offers a green alternative for the synthesis of 5-membered cyclic 1,2-diketones from simple, readily available starting materials.

## Supplementary Material



## Notes

Catalyst synthesis, experimental procedures, control experiments, NMR spectra, and computational details (PDF)

## §

Department of Chemistry, Zhejiang University, Hangzhou 310058, P. R. China
