# Supplementary material for: Single-Step Synthesis of 1,2-Cyclopentanediones by Dehydrogenative Annulation of Ethylene Glycol with Secondary Alcohols
Source: J Am Chem Soc. 2025 Nov 25;147(49):45283–93. doi: 10.1021/jacs.5c14910 (PMC12703682; doi:10.1021/jacs.5c14910)
Supplement: Supplementary file 1 [file ja5c14910_si_001.pdf]

Supporting Information for

Single-Step Synthesis of 1,2-Cyclopentanediones by Dehydrogenative Annulation of Ethylene Glycol with Secondary Alcohols

Lijun Lu,[1] Jie Luo,[1] Michael Montag,[1] Yael Diskin-Posner,[2] and David Milstein[1,]*

[1]*Department of Molecular Chemistry and Materials Science, Weizmann Institute of Science, Rehovot, 76100, Israel.*
[2]*Department of Chemical Research Support, Weizmann Institute of Science, Rehovot 76100, Israel.*

*Correspondence to: david.milstein@weizmann.ac.il

Table of Contents:

**1**	General Considerations
**1-3**	Single Crystal X-ray Diffraction Analysis of Product **1**
**3-5**	General Experimental Procedures
**5-7**	Control Experiments
**7-10**	Mechanism Studies
**11-16**	Analytical Data of Products
**17-33**	NMR Spectra of Products
**34-103**	Computational Details
**104-105**	Supplementary References

1. General Considerations

All reactions investigated in the current work were performed under an atmosphere of purified nitrogen in an MBraun glovebox, or by using standard Schlenk techniques. All commercially available reagents were used as received. Secondary alcohol substrates of **1**, **2**, **6**, **12** and **14**, as well as all ketone substrates, were commercially available. Secondary alcohol substrates of **3-5**, **7-11**, **13** and **15** were synthesized as described below. All organic solvents were of HPLC or higher grade, and were degassed with nitrogen and stored in the glovebox over 3Å molecular sieves before use. Complexes **Mn-1**,[1] **Mn-2**,[2] **Mn-3**,[3] **Mn-4**,[4] and **Mn-5**[5] were synthesized according to previously reported procedures.

For each catalytic reaction examined in this work, substrate conversions and product yields were determined either *in situ* by ^{1}H NMR spectroscopy, employing dibromomethane as an internal standard, or after isolation, following column chromatography. Solution NMR spectra were recorded using Bruker Avance NEO 300 MHz, Avance NEO 400 MHz, or Avance III HD 500 MHz spectrometers at 293 K. ^{1}H and ^{13}C NMR chemical shifts are reported in ppm relative to tetramethylsilane, and are referenced to the respective NMR signal of the solvent. ^{31}P NMR chemical shifts are reported in ppm relative to H_3PO_4 and referenced to an external sample of 85% aqueous phosphoric acid ($\delta = 0.0$ ppm). Abbreviations used in the description of NMR data are as follows: s, singlet; d, doublet; t, triplet; q, quartet; m, multiplet; br, broad.

Gas chromatography (GC) analysis was performed with an HP 6890 chromatograph, equipped with a thermal conductivity detector (TCD), using helium as the carrier gas. Gas chromatography-mass spectrometry (GC-MS) analysis was carried out on an Agilent Technologies 7820A chromatograph equipped with a 5975 Series Mass Selective Detector, using helium as the carrier gas. Liquid chromatography–mass spectrometry (LC-MS) analysis was carried out on a Waters Acquity liquid chromatography system equipped with a PDA Detector (210 and 700 nm) and a Waters QDa mass detector with an electrospray ionization (ESI) and a mass range of 85–1250 m/z. Analytical TLC was performed on Merck silica gel 60 F254 plates. Flash chromatography columns were packed with 200-300 mesh silica gel.

In the present work, room temperature typically ranges from 22 to 25 °C. Throughout the manuscript and supplementary information, all of the cited reaction temperatures are nominal, that is, each of them refers to the temperature of the oil bath used to heat the reaction vessel. The nominal temperature often differs from the actual temperature of the reaction mixture, due to solvent evaporation and reflux.

2. Single Crystal X-ray Diffraction Analysis of Product 1

Diffraction data from a single crystal of **1** were collected on a Rigaku Synergy-R diffractometer equipped with a HyPix-Arc 150 detector, and a CuKα source

(λ=1.54184Å). The data were processed with CrysAlis^{PRO}.[6] The structure was solved with SHELXT,[7] and refined using SHELXL,[8] with full matrix least-squares based on F^2. All structure solution and refinement programs were implemented in the Olex-2 GUI.[9] All non-hydrogen atoms were refined with anisotropic displacement coefficients, whereas hydrogen atoms were placed in calculated positions and refined in riding mode. See Table 1 for crystallographic details.

Table S1. Crystal data and structure refinement parameters for product **1**.

Compound	**1**
CCDC number	2446958
Crystal description	Colorless prism
Empirical formula	$C_{18}H_{16}O_2$
Formula weight (g/mol)	264.31
T (K)	100.0(2)
Source	CuKα
Wavelength (Å)	1.54184
Crystal system	Monoclinic
Space group	$P\ 2_1/n$
a (Å)	5.79105(11)
b (Å)	10.17928(15)
c (Å)	23.0525(3)
α (°)	90
β (°)	90.0205(14)
γ (°)	90
Volume (Å^3)	1358.91(4)
Z	4
Density, calculated (mg/m^3)	1.292
Absorption coefficient (mm^{-1})	0.658
Theta range for data collection (°)	3.835 to 74.500
Reflections collected (unique)	58066 (2734)
R_{int}	0.0467
Completeness to θ (%)	98.2
Data/restraints/parameters	2734/0/184

Goodness-of-fit on F^2	1.037
Final R_1 and wR_2 indices [I>2σ(I)]	R_1 = 0.0366, wR_2 = 0.0902
R_1 and wR_2 indices (all data)	R_1 = 0.0439, wR_2 = 0.0948
Largest diff. peak and hole (e/Å^3)	0.256 and -0.191

3. General Experimental Procedures

Synthesis of secondary alcohol substrates from ketones

The following procedure was used for the synthesis of secondary alcohol substrates of **3-5**, **7-11**, **13** and **15**. A 20 mL glass vial was charged with 2.0 mmol of ketone, methanol (5.0 mL) and a Teflon-coated magnetic stirring bar, and the vial was cooled to 0 °C in an ice bath. Subsequently, sodium borohydride (5.4 mmol, 2.7 equiv *vs* ketone) was added to the cold solution, and the resulting mixture was stirred for 5 h under air. The progress of the reaction was monitored using TLC. Upon completion, 5.0 mL of H_2O were added to the reaction mixture to quench the excess sodium borohydride, and the mixture was extracted with dichloromethane (2 × 2.0 mL). The extracts were then dried with anhydrous Na_2SO_4, combined into a single solution, and the solvent was evaporated under reduced pressure to obtain the corresponding secondary alcohol. The purity of the product was determined through NMR analysis, and was typically greater than 98%.

Conversion of 1-phenylethanol and ethylene glycol into 2-hydroxy-5-methyl-3,5-diphenylcyclopent-2-en-1-one using Mn-5 in a closed system

In an N_2-filled glovebox, a 100 mL glass pressure tube was charged with 1-phenylethanol (22.0 μL, 0.2 mmol), ethylene glycol (12.0 μL, 0.2 mmol), **Mn-5** (1.3 mg, 0.002 mmol, 1.0 mol% *vs* 1-phenylethanol), KOH (22.4 mg, 0.4 mmol, 2.0 equiv *vs* 1-phenylethanol), toluene (2.0 mL) and a Teflon-coated magnetic stirring bar. The tube was then sealed and removed from the glovebox, and the reaction mixture was stirred at 130 °C for 20 h. The mixture was subsequently allowed to cool to room temperature, and acidified by adding a 3 M aqueous solution of HCl (0.2 mL, 0.6 mmol). The mixture was then extracted with ethyl acetate (3 × 2.0 mL), and the extracts were dried with anhydrous Na_2SO_4. The extracts were combined and subjected to flash column chromatography over silica gel, using a 15:1 petroleum ether/ethyl acetate mixture as the eluent. The fraction containing the desired product was then placed under reduced pressure to remove the solvent and give the pure product in 40% yield.

Conversion of 1-phenylethanol and ethylene glycol into 2-hydroxy-5-methyl-3,5-diphenylcyclopent-2-en-1-one using Mn-5 in an open system

The open system procedure was conducted using a 30 mL Schlenk tube under N_2 flow, but was otherwise identical to the procedure carried out in a sealed pressure tube (see above). The desired product was obtained in 79% yield.

Conversion of acetophenone and ethylene glycol into 2-hydroxy-5-methyl-3,5-diphenylcyclopent-2-en-1-one using Mn-5 in an open system

The open system procedure involving acetophenone was identical to the one involving 1-phenylethanol (see above). The desired product was obtained in 84% yield.

The reaction between aliphatic ketones and ethylene glycol using Mn-5 in an open system

The open system procedure involving aliphatic ketone was identical to the one involving 1-phenylethanol (see above). The products were analyzed using NMR spectroscopy and GC-MS.

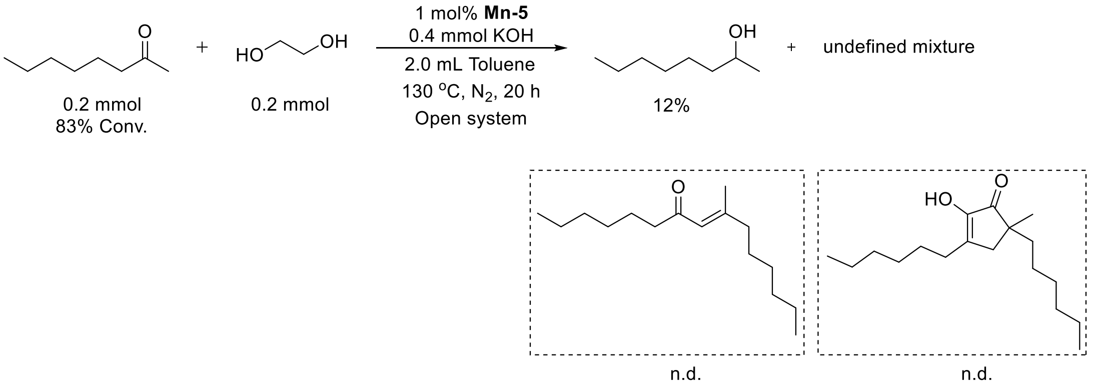

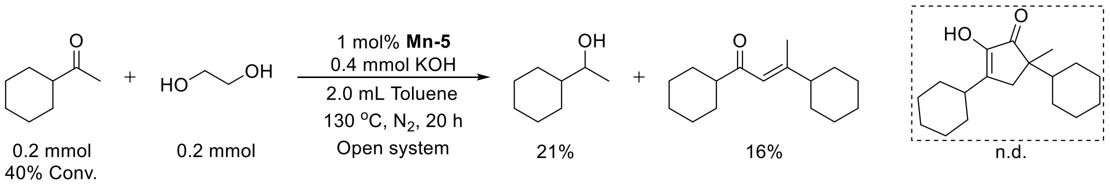

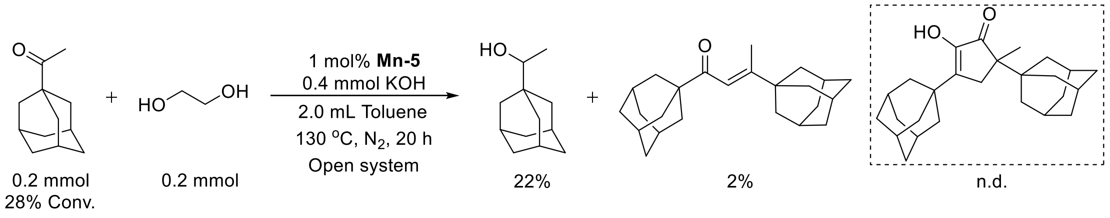

Conversion of gram-scale 1-phenylethanol and ethylene glycol into 2-hydroxy-5-methyl-3,5-diphenylcyclopent-2-en-1-one using Mn-5 in an open system

In an N_2-filled glovebox, a 250 mL glass flask was charged with 1-phenylethanol (732 mg, 6.0 mmol), ethylene glycol (372 mg, 6.0 mmol), **Mn-5** (40.4 mg, 0.06 mmol, 1.0 mol% *vs* 1-phenylethanol), KOH (672 mg, 12.0 mmol, 2.0 equiv *vs* 1-phenylethanol), toluene (60.0 mL) and a Teflon-coated magnetic stirring bar. The flask was then sealed and removed from the glovebox, and the reaction mixture was stirred under N_2 flow at 130 °C for 20 h. The mixture was subsequently allowed to cool to room temperature, and acidified by adding a 3 M aqueous solution of HCl (5.0 mL, 15.0 mmol). The mixture was then extracted with ethyl acetate (3 × 30.0 mL), and the extracts were dried with anhydrous Na_2SO_4. The extracts were combined and subjected to flash column chromatography over silica gel, using a 15:1 petroleum ether/ethyl acetate mixture as the eluent. The fraction containing the desired product was then placed under reduced pressure to remove the solvent and give the pure product in 65% yield (515 mg).

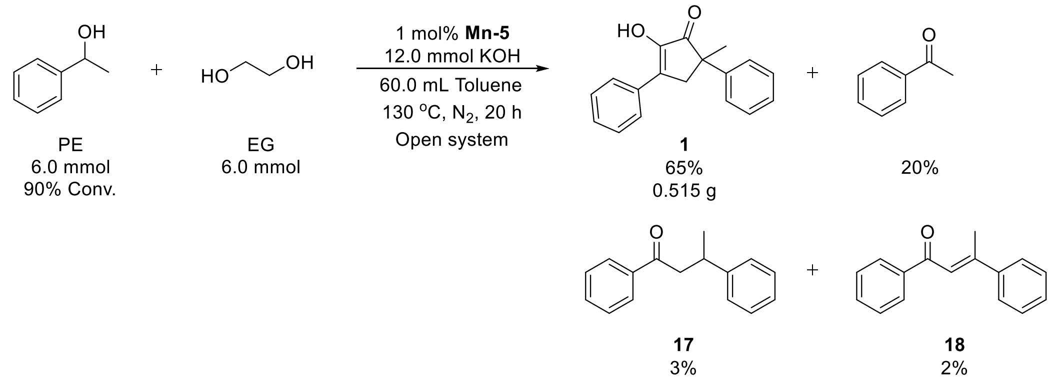

4. Control Experiments

Annulation reaction in a closed system

In an N_2-filled glovebox, a 100 mL glass pressure tube was charged with 1-phenylethanol (22.0 μL, 0.2 mmol), ethylene glycol (12.0 μL, 0.2 mmol), **Mn-5** (1.3 mg, 0.002 mmol, 1.0 mol% *vs* 1-phenylethanol), KOH (22.4 mg, 0.4 mmol, 2.0 equiv *vs* 1-phenylethanol), toluene (2.0 mL) and a Teflon-coated magnetic stirring bar. The tube was then sealed and removed from the glovebox, and the reaction mixture was stirred at 130 °C for 20 h, after which it was allowed to cool to room temperature. The pressure tube was then connected, *via* flexible plastic tubing, to an inverted graduated cylinder filled with silicone oil, and the gaseous phase above the reaction mixture was allowed to flow into the cylinder and displace the oil. Once the gas bubbling had stopped, the pressure tube was sealed and the collected gas was subjected to GC-TCD analysis to detect H_2. The liquid reaction mixture was analyzed by ^{1}H NMR

spectroscopy to detect H_2O. Subsequently, the reaction mixture was acidified by adding a 3 M aqueous solution of HCl (0.2 mL, 0.6 mmol), and was extracted with ethyl acetate (3 × 2.0 mL). The extracts were dried with anhydrous Na_2SO_4, combined into a single solution, and then analyzed *in situ* by ^1H and ^{13}C NMR spectroscopy.

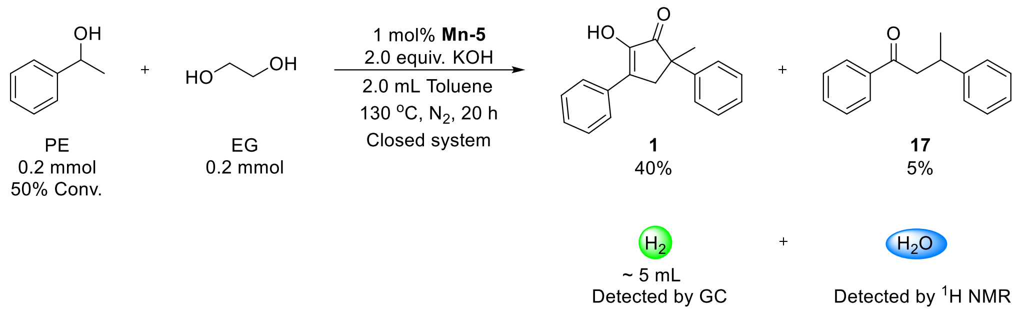

Figure S1. Conversion of 1-phenylethanol and ethylene glycol into 2-hydroxy-5-methyl-3,5-diphenylcyclopent-2-en-1-one using **Mn-5** in a closed system.

Gas chromatography following the annulation reaction in a closed system

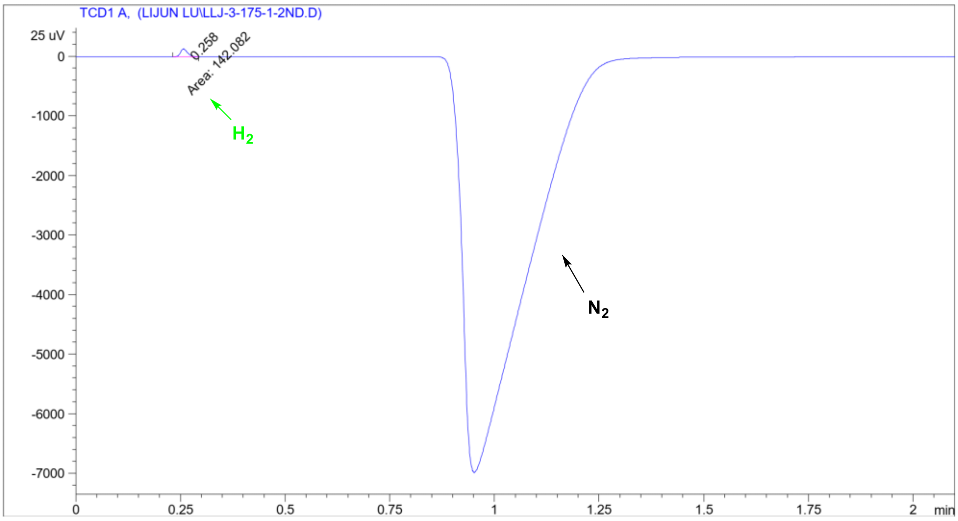

Peak #	RetTime [min]	Type	Width [min]	Area [25 uV*s]	Height [25 uV]	Area %
1	0.258	MM	0.0185	142.08205	128.24263	1.000e2
Totals :				142.08205	128.24263	

Figure S2. GC-TCD chromatogram for a sample of gas collected after the annulation reaction of PE and EG using **Mn-5** as catalyst in a closed system.

NMR spectra following the annulation reaction in a closed system

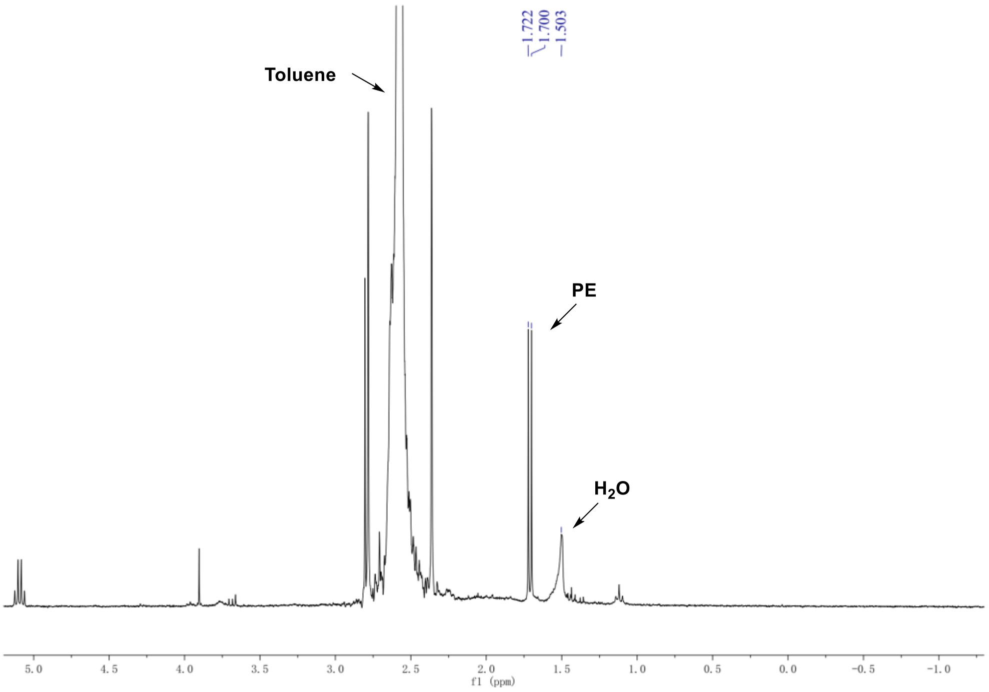

Figure S3. ^{1}H NMR spectrum (300 MHz) of the reaction mixture after the annulation reaction of PE and EG using **Mn-5** as catalyst in a closed system. A sample of the reaction mixture was dissolved in $CDCl_3$.

Conversion of 1,3-diphenyl-2-buten-1-one (18) and ethylene glycol into 2-hydroxy-5-methyl-3,5-diphenylcyclopent-2-en-1-one using Mn-5 in an open system

The open system procedure involving 1,3-diphenyl-2-buten-1-one (**18**) was identical to the one involving 1-phenylethanol (see above General Experimental Procedures). The desired product was obtained in 55% yield.

5. Mechanism Studies

Conversion of 1-phenylethanol and ethylene glycol using Mn-5-^{i}Pr with P^{i}Pr$_2$ group in an open system

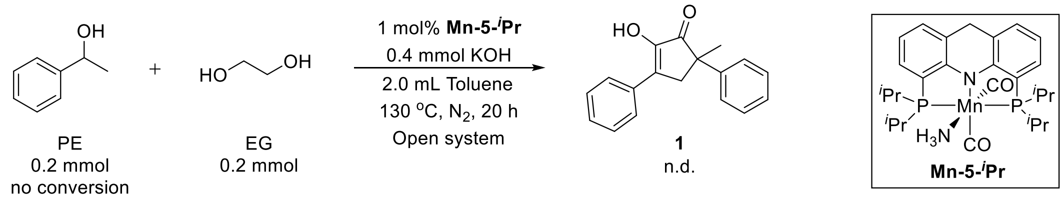

DFT calculation of 1-phenylethanol dehydrogenation catalyzed by Mn-5-^{i}Pr with P^{i}Pr$_2$ group

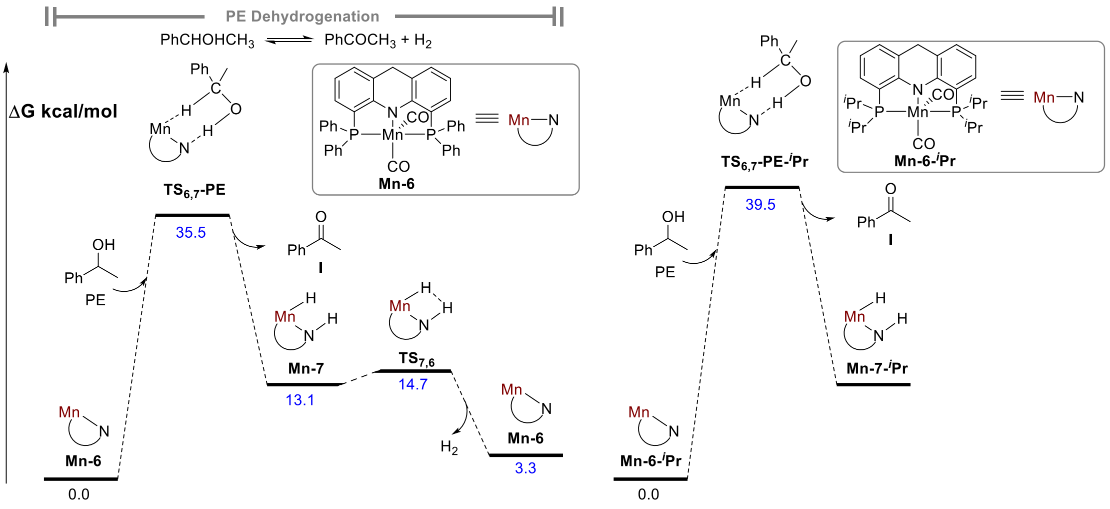

Structure comparison of 1-phenylethanol dehydrogenation transition state of TS$_{6,7}$-PE and TS$_{6,7}$-PE-iPr

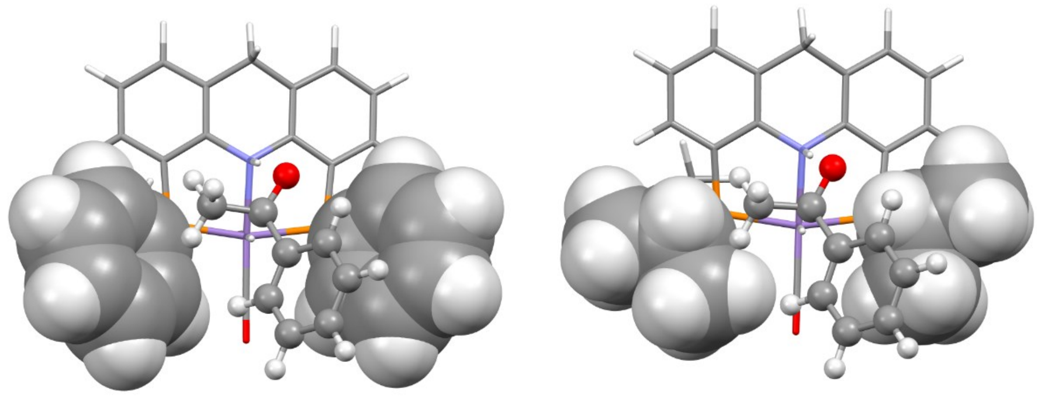

TS$_{6,7}$-PE **TS$_{6,7}$-PE-^{i}Pr**

Conversion of 1-phenylethanol and ethylene glycol into 2-hydroxy-5-methyl-3,5-diphenylcyclopent-2-en-1-one using Mn-2-Ph with PPh_2 group in an open system

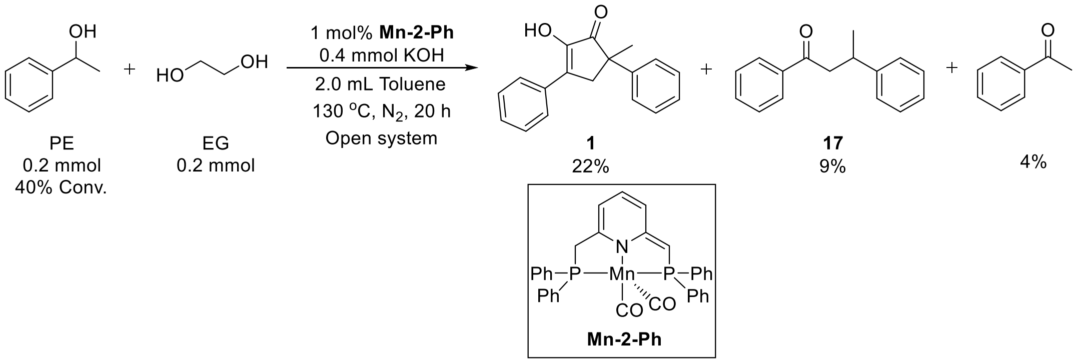

Sampling experiment of 1-phenylethanol and ethylene glycol into 2-hydroxy-5-methyl-3,5-diphenylcyclopent-2-en-1-one using Mn-5 in an open system

In an N_2-filled glovebox, a 100 mL double-necked glass flask was charged with 1-phenylethanol (44.0 µL,, 0.4 mmol), ethylene glycol (24.0 µL, mg, 0.4 mmol), **Mn-5** (2.6 mg, 0.004 mmol, 1.0 mol% *vs* 1-phenylethanol), KOH (44.8 mg, 0.8 mmol, 2.0 equiv *vs* 1-phenylethanol), 1,3,5-Trimethoxybenzene (16.8 mg, 0.1 mmol, 0.25 equiv *vs* 1-phenylethanol), toluene-d_8 (4.0 mL) and a Teflon-coated magnetic stirring bar. The flask was then sealed and removed from the glovebox, and the reaction mixture was stirred under N_2 flow at 130 °C. At specific time points, approximately 0.1 mL of solution was withdrawn using a long-needle syringe. The liquid reaction mixture was analyzed by ^1H NMR spectroscopy and GC-MS.

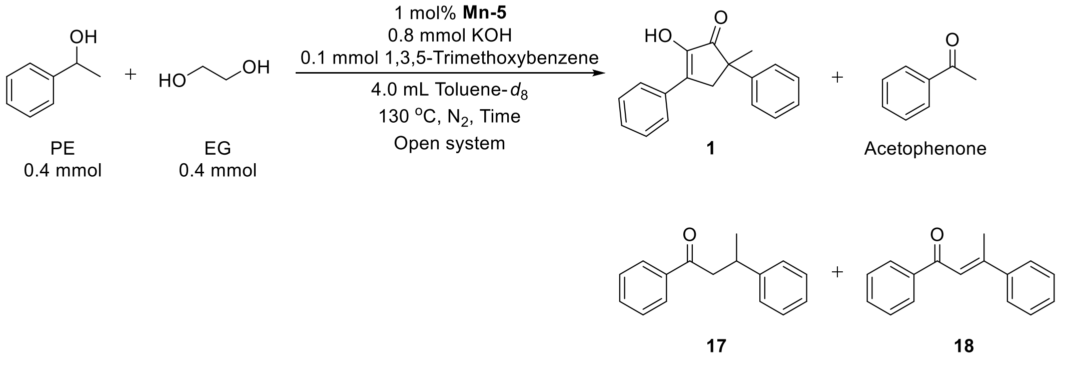

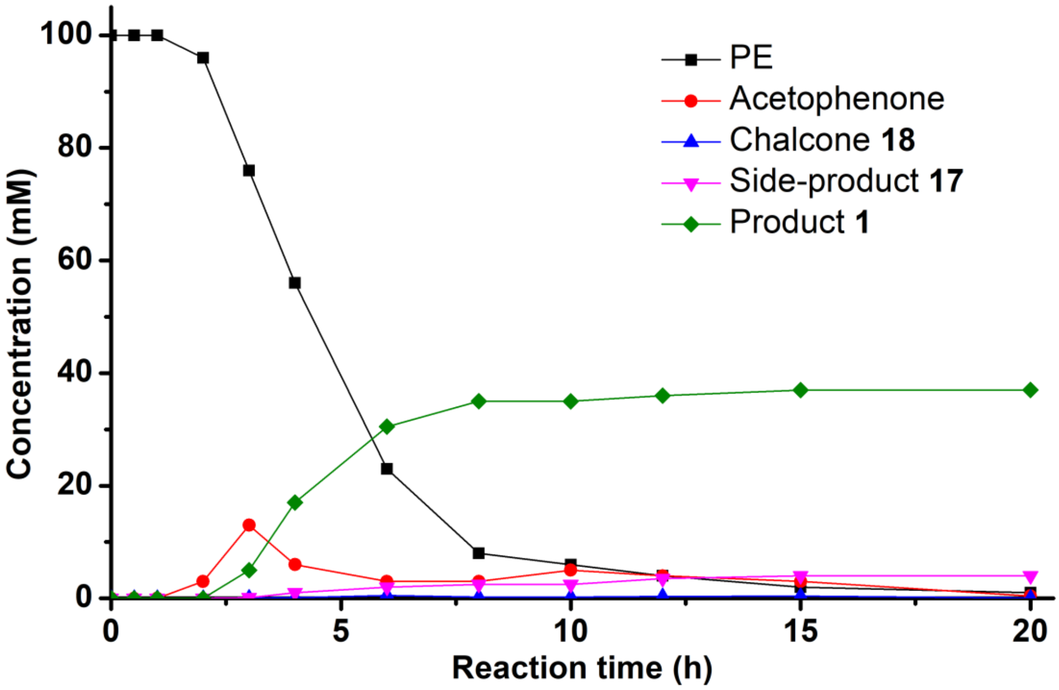

Reaction Time (h)	PE		Acetophenone		Chalcone **18**		Side-product **17**		Product **1**	
	Conv. (%)	Conc. (mM)	Yield (%)	Conc. (mM)	Yield (%)	Conc. (mM)	Yield (%)	Conc. (mM)	Yield (%)	Conc. (mM)
0	0	100	n.d.	n.d.	n.d.	n.d.	n.d.	n.d.	n.d.	n.d.
0.5	0	100	n.d.	n.d.	n.d.	n.d.	n.d.	n.d.	n.d.	n.d.
1	0	100	n.d.	n.d.	n.d.	n.d.	n.d.	n.d.	n.d.	n.d.
2	4	96	3	3	n.d.	n.d.	n.d.	n.d.	n.d.	n.d.
3	24	76	13	13	0.1	0.05	0.2	0.1	10	5
4	44	56	6	6	0.2	0.1	2	1	34	17
6	77	23	3	3	1	0.5	4	2	61	30.5
8	92	8	3	3	0.5	0.25	5	2.5	70	35
10	94	6	5	5	0.5	0.25	5	2.5	70	35
12	96	4	4	4	0.7	0.35	7	3.5	72	36
15	98	2	3	3	0.8	0.4	8	4	74	37
20	99	1	0.4	0.4	0.1	0.05	8	4	74	37

6. Analytical Data for the Products of the Catalytic Annulation

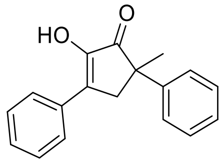

2-Hydroxy-5-methyl-3,5-diphenylcyclopent-2-en-1-one (1): white solid was obtained from the secondary alcohol in 79% isolated yield (20.9 mg), and from the ketone in 84% isolated yield (22.2 mg). ^{1}H NMR (500 MHz, $CDCl_3$) δ 7.94 (d, *J* = 7.0 Hz, 2H), 7.47 (t, *J* = 7.0 Hz, 2H), 7.43-7.40 (m, 1H), 7.33 (br, 4H), 7.24-7.21 (m, 1H), 6.04 (s, 1H), 3.30 (d, *J* = 17.0 Hz, 1H), 3.07 (d, *J* = 17.0 Hz, 1H), 1.66 (s, 3H). ^{13}C NMR (126 MHz, $CDCl_3$) δ 205.35, 145.98, 143.44, 136.18, 133.67, 129.87, 128.86, 128.15, 127.00, 126.04, 48.12, 43.25, 24.73. GC-EI-MS m/z calcd. for $[C_{18}H_{16}O_2]^+$: 264.1, found: 264.1.

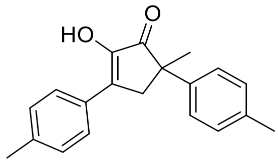

2-Hydroxy-5-methyl-3,5-di-p-tolylcyclopent-2-en-1-one (2): white solid was obtained from the secondary alcohol in 70% isolated yield (20.4 mg), and from the ketone in 76% isolated yield (22.2 mg). ^{1}H NMR (500 MHz, $CDCl_3$) δ 7.83 (d, *J* = 8.0 Hz, 2H), 7.28 (d, *J* = 8.0 Hz, 2H), 7.22 (d, *J* = 8.0 Hz, 2H), 7.13 (d, *J* = 7.5 Hz, 2H), 6.03 (s, 1H), 3.26 (d, *J* = 16.5 Hz, 1H), 3.03 (d, *J* = 17.0 Hz, 1H), 2.41 (s, 3H), 2.31 (s, 3H), 1.63 (s, 3H). ^{13}C NMR (126 MHz, $CDCl_3$) δ 205.34, 145.49, 140.55, 140.20, 136.56, 136.49, 130.98, 129.58, 129.49, 128.11, 125.91, 47.75, 43.26, 24.69, 21.70, 21.07. GC-EI-MS m/z calcd. for $[C_{20}H_{20}O_2]^+$: 292.1, found: 292.1.

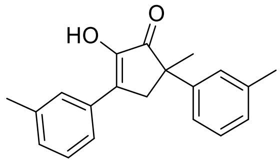

2-Hydroxy-5-methyl-3,5-di-*m*-tolylcyclopent-2-en-1-one (3): white solid was obtained from the secondary alcohol in 74% isolated yield (21.6 mg), and from the ketone in 85% isolated yield (24.8 mg). ^{1}H NMR (500 MHz, $CDCl_3$) δ 7.78-7.76 (m, 2H), 7.37 (t, *J* = 7.5 Hz, 1H), 7.25-7.21 (m, 2H), 7.14-7.12 (m, 2H), 7.06 (d, *J* = 7.5 Hz, 1H), 6.18 (s, 1H), 3.29 (d, *J* = 17.0 Hz, 1H), 3.05 (d, *J* = 16.5 Hz, 1H), 2.43 (s, 3H), 2.34 (s, 3H), 1.65 (s, 3H). ^{13}C NMR (126 MHz, $CDCl_3$) δ 205.57, 145.97, 143.44, 138.43, 136.54, 133.67, 130.68, 128.73, 128.70, 127.73, 126.73, 125.36, 123.06, 48.00, 43.45, 24.65, 21.73, 21.69. GC-EI-MS m/z calcd. for $[C_{20}H_{20}O_2]^+$: 292.1, found: 292.1.

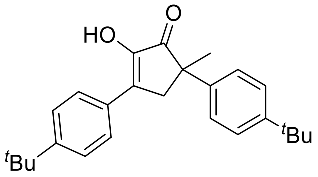

3,5-Bis(4-(*tert*-butyl)phenyl)-2-hydroxy-5-methylcyclopent-2-en-1-one (4): white solid was obtained from the secondary alcohol in 72% isolated yield (27.1 mg), and from the ketone in 79% isolated yield (29.7 mg). ^{1}H NMR (500 MHz, $CDCl_3$) δ 7.88 (d, *J* = 8.0 Hz, 2H), 7.50 (d, *J* = 8.0 Hz, 2H), 7.33 (d, *J* = 8.0 Hz, 2H), 7.25 (d, *J* = 8.0 Hz, 2H), 5.90 (s, 1H), 3.29 (d, *J* = 17.0 Hz, 1H), 3.02 (d, *J* = 17.0 Hz, 1H), 1.64 (s, 3H), 1.34 (s, 9H), 1.29 (s, 9H). ^{13}C NMR (126 MHz, $CDCl_3$) δ 205.36, 153.33, 149.75, 145.57, 140.41, 136.41, 130.96, 127.97, 125.82, 125.74, 125.68, 47.75, 43.20, 35.08, 34.53, 31.44, 31.33, 24.54. GC-EI-MS m/z calcd. for $[C_{26}H_{32}O_2]^+$: 376.2, found: 376.2.

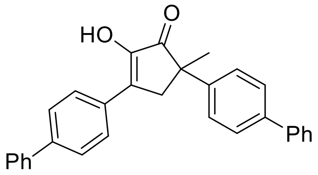

3,5-Di([1,1'-biphenyl]-4-yl)-2-hydroxy-5-methylcyclopent-2-en-1-one (5): white solid was obtained from the secondary alcohol in 60% isolated yield (25.0 mg), and from the ketone in 65% isolated yield (27.1 mg). ^{1}H NMR (500 MHz, $CDCl_3$) δ 8.04 (d, *J* = 7.5 Hz, 2H), 7.73 (d, *J* = 7.5 Hz, 2H), 7.66 (d, *J* = 7.0 Hz, 2H), 7.57 (d, *J* = 7.0 Hz, 4H), 7.48-7.34 (m, 8H), 6.14 (s, 1H), 3.38 (d, *J* = 17.0 Hz, 1H), 3.13 (d, *J* = 17.0 Hz, 1H), 1.72 (s, 3H). ^{13}C NMR (126 MHz, $CDCl_3$) δ 205.14, 146.05, 142.50, 142.48, 140.81, 140.41, 139.98, 135.99, 132.62, 129.08, 128.92, 128.64, 128.00, 127.59, 127.47, 127.45, 127.24, 127.22, 126.50, 48.00, 43.19, 24.76. LC-ESI-MS m/z calcd. for $[C_{30}H_{24}O_2+H]^+$: 417.2, found: 417.2.

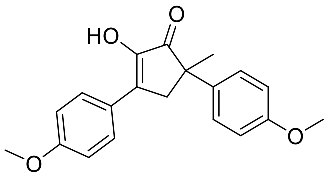

2-Hydroxy-3,5-bis(4-methoxyphenyl)-5-methylcyclopent-2-en-1-one (6): white solid was obtained from the secondary alcohol in 74% isolated yield (24.0 mg), and from the ketone in 80% isolated yield (25.9 mg). ^{1}H NMR (500 MHz, $CDCl_3$) δ 7.91 (d, *J* = 8.5 Hz, 2H), 7.25 (d, *J* = 8.5 Hz, 2H), 6.99 (d, *J* = 8.0 Hz, 2H), 6.86 (d, *J* = 8.0 Hz, 2H), 6.07 (s, 1H), 3.87 (s, 3H), 3.78 (s, 3H), 3.23 (d, *J* = 17.0 Hz, 1H), 3.01 (d, *J* = 17.0 Hz, 1H), 1.62 (s, 3H). ^{13}C NMR (126 MHz, $CDCl_3$) δ 205.06, 160.88, 158.46,

144.80, 136.53, 135.74, 129.86, 127.13, 126.56, 114.28, 114.15, 55.52, 55.43, 47.38, 43.34, 24.85. GC-EI-MS m/z calcd. for $[C_{20}H_{20}O_4]^+$: 324.1, found: 324.1.

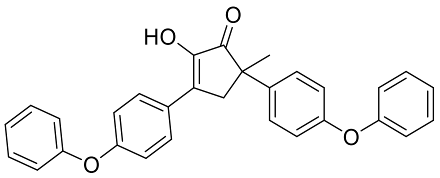

2-Hydroxy-5-methyl-3,5-bis(4-phenoxyphenyl)cyclopent-2-en-1-one (7): white solid was obtained from the secondary alcohol in 78% isolated yield (34.9 mg), and from the ketone in 80% isolated yield (35.9 mg). ^{1}H NMR (500 MHz, $CDCl_3$) δ 7.93 (d, *J* = 8.0 Hz, 2H), 7.38 (t, *J* = 7.5 Hz, 2H), 7.34-7.28 (m, 4H), 7.17 (t, *J* = 7.5 Hz, 1H), 7.11-7.07 (m, 5H), 6.99 (d, *J* = 7.5 Hz, 2H), 6.96 (d, *J* = 7.5 Hz, 2H), 6.08 (s, 1H), 3.25 (d, *J* = 17.0 Hz, 1H), 3.04 (d, *J* = 16.5 Hz, 1H), 1.65 (s, 3H). ^{13}C NMR (126 MHz, $CDCl_3$) δ 205.00, 158.99, 157.28, 156.39, 156.15, 145.29, 138.30, 135.98, 130.08, 129.93, 129.87, 128.52, 127.45, 124.21, 123.42, 119.75, 119.09, 119.01, 118.50, 47.62, 43.33, 24.91. LC-ESI-MS m/z calcd. for $[C_{30}H_{24}O_4+H]^+$: 449.2, found: 449.2.

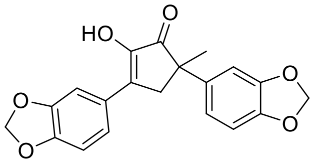

3,5-Bis(benzo[*d*][1,3]dioxol-5-yl)-2-hydroxy-5-methylcyclopent-2-en-1-one (8): light yellow solid was obtained from the secondary alcohol in 61% isolated yield (21.5 mg), and from the ketone in 65% isolated yield (22.9 mg). ^{1}H NMR (500 MHz, $CDCl_3$) δ 7.54 (s, 1H), 7.40 (d, *J* = 8.0 Hz, 1H), 6.90 (d, *J* = 8.0 Hz, 1H), 6.80-6.74 (m, 3H), 6.03 (s, 2H), 5.92 (s, 3H), 3.16 (d, *J* = 17.0 Hz, 1H), 2.97 (d, *J* = 17.0 Hz, 1H), 1.59 (s, 3H). ^{13}C NMR (126 MHz, $CDCl_3$) δ 204.68, 149.04, 148.23, 148.13, 146.49, 144.92, 137.42, 136.19, 127.99, 122.83, 119.13, 108.67, 108.49, 108.36, 106.89, 101.62, 101.22, 47.73, 43.68, 24.90. GC-EI-MS m/z calcd. for $[C_{20}H_{16}O_6]^+$: 352.1, found: 352.1.

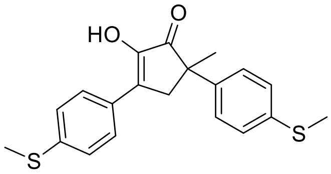

2-Hydroxy-5-methyl-3,5-bis(4-(methylthio)phenyl)cyclopent-2-en-1-one (9): light yellow solid was obtained from the secondary alcohol in 75% isolated yield (26.7 mg), and from the ketone in 83% isolated yield (29.6 mg). ^{1}H NMR (500 MHz, $CDCl_3$) δ 7.85 (d, *J* = 8.0 Hz, 2H), 7.31 (d, *J* = 8.0 Hz, 2H), 7.23 (q, *J* = 8.0 Hz, 4H), 6.10 (s,

1H), 3.22 (d, J = 17.0 Hz, 1H), 3.02 (d, J = 16.5 Hz, 1H), 2.53 (s, 3H), 2.45 (s, 3H), 1.63 (s, 3H). ^{13}C NMR (126 MHz, $CDCl_3$) δ 204.80, 145.58, 141.50, 140.42, 137.09, 136.00, 130.11, 128.42, 127.18, 126.57, 125.94, 47.71, 42.97, 24.74, 16.12, 15.32. GC-EI-MS m/z calcd. for $[C_{20}H_{20}S_2O_2]^+$: 356.1, found: 356.1.

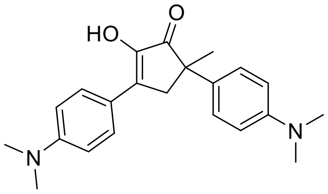

3,5-Bis(4-(dimethylamino)phenyl)-2-hydroxy-5-methylcyclopent-2-en-1-one (10): yellow solid was obtained from the secondary alcohol in 55% isolated yield (19.3 mg), and from the ketone in 65% isolated yield (22.8 mg). ^{1}H NMR (500 MHz, $CDCl_3$) δ 7.85 (d, J = 8.0 Hz, 2H), 7.20 (d, J = 8.0 Hz, 2H), 6.75 (d, J = 8.0 Hz, 2H), 6.69 (d, J = 8.0 Hz, 2H), 5.85 (s, 1H), 3.21 (d, J = 16.5 Hz, 1H), 3.04 (s, 6H), 2.96 (d, J = 17.0 Hz, 1H), 2.90 (s, 6H), 1.60 (s, 3H). ^{13}C NMR (126 MHz, $CDCl_3$) δ 204.52, 151.07, 149.36, 143.60, 137.68, 131.76, 129.55, 126.57, 121.66, 112.86, 111.70, 46.94, 43.10, 40.69, 40.15, 24.50. GC-EI-MS m/z calcd. for $[C_{22}H_{26}N_2O_2]^+$: 350.2, found: 350.2.

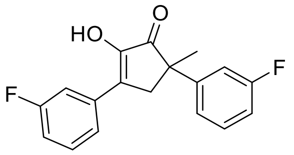

3,5-Bis(3-fluorophenyl)-2-hydroxy-5-methylcyclopent-2-en-1-one (11): white solid was obtained from the secondary alcohol in 60% isolated yield (18.0 mg), and from the ketone in 66% isolated yield (19.8 mg). ^{1}H NMR (500 MHz, $CDCl_3$) δ 7.68 (d, J = 8.5 Hz, 2H), 7.44 (q, J = 7.5 Hz, 1H), 7.29 (q, J = 7.5 Hz, 1H), 7.14-7.05 (m, 3H), 6.95 (t, J = 8.0 Hz, 1H), 6.14 (s, 1H), 3.24 (d, J = 17.0 Hz, 1H), 3.05 (d, J = 17.0 Hz, 1H), 1.65 (s, 3H). ^{13}C NMR (126 MHz, $CDCl_3$) δ 204.61, 164.08 (d, $J_{C\text{-}F}$ = 12.3 Hz), 162.13 (d, $J_{C\text{-}F}$ = 12.3 Hz), 146.43, 145.72 (d, $J_{C\text{-}F}$ = 7.1 Hz), 135.53 (d, $J_{C\text{-}F}$ = 8.2 Hz), 134.67 (d, $J_{C\text{-}F}$ = 2.8 Hz), 130.39 (d, $J_{C\text{-}F}$ = 3.2 Hz), 130.32 (d, $J_{C\text{-}F}$ = 3.0 Hz), 123.70 (d, $J_{C\text{-}F}$ = 2.9 Hz), 121.72 (d, $J_{C\text{-}F}$ = 2.8 Hz), 116.85 (d, $J_{C\text{-}F}$ = 21.5 Hz), 114.99 (d, $J_{C\text{-}F}$ = 22.7 Hz), 114.06 (d, $J_{C\text{-}F}$ = 21.0 Hz), 113.44 (d, $J_{C\text{-}F}$ = 22.6 Hz), 48.05 (d, $J_{C\text{-}F}$ = 1.5 Hz), 42.95, 24.91. ^{19}F NMR (471 MHz, $CDCl_3$) δ -133.15, -133.19. GC-EI-MS m/z calcd. for $[C_{18}H_{14}F_2O_2]^+$: 300.1, found: 300.1.

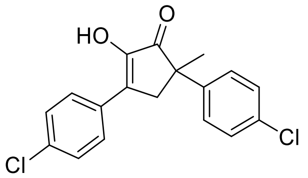

3,5-Bis(4-chlorophenyl)-2-hydroxy-5-methylcyclopent-2-en-1-one (12): light yellow solid was obtained from the secondary alcohol in 63% isolated yield (20.9 mg), and from the ketone in 72% isolated yield (23.9 mg). ^{1}H NMR (500 MHz, $CDCl_3$) δ 7.86 (d, *J* = 7.5 Hz, 2H), 7.44 (d, *J* = 7.5 Hz, 2H), 7.30-7.25 (m, 4H), 6.10 (s, 1H), 3.21 (d, *J* = 17.5 Hz, 1H), 3.03 (d, *J* = 17.0 Hz, 1H), 1.63 (s, 3H). ^{13}C NMR (126 MHz, $CDCl_3$) δ 204.65, 146.05, 141.74, 135.92, 134.82, 133.02, 131.96, 129.37, 129.16, 128.98, 127.52, 47.76, 42.89, 25.01. GC-EI-MS m/z calcd. for $[C_{18}H_{14}Cl_2O_2]^+$: 332.0, found: 332.0.

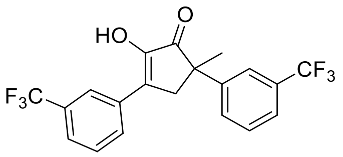

2-Hydroxy-5-methyl-3,5-bis(3-(trifluoromethyl)phenyl)cyclopent-2-en-1-one (13): white solid was obtained from the secondary alcohol in 68% isolated yield (27.2 mg), and from the ketone in 75% isolated yield (30.0 mg). ^{1}H NMR (500 MHz, $CDCl_3$) δ 8.16-8.15 (m, 2H), 7.68-7.67 (m, 1H), 7.61-7.59 (m, 2H), 7.52 (m, 2H), 7.48-7.44 (m, 1H), 6.26 (s, 1H), 3.29 (d, *J* = 16.5 Hz, 1H), 3.14 (d, *J* = 16.5 Hz, 1H), 1.70 (s, 3H). ^{13}C NMR (126 MHz, $CDCl_3$) δ 204.55, 146.72, 144.12, 134.16, 131.44 (q, J_{C-F} = 32.6 Hz), 131.30, 131.27 (q, J_{C-F} = 32.2 Hz), 129.65, 129.44, 126.37 (q, J_{C-F} = 3.7 Hz), 124.61 (q, J_{C-F} = 3.8 Hz), 124.17 (q, J_{C-F} = 273.1 Hz), 124.13 (q, J_{C-F} = 3.8 Hz), 124.08 (q, J_{C-F} = 273.1 Hz), 122.92 (q, J_{C-F} = 3.8 Hz), 48.18, 42.75, 25.37. ^{19}F NMR (471 MHz, $CDCl_3$) δ -63.54, -63.79. GC-EI-MS m/z calcd. for $C_{20}H_{14}F_6O_2$ $[M]^+$: 400.1, found: 400.1.

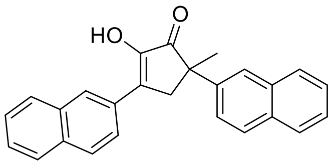

2-Hydroxy-5-methyl-3,5-di(naphthalen-2-yl)cyclopent-2-en-1-one (14): white solid was obtained from the secondary alcohol in 65% isolated yield (23.7 mg), and from the ketone in 78% isolated yield (28.4 mg). ^{1}H NMR (500 MHz, $CDCl_3$) δ 8.35 (s, 1H), 8.17 (d, *J* = 8.5 Hz, 1H), 7.93-7.80 (m, 7H), 7.54-7.41 (m, 5H), 6.24 (s, 1H), 3.52 (d, *J* = 16.5 Hz, 1H), 3.28 (d, *J* = 17.0 Hz, 1H), 1.81 (s, 3H). ^{13}C NMR (126 MHz, $CDCl_3$) δ 205.33, 146.44, 140.72, 136.38, 133.89, 133.46, 133.33, 132.41, 131.27, 129.00, 128.78, 128.47, 128.22, 127.94, 127.87, 127.59, 127.40, 126.71, 126.41, 126.10, 125.44, 124.67, 124.47, 48.38, 43.32, 24.83. GC-EI-MS m/z calcd. for $[C_{26}H_{20}O_2]^+$: 364.1, found: 364.1.

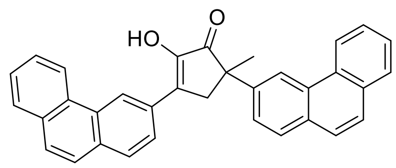

2-Hydroxy-5-methyl-3,5-di(phenanthren-3-yl)cyclopent-2-en-1-one (15): light yellow solid was obtained from the secondary alcohol in 61% isolated yield (28.3 mg), and from the ketone in 72% isolated yield (33.4 mg). ^{1}H NMR (500 MHz, $CDCl_3$) δ 9.32 (s, 1H), 8.77 (s, 2H), 8.71 (d, *J* = 8.0 Hz, 1H), 8.24 (d, *J* = 8.0 Hz, 1H), 7.98 (d, *J* = 8.5 Hz, 1H), 7.92 (d, *J* = 8.0 Hz, 1H), 7.88 (t, *J* = 7.0 Hz, 2H), 7.82 (d, *J* = 9.0 Hz, 1H), 7.77 (d, *J* = 9.0 Hz, 1H), 7.75-7.69 (m, 3H), 7.67-7.62 (m, 2H), 7.60-7.58 (m, 2H), 6.34 (s, 1H), 3.67 (d, *J* = 17.0 Hz, 1H), 3.43 (d, *J* = 16.5 Hz, 1H), 1.93 (s, 3H). ^{13}C NMR (126 MHz, $CDCl_3$) δ 205.27, 146.46, 141.63, 136.56, 132.91, 132.42, 131.77, 130.98, 130.59, 130.38, 130.36, 129.30, 129.06, 128.94, 128.78, 128.63, 127.21, 127.15, 126.82, 126.72, 126.63, 126.51, 126.09, 124.98, 122.87, 122.84, 119.83, 48.75, 43.73, 25.35. LC-ESI-MS m/z calcd. for $[C_{34}H_{24}O_2+H]^+$: 465.2, found: 365.2.

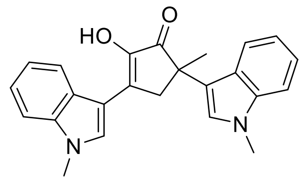

2-Hdroxy-5-methyl-3,5-bis(1-methyl-1H-indol-3-yl)cyclopent-2-en-1-one (16): light yellow solid was obtained from the ketone in 60% isolated yield (22.2 mg). ^{1}H NMR (500 MHz, $CDCl_3$) δ 8.02 (s, 1H), 7.96 (d, *J* = 8.0 Hz, 1H), 7.40 (d, *J* = 8.0 Hz, 1H), 7.33 (d, *J* = 8.0 Hz, 1H), 7.26-7.22 (m, 2H), 7.14-7.13 (m, 2H), 7.02 (s, 1H), 6.95 (t, *J* = 7.5 Hz, 1H), 5.99 (s, 1H), 3.84 (s, 3H), 3.76 (d, *J* = 17.0 Hz, 1H), 3.70 (s, 3H), 3.37 (d, *J* = 16.5 Hz, 1H), 1.69 (s, 3H). ^{13}C NMR (126 MHz, $CDCl_3$) δ 203.23, 142.58, 137.75, 137.45, 136.14, 133.49, 126.79, 126.54, 126.15, 122.74, 121.78, 121.60, 121.19, 119.75, 119.25, 117.40, 110.33, 110.04, 109.62, 44.32, 42.97, 33.52, 32.86, 25.30. LC-ESI-MS m/z calcd. for $[C_{24}H_{22}N_2O_2+H]^+$: 371.2, found: 371.2.

7. NMR Spectra of the Products of the Catalytic Annulation

Product 1

^{1}H NMR (500 MHz, $CDCl_3$)

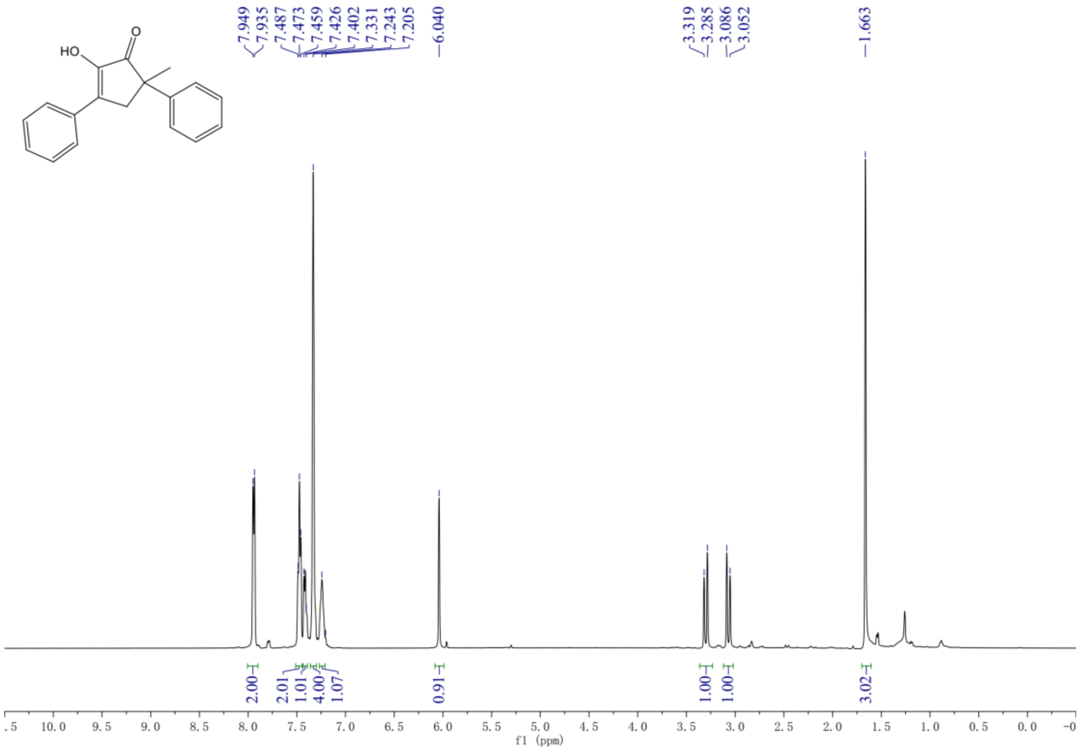

^{13}C{^{1}H NMR (126 MHz, $CDCl_3$)

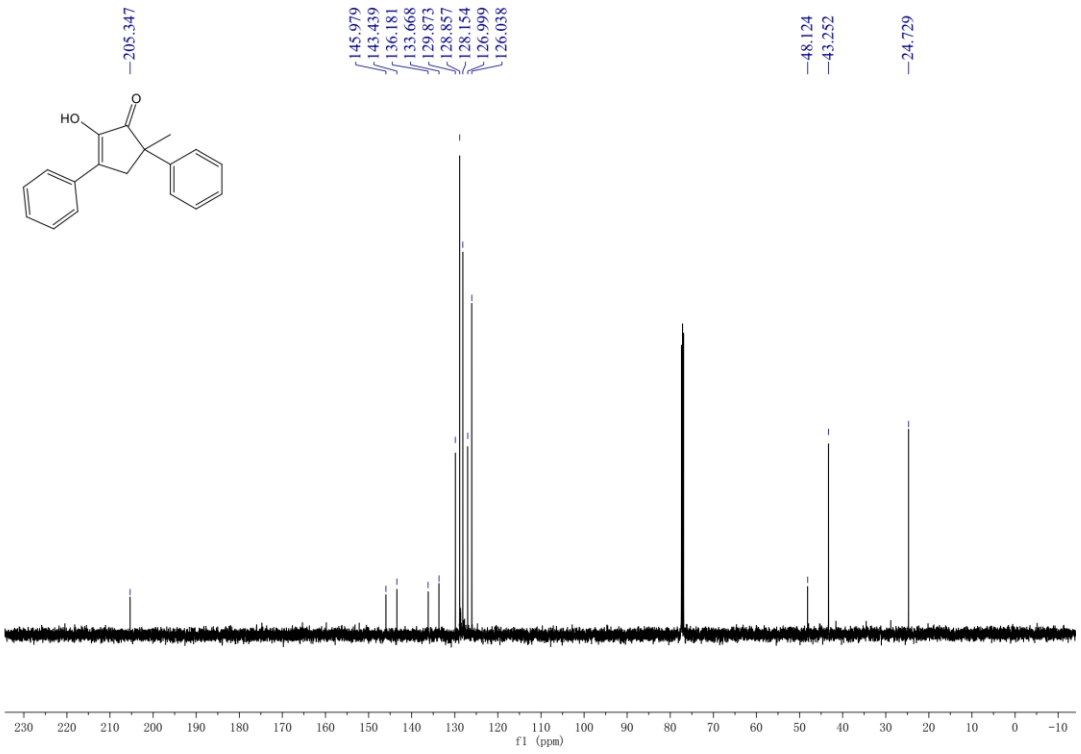

Product 2

^1H NMR (500 MHz, $CDCl_3$)

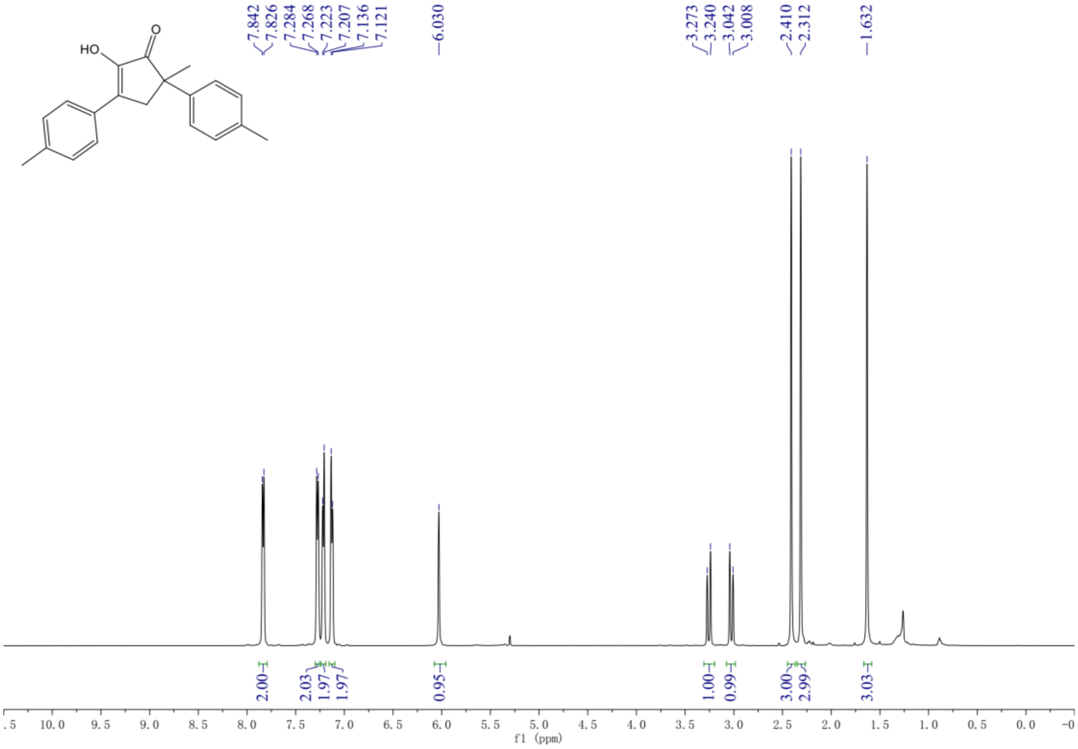

$^{13}C\{^1H\}$ NMR (126 MHz, $CDCl_3$)

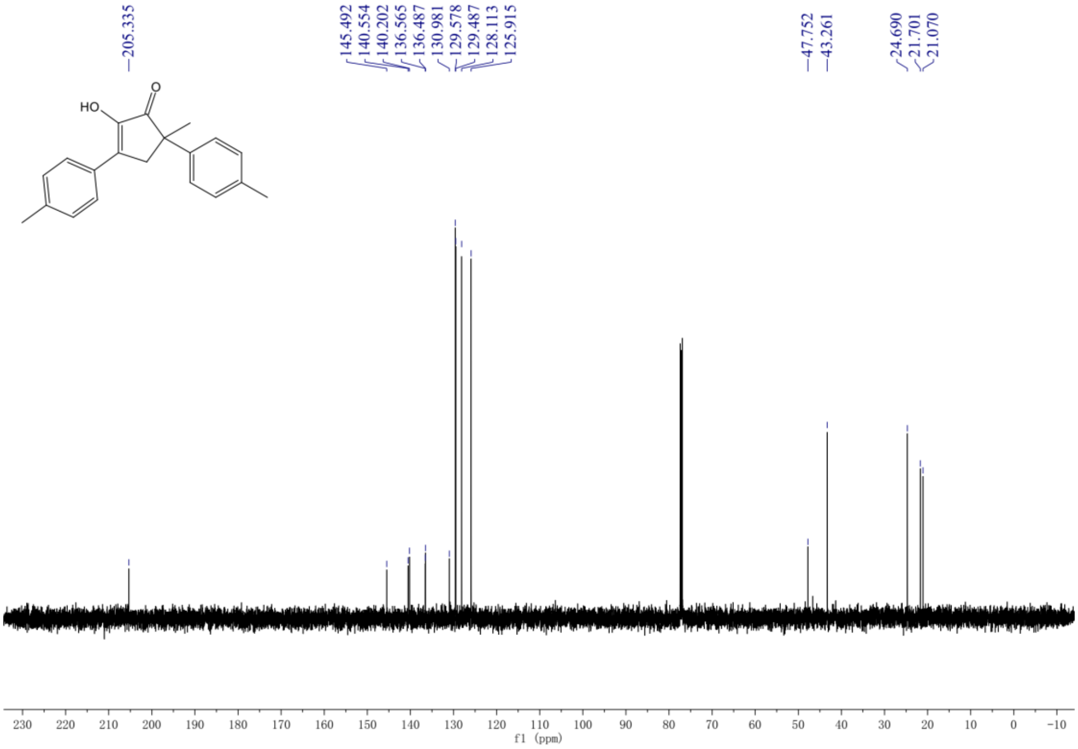

Product 3

^{1}H NMR (500 MHz, $CDCl_3$)

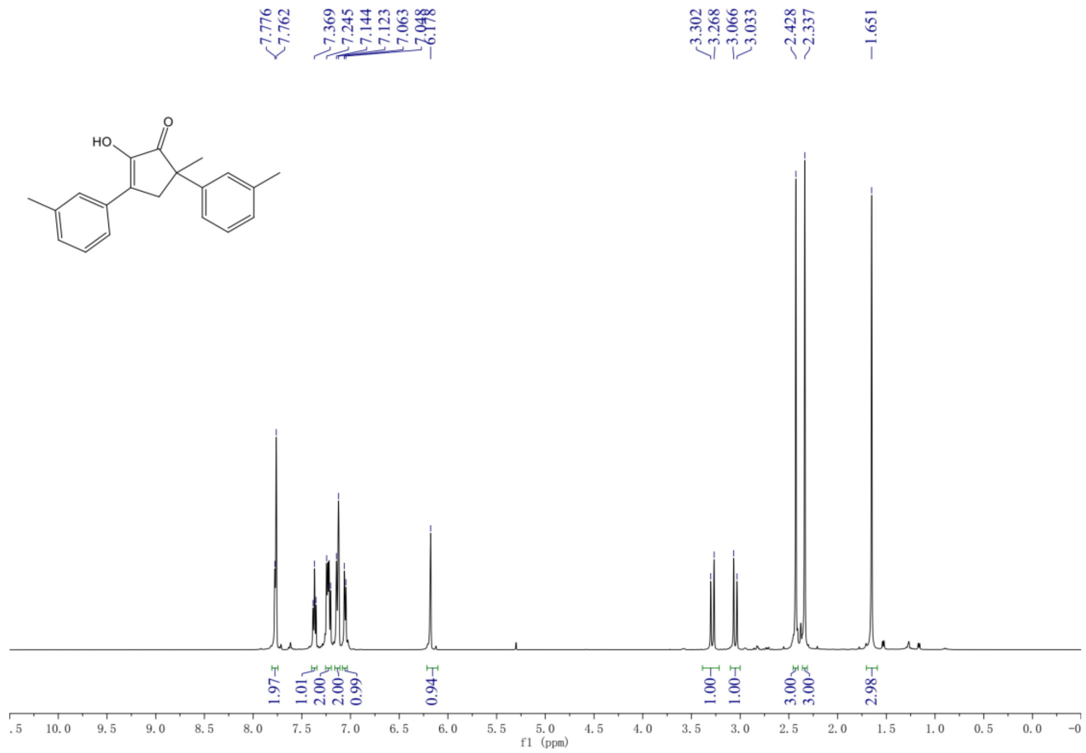

$^{13}C\{^{1}H\}$ NMR (126 MHz, $CDCl_3$)

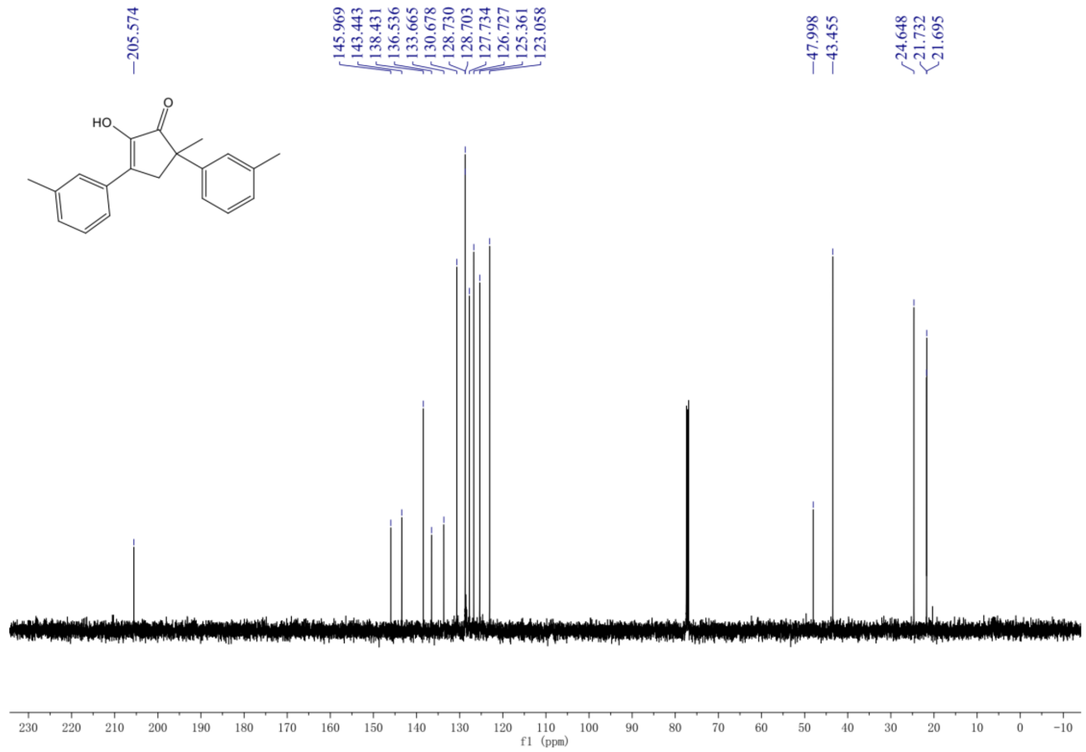

Product 4

^{1}H NMR (500 MHz, $CDCl_3$)

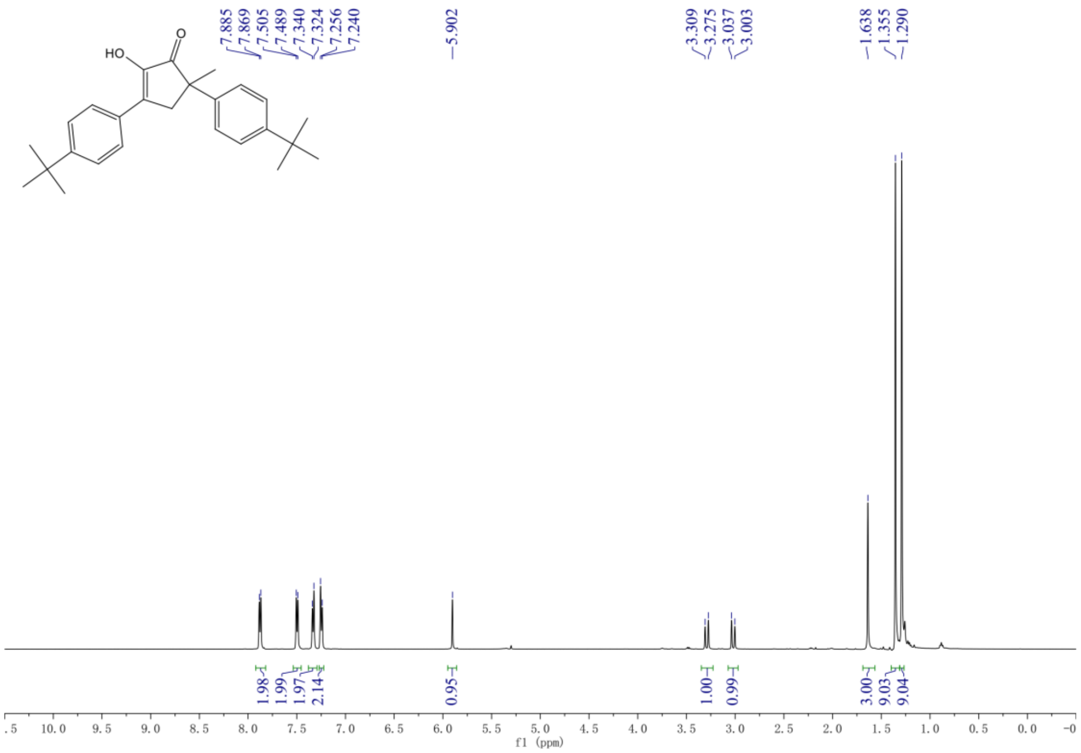

$^{13}C\{^{1}H\}$ NMR (126 MHz, $CDCl_3$)

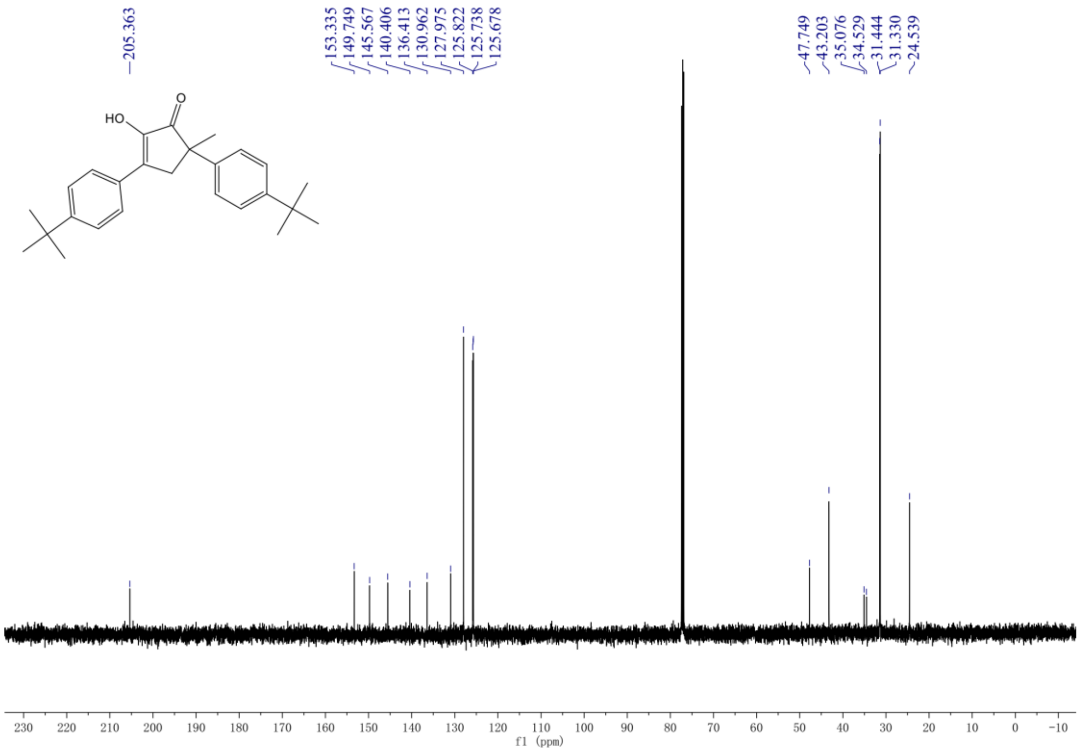

Product 5

^{1}H NMR (500 MHz, $CDCl_3$)

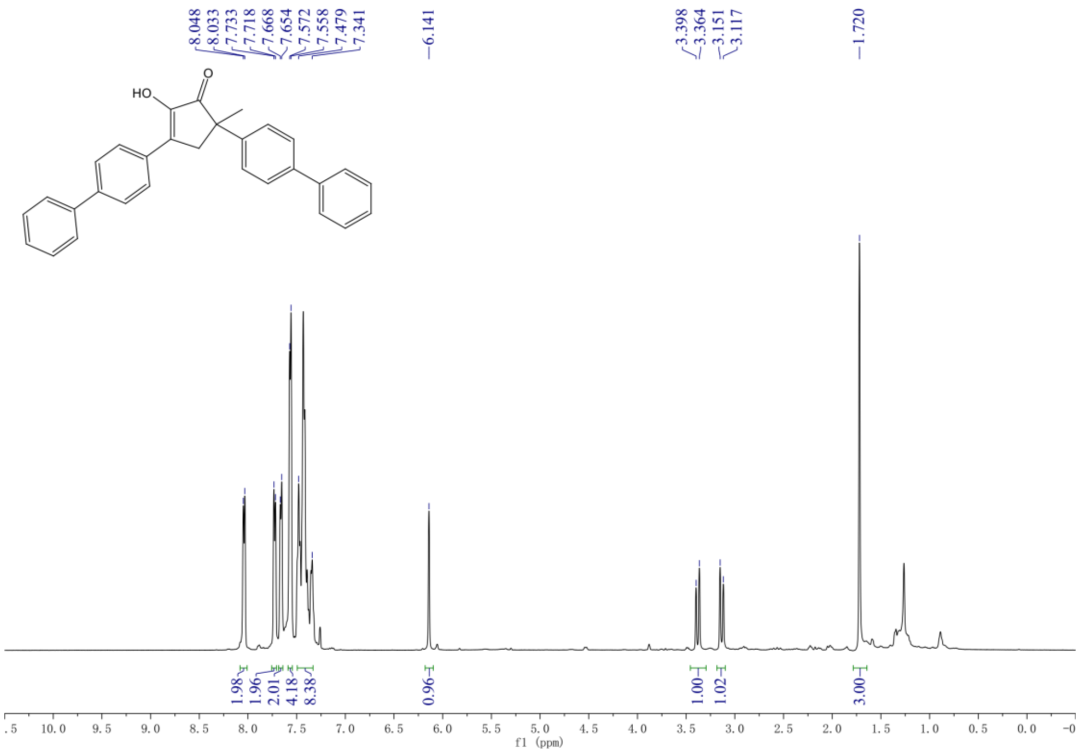

^{13}C{^{1}H} NMR (126 MHz, $CDCl_3$)

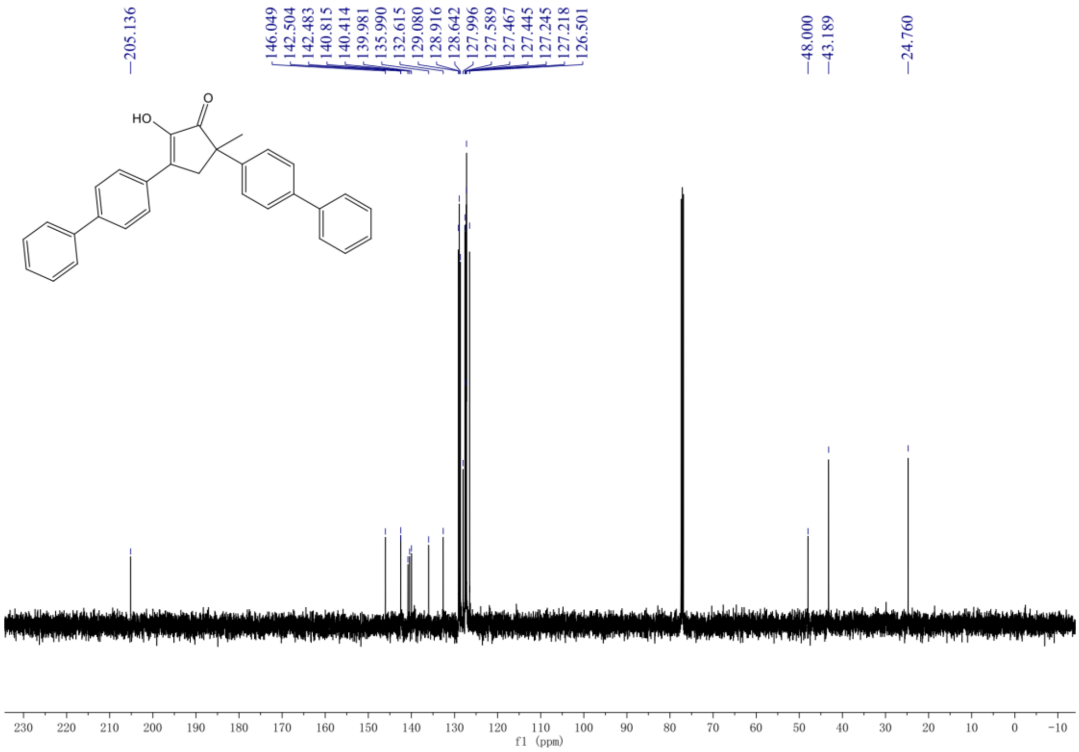

Product 6

^{1}H NMR (500 MHz, $CDCl_3$)

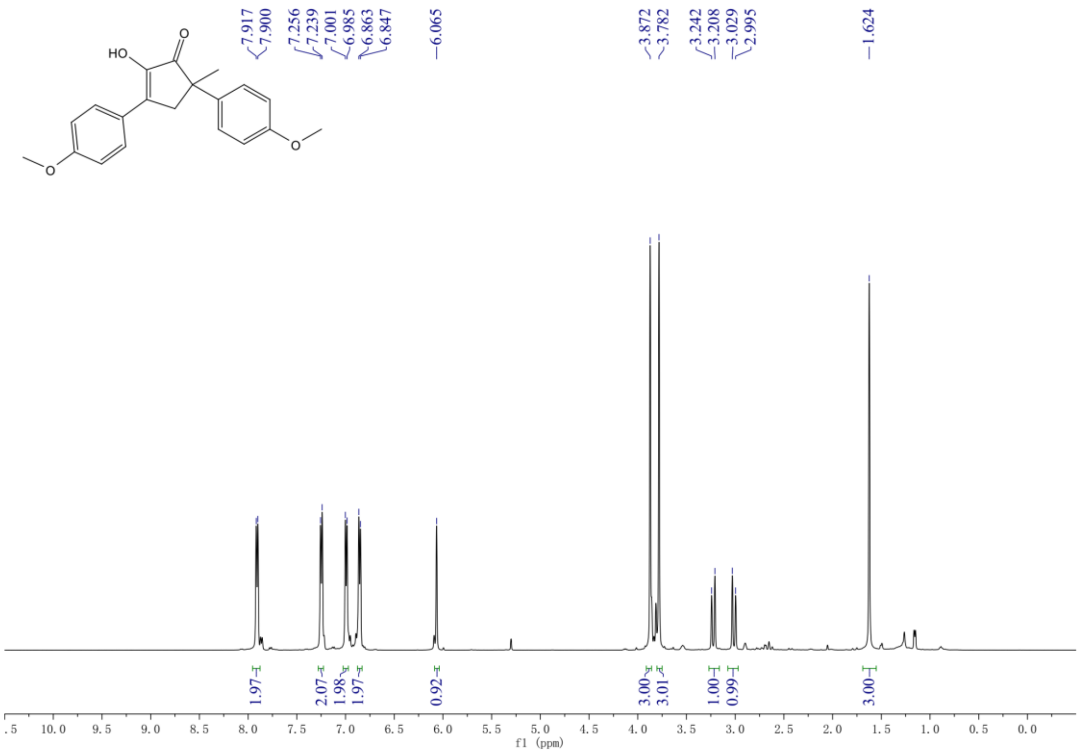

^{13}C{^{1}H} NMR (126 MHz, $CDCl_3$)

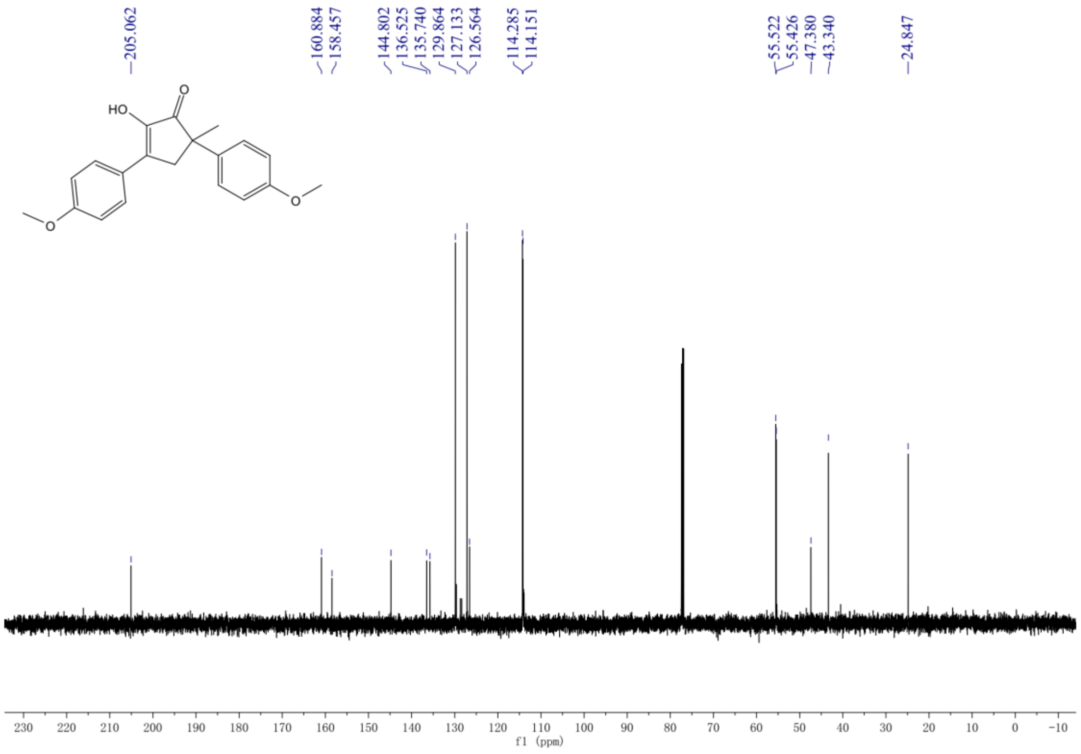

Product 7

^{1}H NMR (500 MHz, $CDCl_3$)

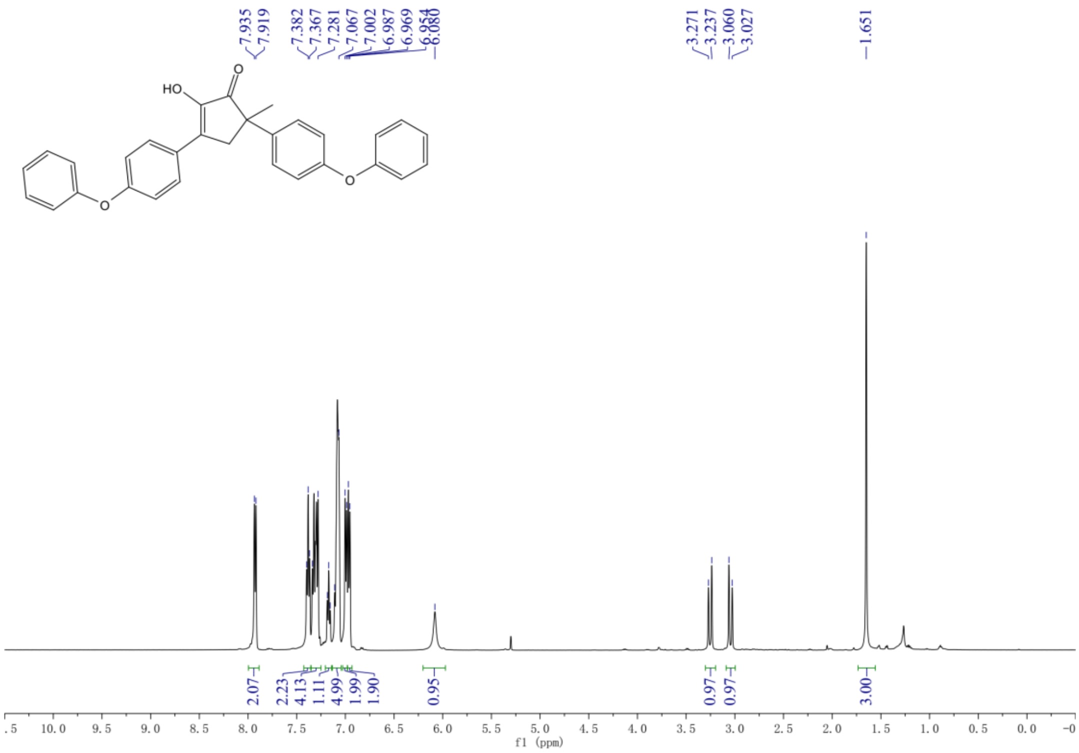

^{13}C{^{1}H} NMR (126 MHz, $CDCl_3$)

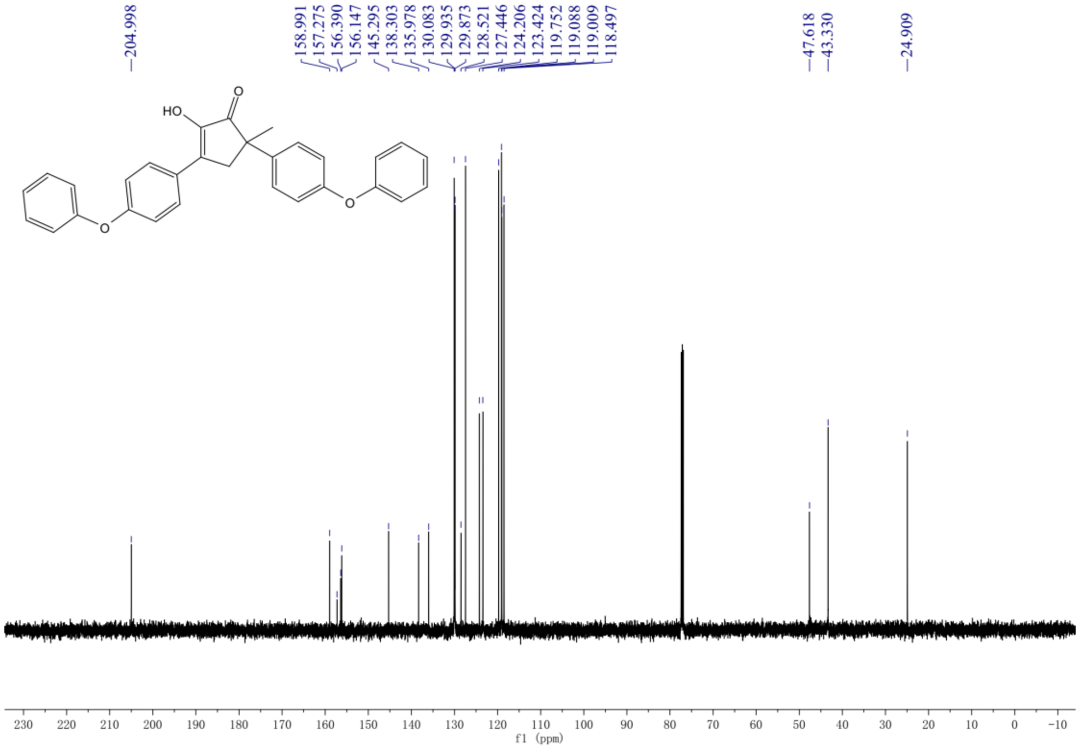

Product 8

^{1}H NMR (500 MHz, $CDCl_3$)

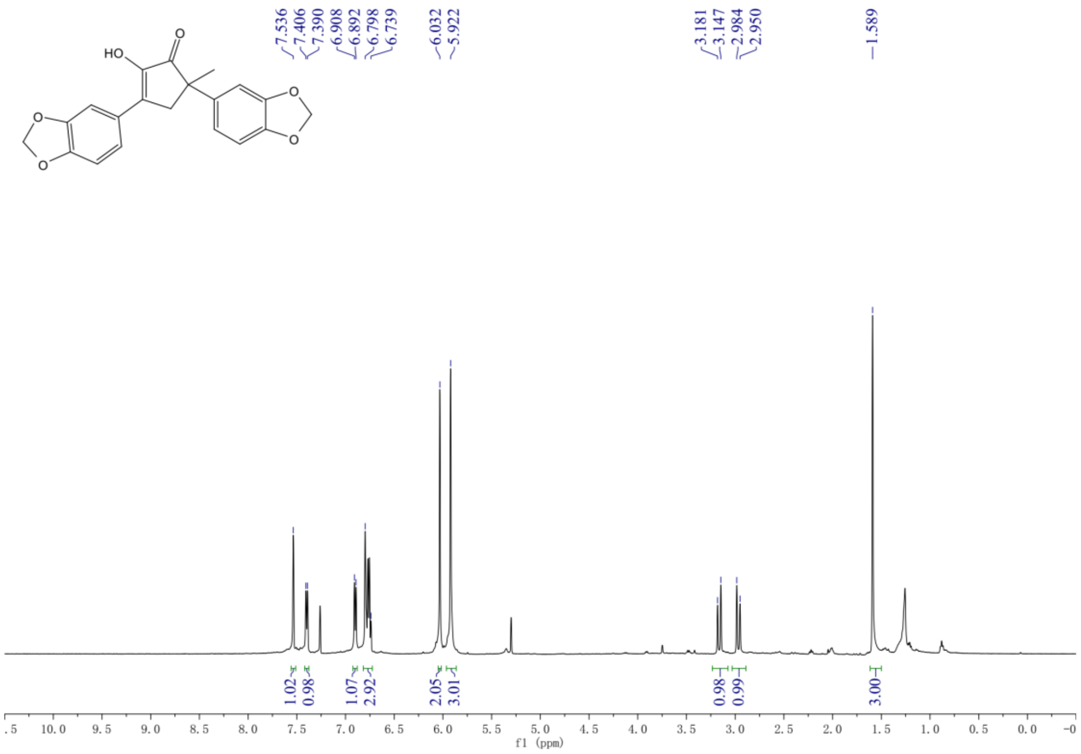

^{13}C{^{1}H} NMR (126 MHz, $CDCl_3$)

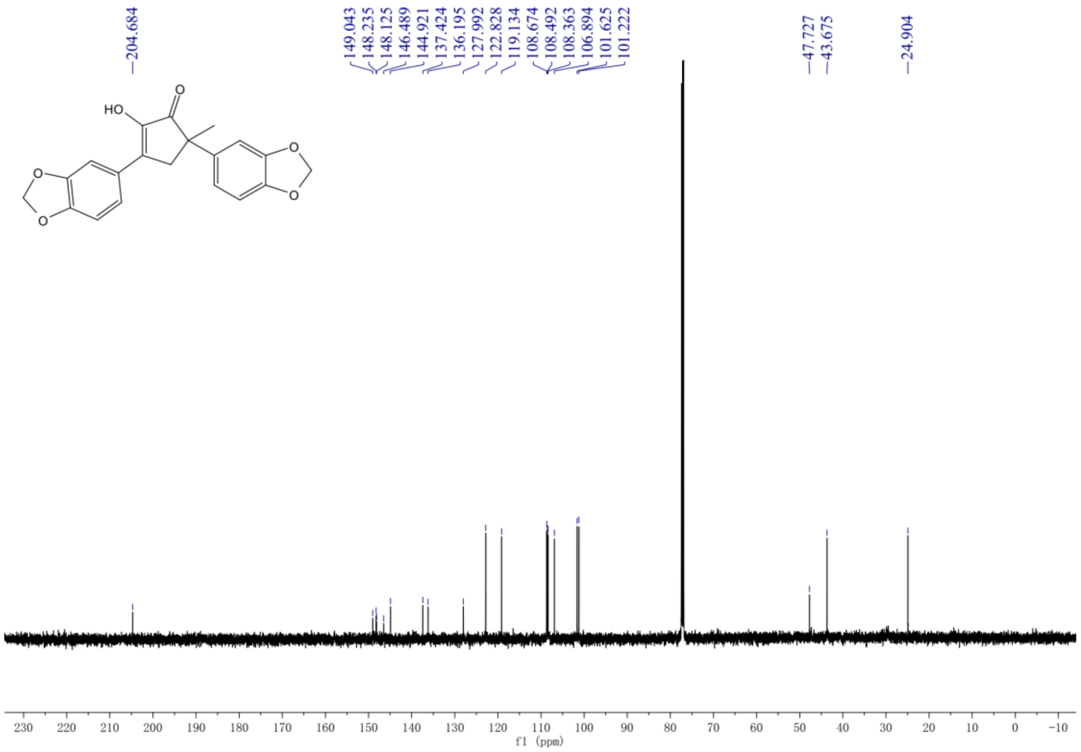

Product 9

^{1}H NMR (500 MHz, $CDCl_3$)

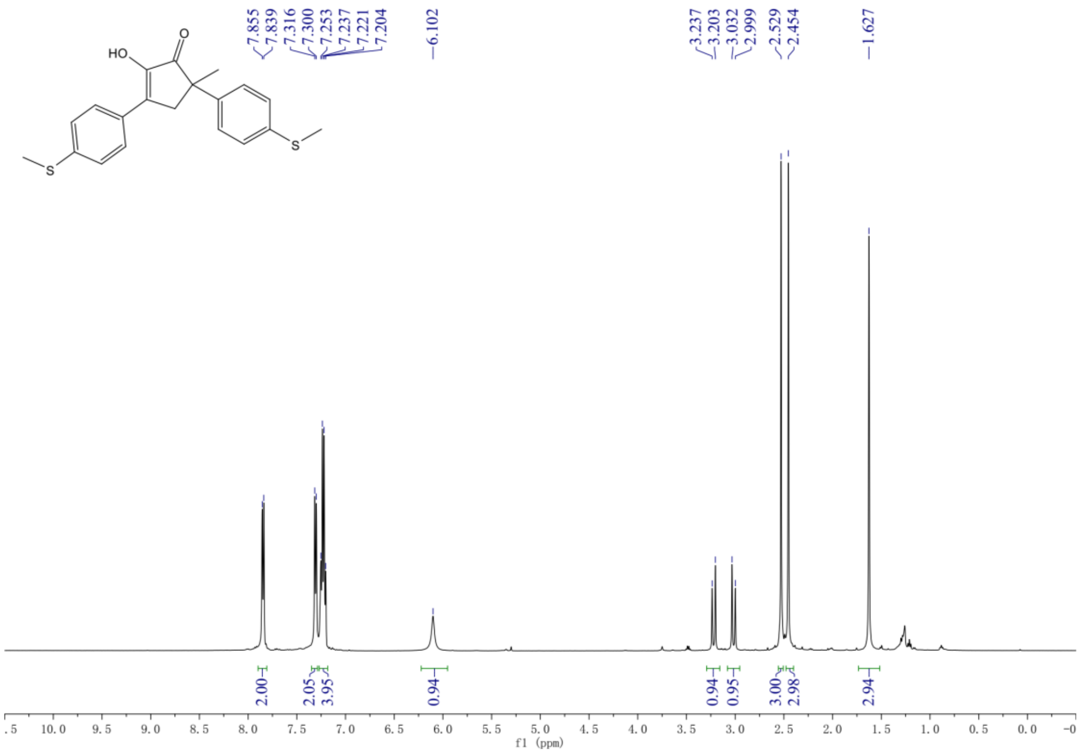

$^{13}C\{^{1}H\}$ NMR (126 MHz, $CDCl_3$)

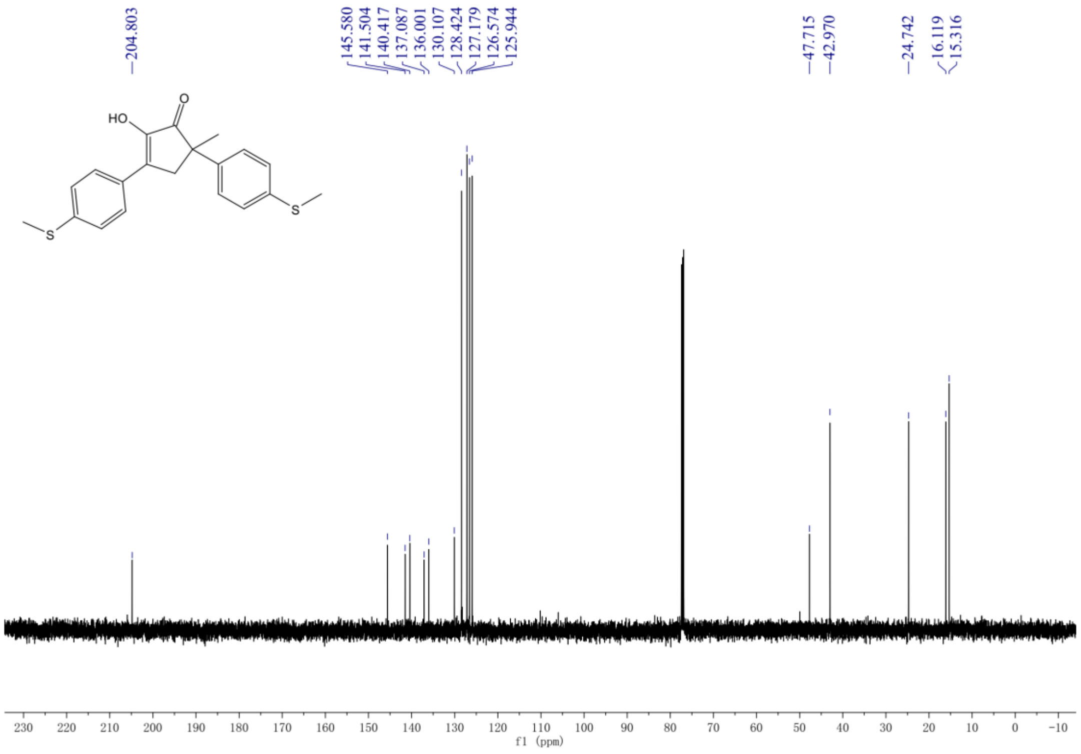

Product 10

^{1}H NMR (500 MHz, CDCl3)

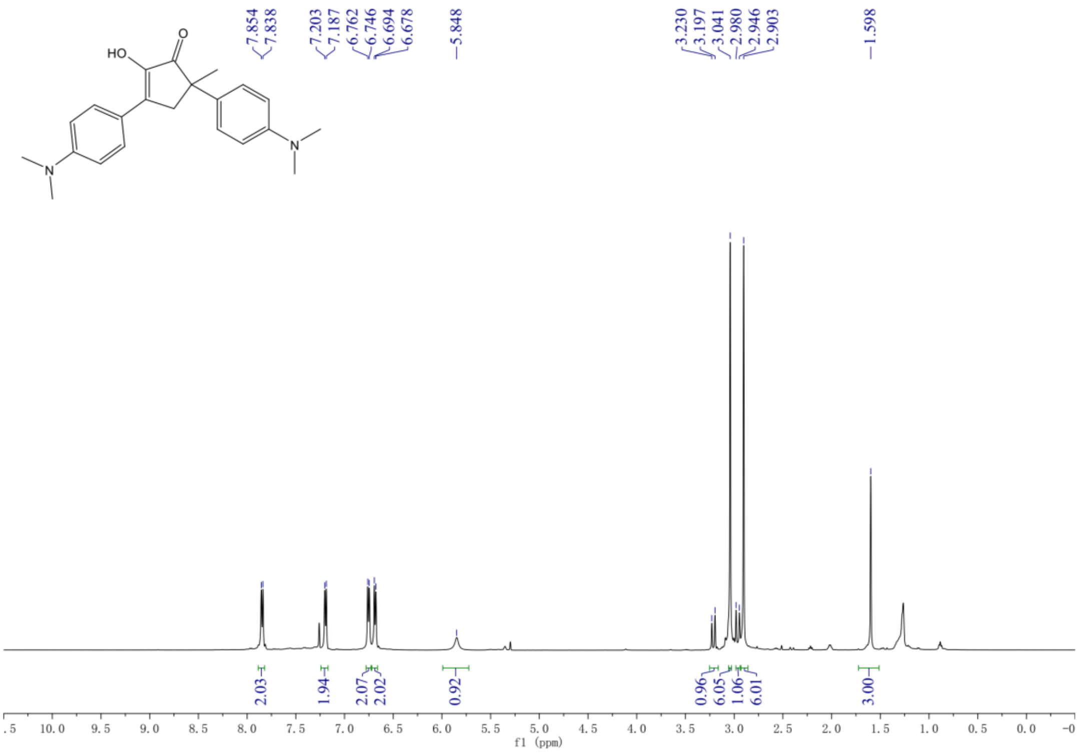

^{13}C{^{1}H} NMR (126 MHz, CDCl3)

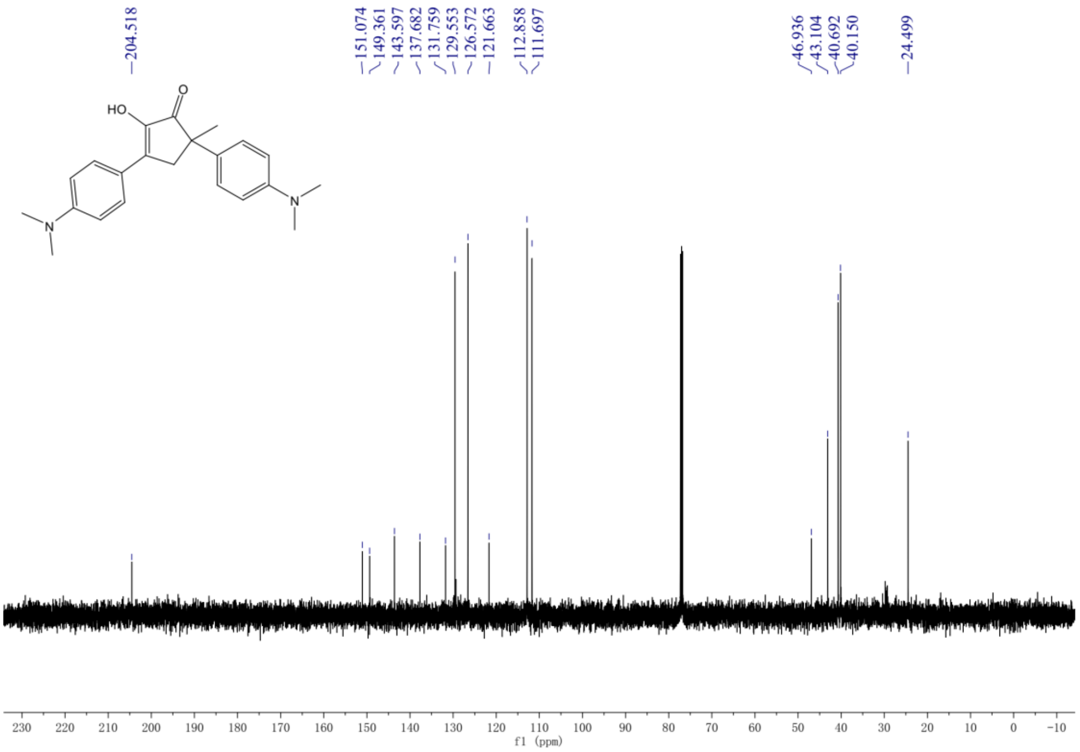

Product 11

^{1}H NMR (500 MHz, CDCl3)

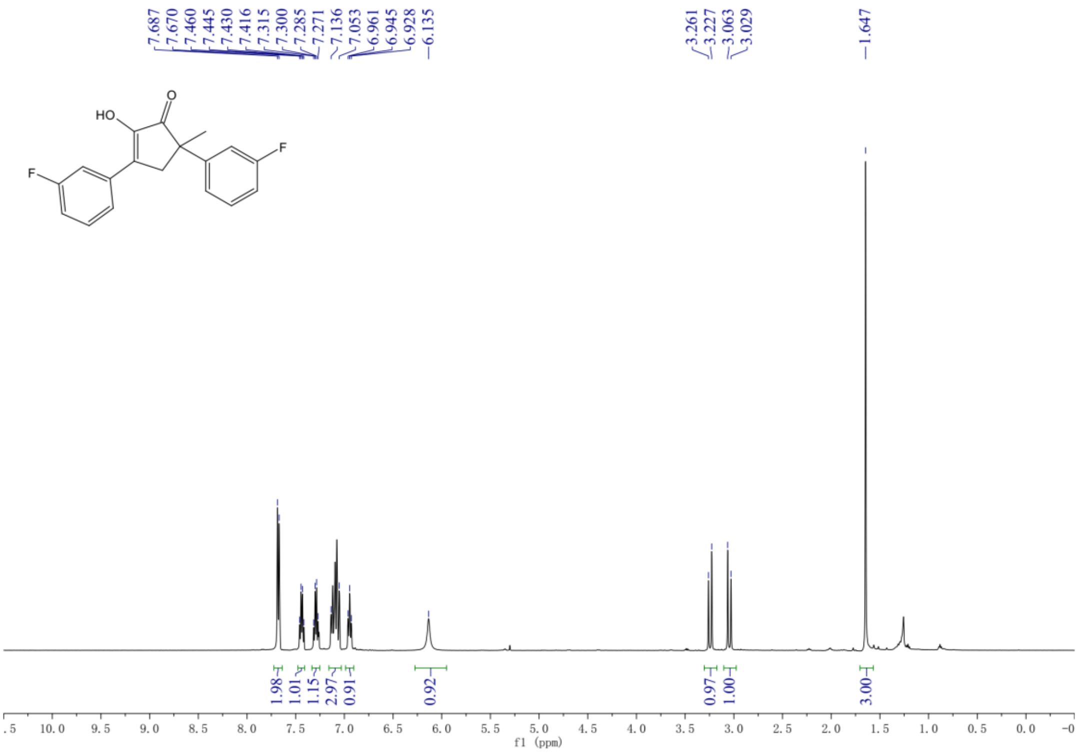

^{13}C{^{1}H} NMR (126 MHz, CDCl3)

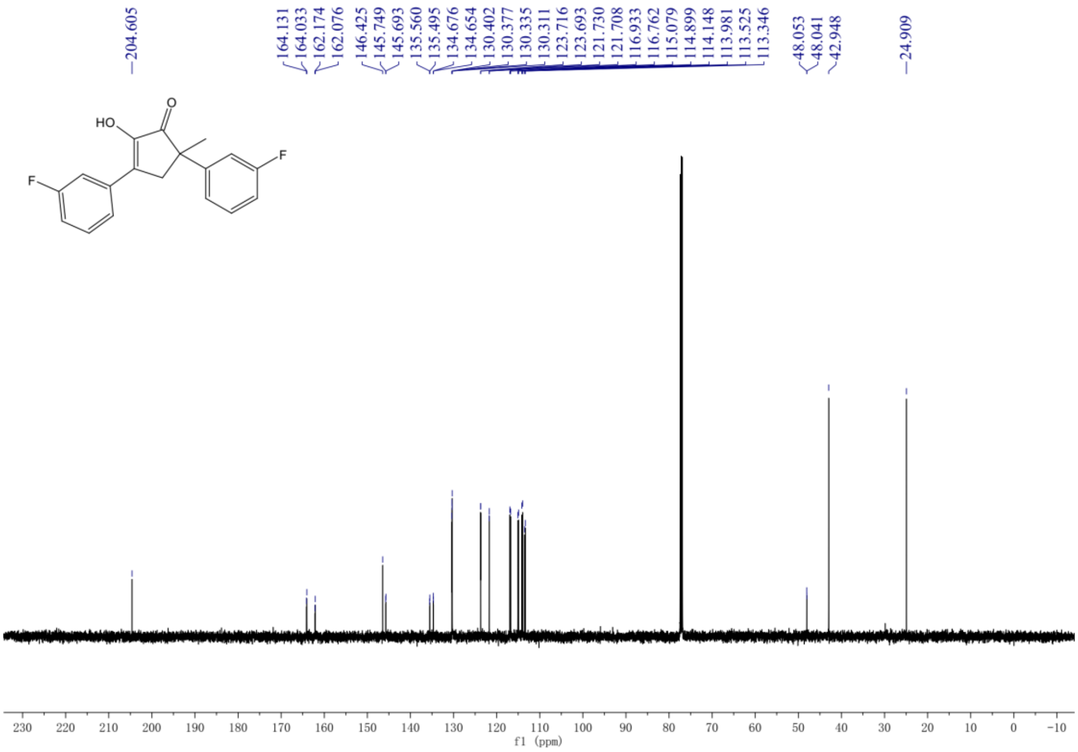

$^{19}F\{^{1}H\}$ NMR (471 MHz, $CDCl_3$)

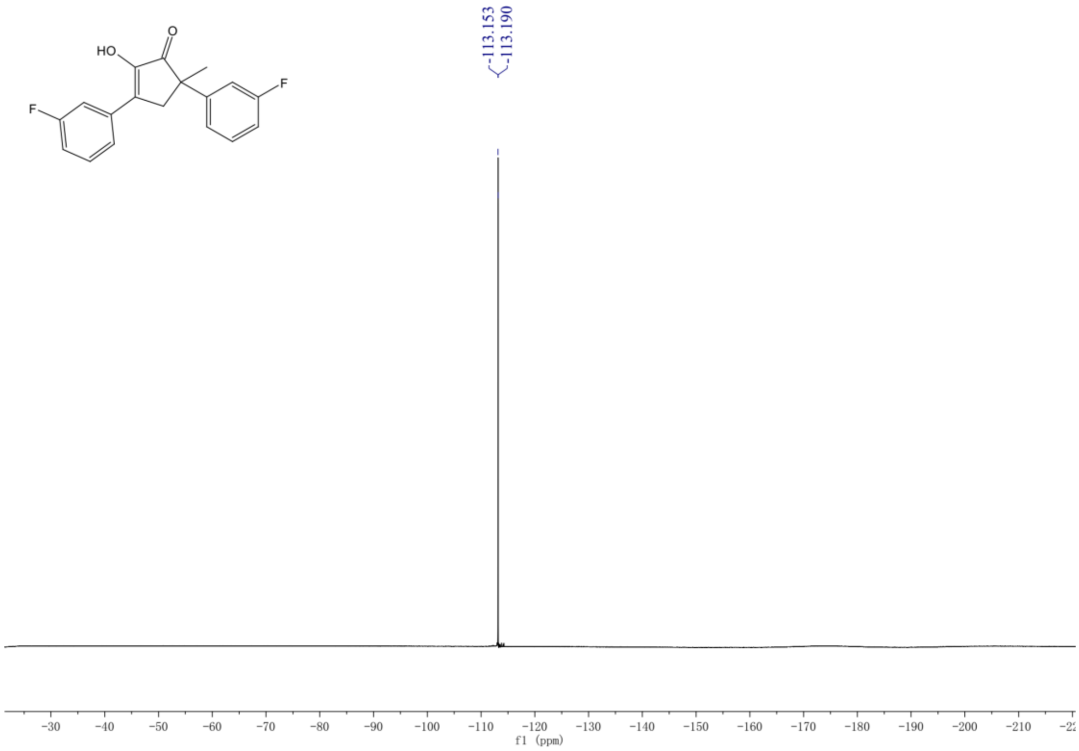

Product 12

^{1}H NMR (500 MHz, $CDCl_3$)

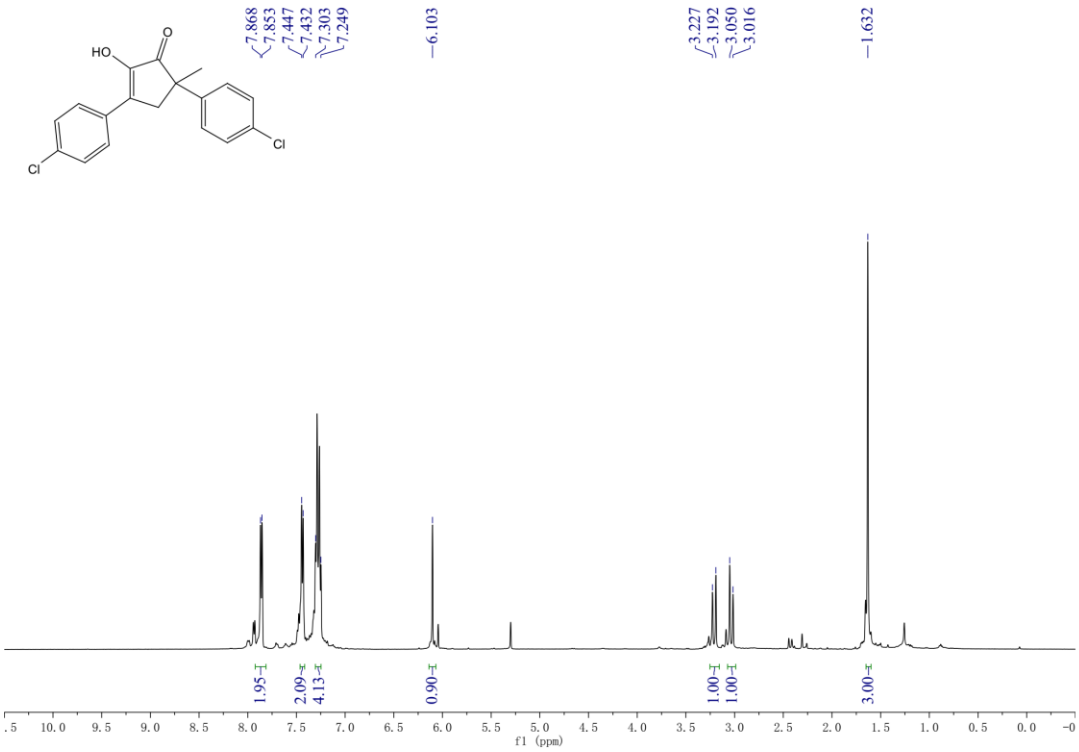

$^{13}C\{^{1}H\}$ NMR (126 MHz, $CDCl_3$)

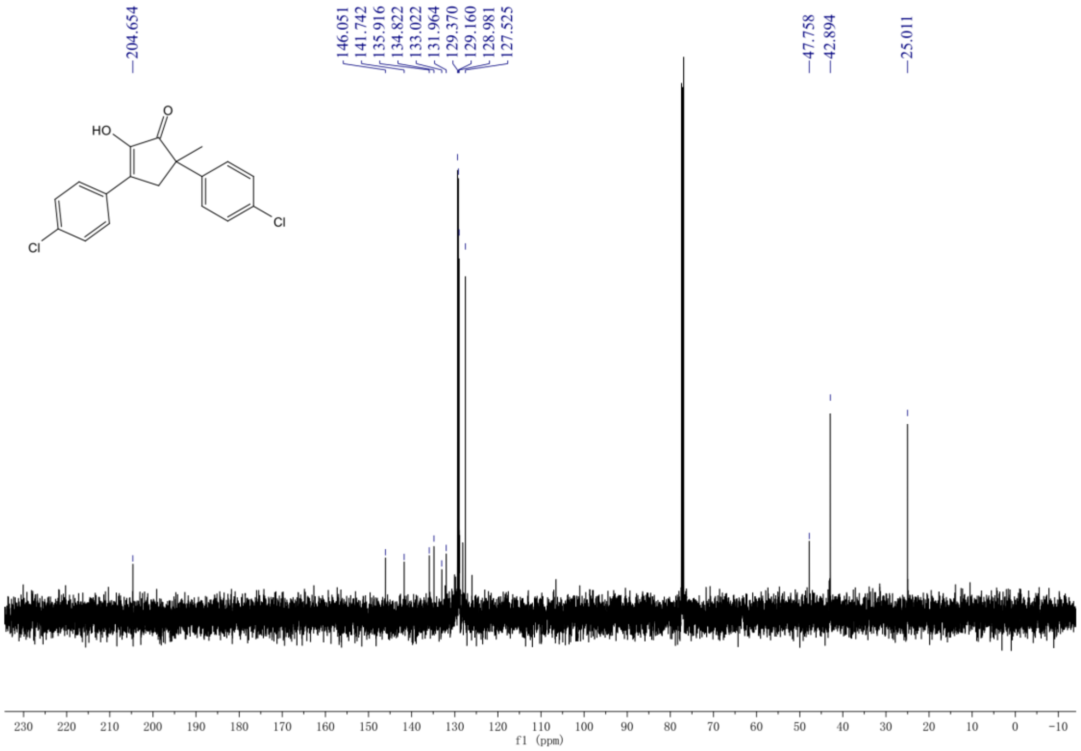

Product 13

^{1}H NMR (500 MHz, $CDCl_3$)

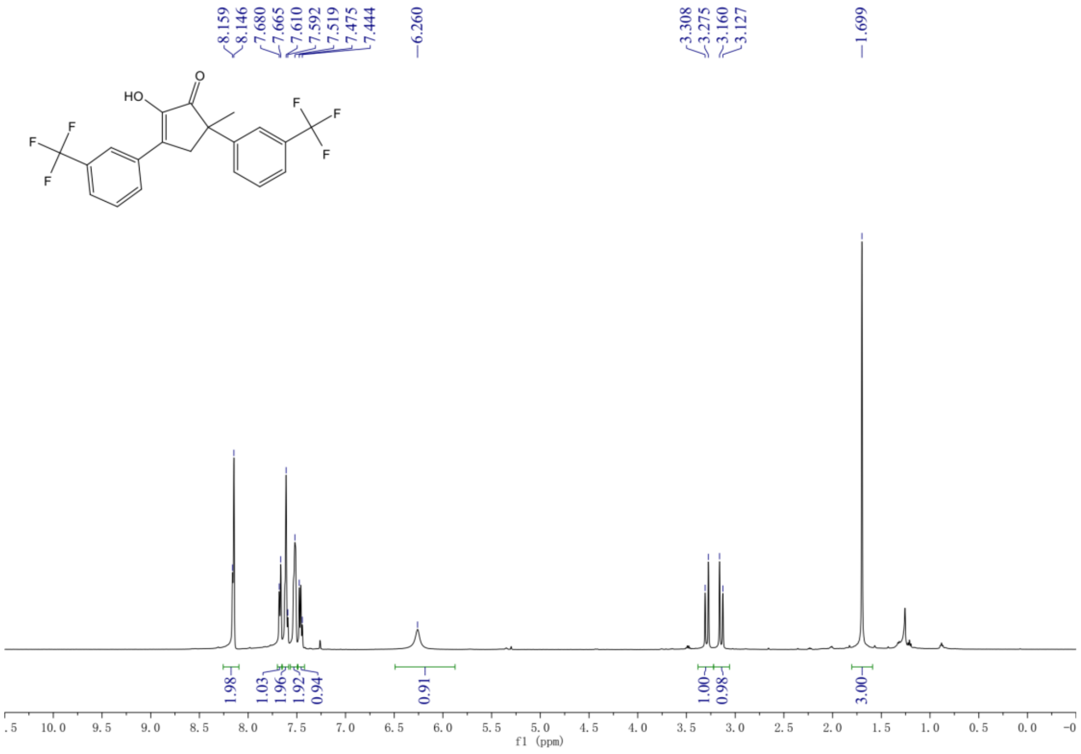

$^{13}C\{^{1}H\}$ NMR (126 MHz, CDCl$_3$)

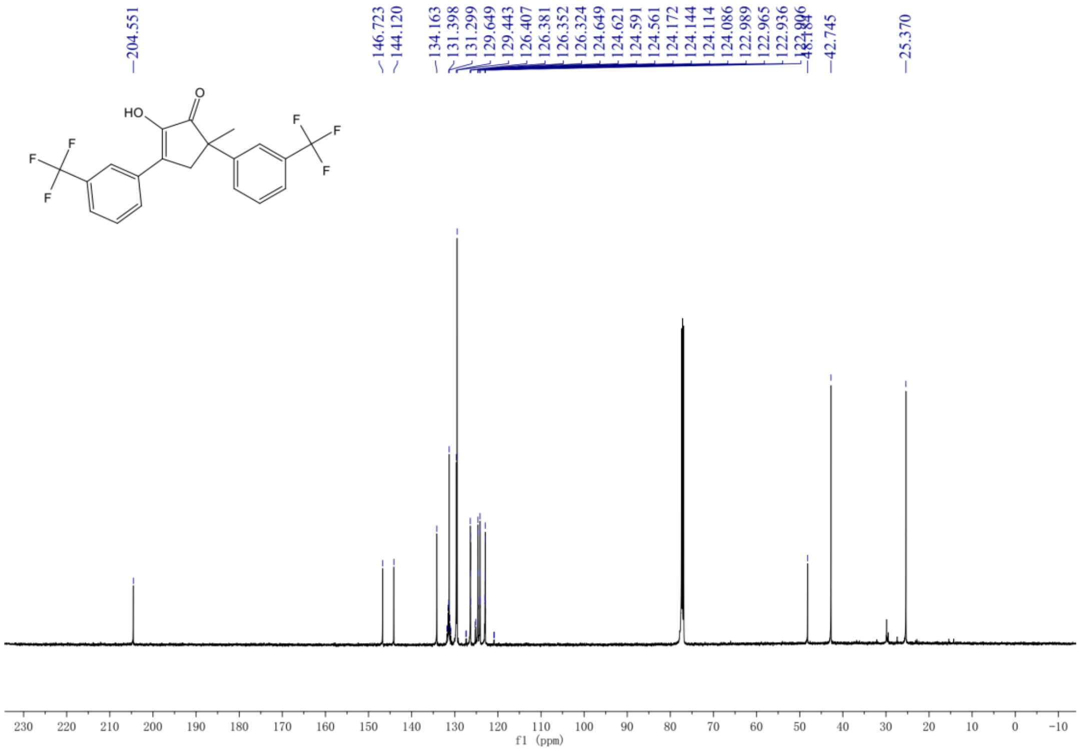

$^{19}F\{^{1}H\}$ NMR (471 MHz, CDCl$_3$)

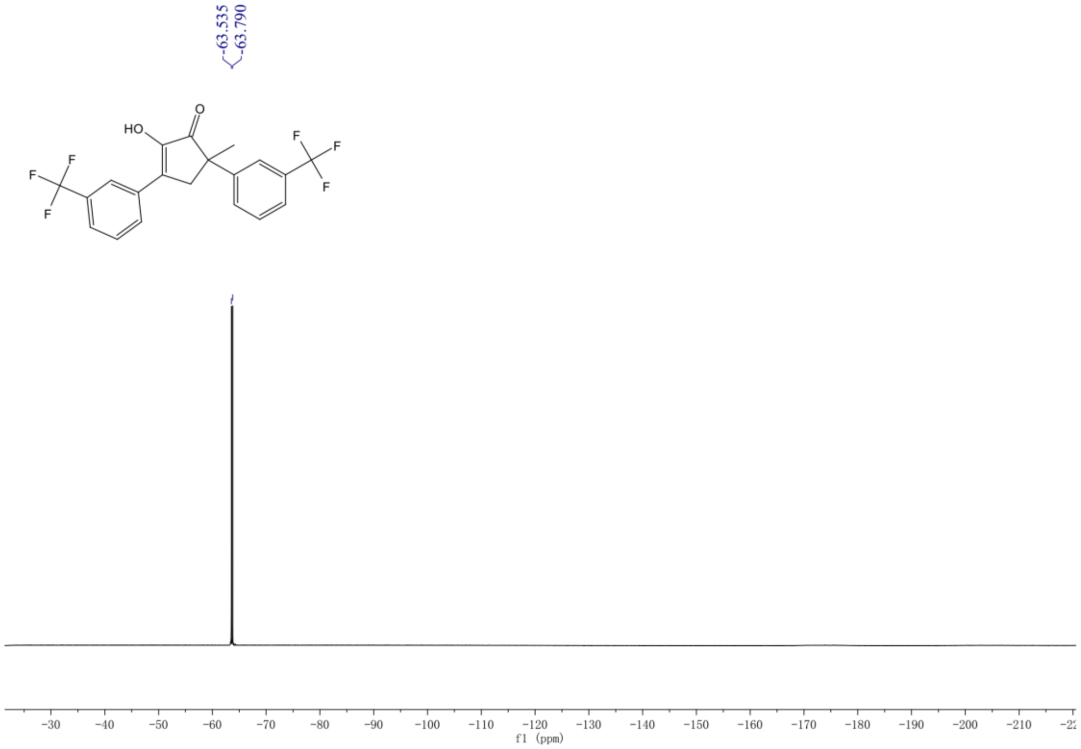

Product 14

^{1}H NMR (500 MHz, $CDCl_3$)

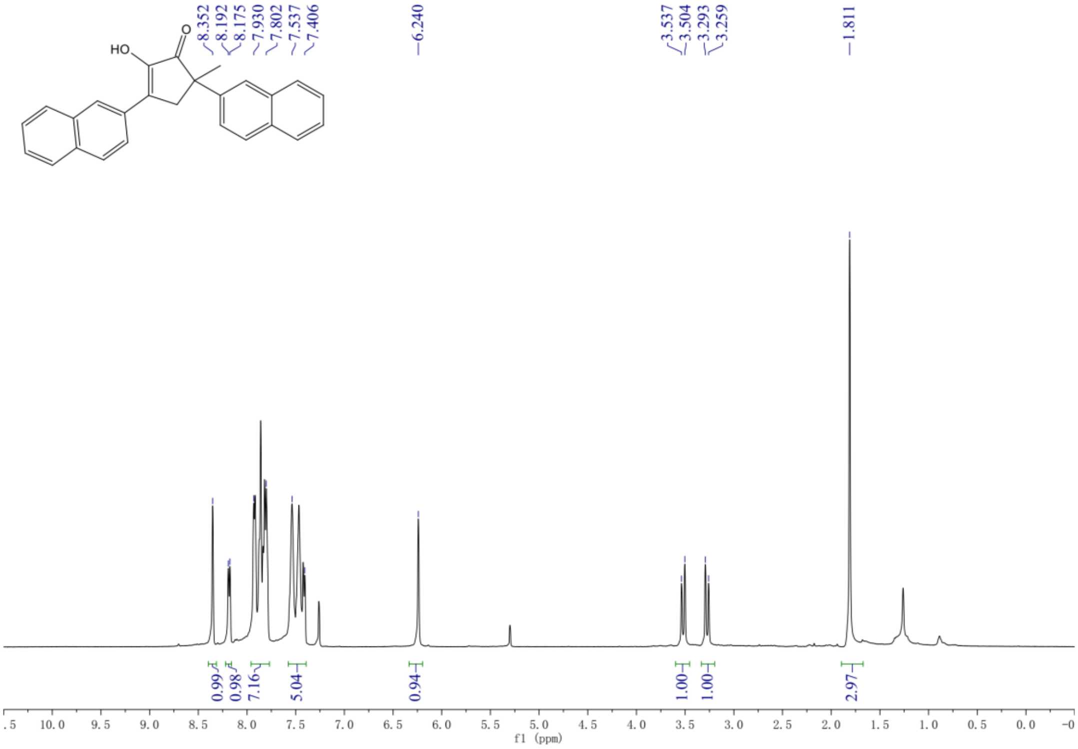

^{13}C{^{1}H} NMR (126 MHz, $CDCl_3$)

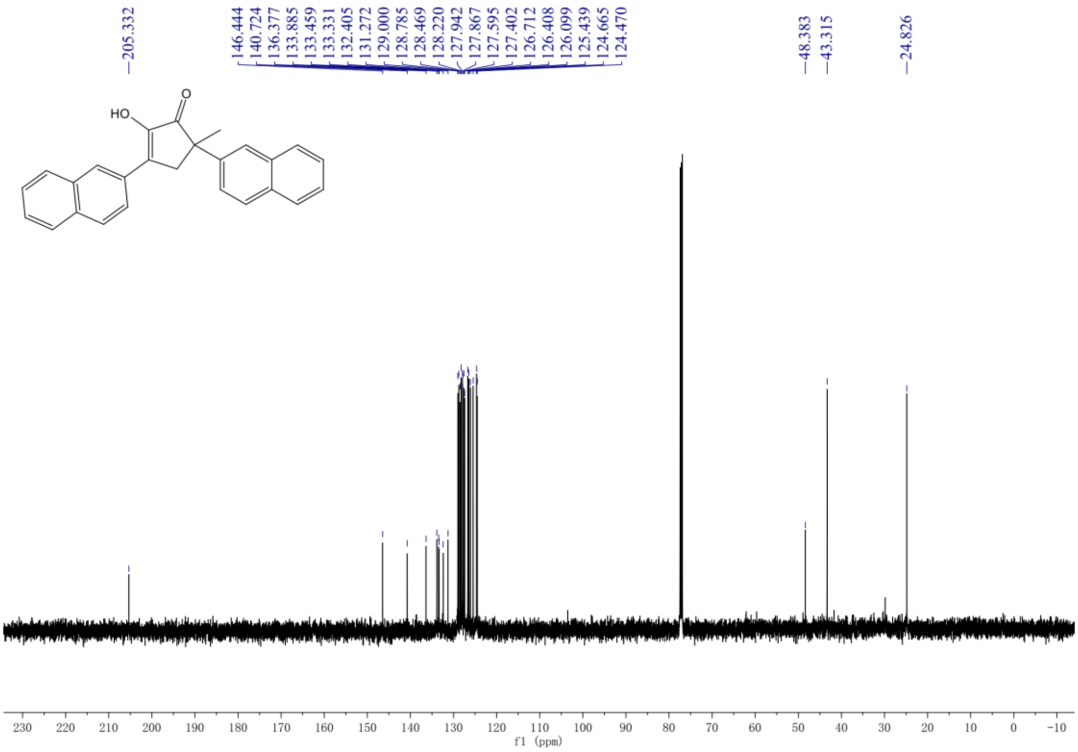

Product 15

^{1}H NMR (500 MHz, $CDCl_3$)

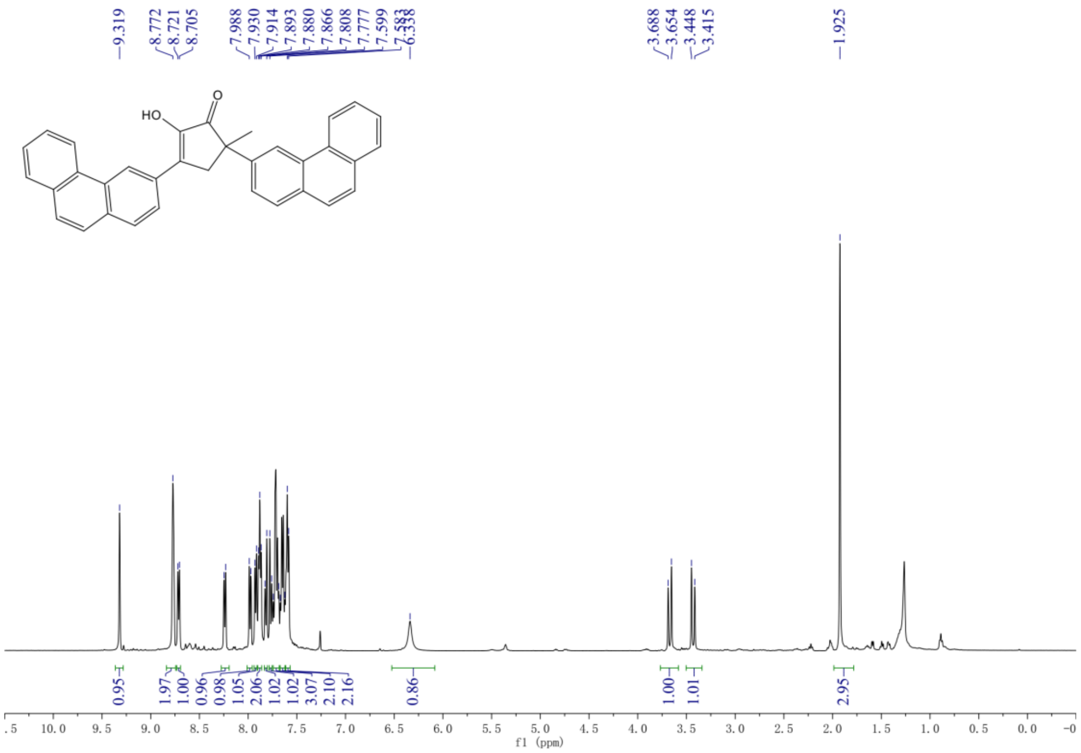

^{13}C{^{1}H} NMR (126 MHz, $CDCl_3$)

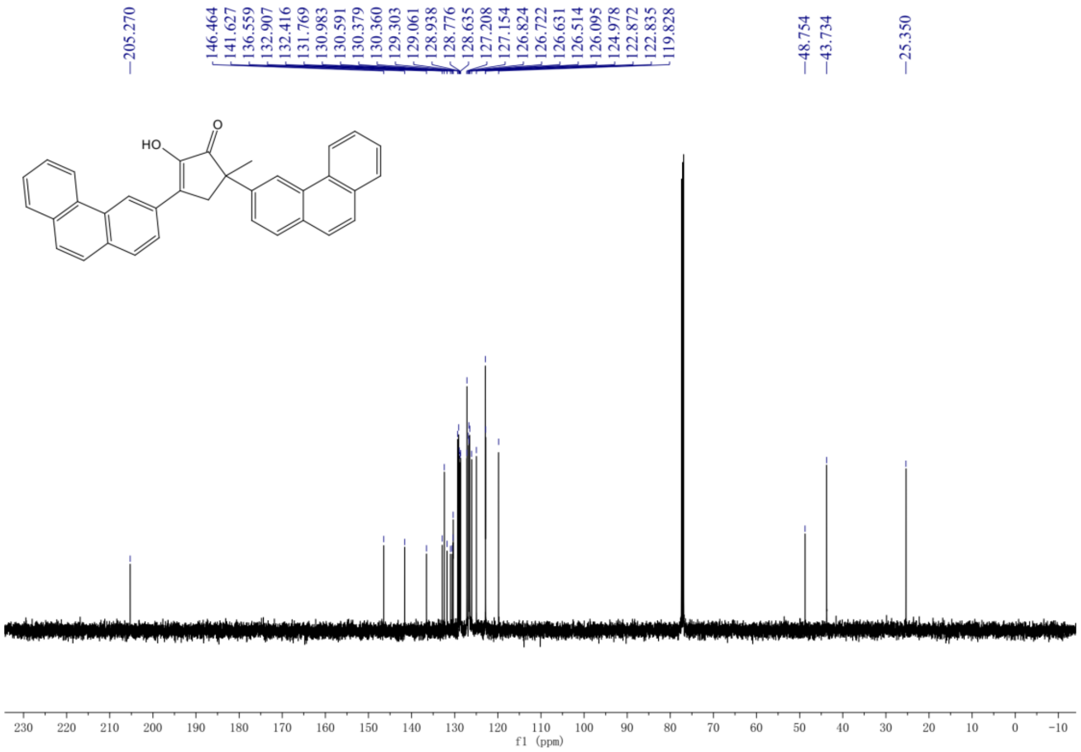

Product 16

¹H NMR (500 MHz, $CDCl_3$)

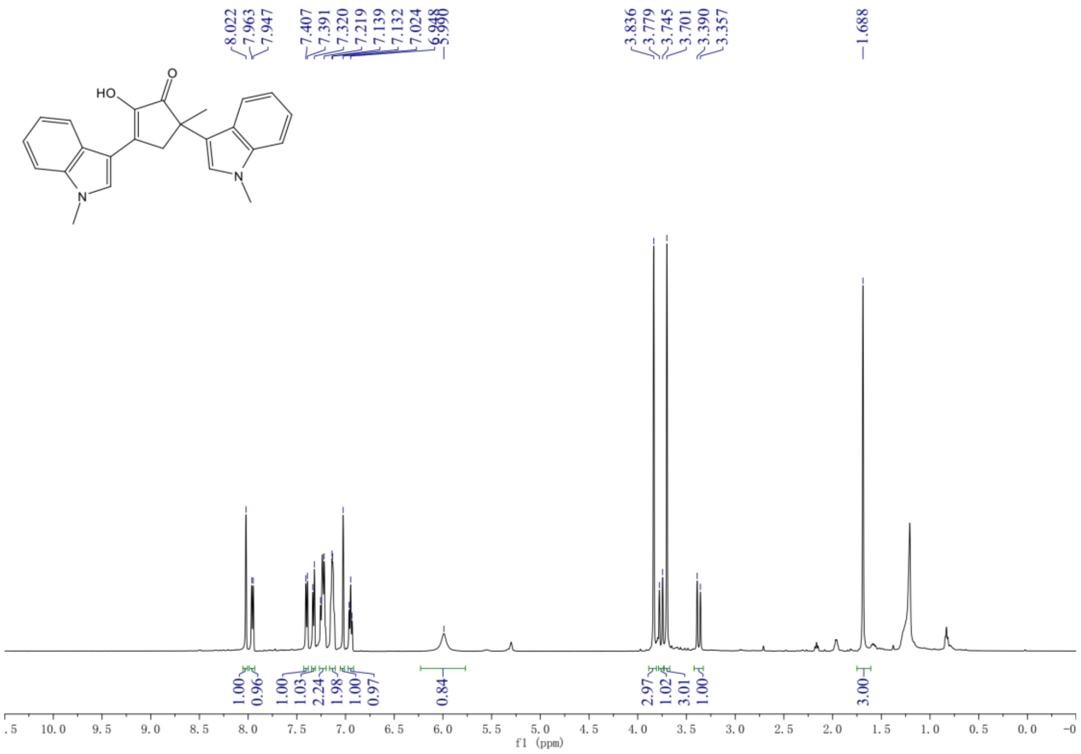

¹³C{¹H} NMR (126 MHz, $CDCl_3$)

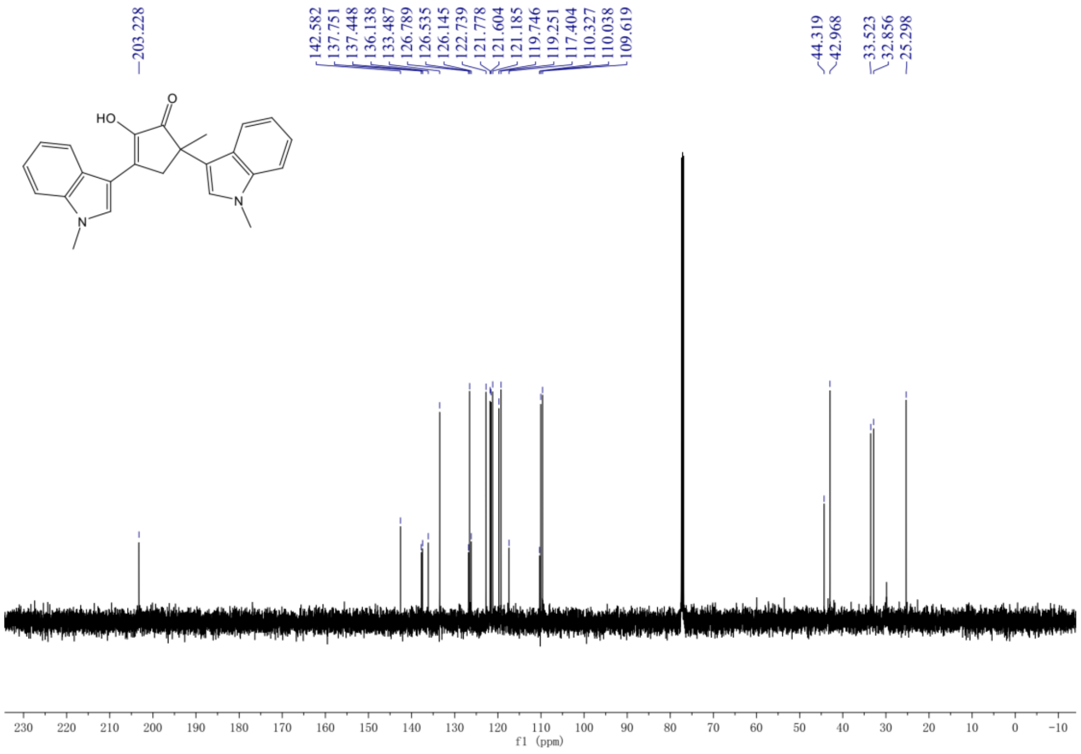

8. Computational Details

Density functional theory (DFT) calculations were performed with Gaussian 16 (C.01 revision),[10] using Truhlar's M06-L functional,[11] the triple-ξ def2-TZVP basis set,[12] W06 density fitting,[13] and Grimme's D3(0) empirical dispersion correction.[14] Frequency calculations at this level of theory were run at 403.15K to confirm stationary points and transition states, and to obtain thermodynamic corrections. Single point energies of the M06-L optimized structures were computed with ORCA (5.0.4),[15] using the range-separated meta-GGA hybrid functional ωB97M-V of the Head-Gordon group,[16] including dispersion correction,[17,18] together with the triple-ξ def2-TZVPP basis set,[12] and the corresponding auxiliary basis sets, def2/J[13] and def2-TZVPP/C,[19] for RIJCOSX density fitting. The functional and basis set selections are based on recent benchmark studies.[20] The SMD solvation model of Truhlar and coworkers was used in all calculations (optimization and single point),[21] using either toluene or ethylene glycol as implicit solvents.

Gibbs free energies at 403.15 K were computed by adding the free energy correction term from the frequency calculation to the single point energy in toluene or ethylene glycol, according to the following equation:

$$\mathbf{G^{o\omega B97M\text{-}V}_{(toluene\ or\ ethylene\ glycol,\ 403.15K)} = E^{\omega B97M\text{-}V}_{(toluene\ or\ ethylene\ glycol)} + corr^{\mathit{M06\text{-}L}}_{\mathit{freq}}(toluene\ or\ ethylene\ glycol,\ 1\ atm,\ 403.15K)}$$

where $\mathbf{E^{\omega B97M\text{-}V}_{(toluene\ or\ ethylene\ glycol)}}$ is the single point energy, and $\mathbf{corr^{\mathit{M06\text{-}L}}_{\mathit{freq}}}$ is the thermal correction to the Gibbs free energy from the frequency calculation (at T = 403.15 K and P = 1 atm).

Free energy values (G^o) were corrected to account for changes in standard states ($G^o \rightarrow G$). Standard state corrections[22] were employed, such that all species are treated as 1 M (using an ideal gas approximation).[23-25] The obtained energy data based on standard state corrections are provided in Tables S2 and S3, respectively.

Table S2. Energy data for calculated complexes, transition states, substrates and products based on standard state corrections, using toluene as an implicit solvent.

**Structure**	$\mathbf{E^{\omega B97M\text{-}V}_{Toluene}}$	$\mathbf{G^{o\omega B97M\text{-}V}_{(Toluene,\ 403.15K)}}$	**Imaginary frequency**	**G T = 403.15K**
Energy Unit	Hartree	Hartree	cm^{-1}	kcal
**Mn-5**	-3598.64348	-3598.162445	-	-2257844.372
**Mn-6**	-3542.046624	-3541.603951	-	-2222353.916
**Mn-6-^{i}Pr**	-3089.50692	-3089.035305	-	-1938367.091
**Mn-7**	-3543.214687	-3542.752353	-	-2223074.539

**$TS_{6,7}$-PE**	-3928.114597	-3927.524977	-435.2828	-2464519.361
**$TS_{6,7}$-PE-^{i}Pr**	-3475.567347	-3474.949988	-577.9326	-2180528.555
**$TS_{6,7}$-EG**	-3772.294197	-3771.773811	-354.8106	-2366785.504
**$TS_{6,7}$-IV**	-4464.465275	-4463.742767	-410.4265	-2800996.024
**$TS_{6,7}$-VI**	-4465.673663	-4464.928701	-427.4796	-2801740.197
**$TS_{7,6}$**	-3543.2066	-3542.749832	-1239.7294	-2223072.957
**$TS_{7,6}$-IV**	-4465.68639	-4464.94134	-330.5663	-2801748.129
**$TS_{7,6}$-V**	-4464.476919	-4463.755308	-256.4596	-2801003.893
**H_2**	-1.161064506	-1.168069506	-	-730.4010309
**NH_3**	-56.55398578	-56.54693178	-	-35480.63711
**H_2O**	-76.43820352	-76.44324552	-	-47965.57398
**PE**	-386.0938832	-385.9817092	-	-242200.9599
**Intermediate I**	-384.9022177	-384.8124187	-	-241467.2302
**EG**	-230.2671517	-230.2224197	-	-144462.0058
**Intermediate III**	-229.0648456	-229.0442076	-	-143722.6777
**Intermediate IX**	-769.8128627	-769.5994397	-	-482921.0858
**Intermediate II**	-693.3517492	-693.1626602		-434957.0067
**Intermediate IV**	-922.4573685	-922.2122525	-	-578685.6259
**Intermediate VII**	-922.465723	-922.220872	-	-578691.0346
**Intermediate VIII**	-922.4633286	-922.2181906	-	-578689.352
**Product 1**	-846.0351896	-845.8107626	-	-530743.6909
**Intermediate V**	-921.2454832	-921.0244922	-	-577940.3063
**Intermediate VI**	-923.652424	-923.384887	-	-579421.454

Cartesian coordinates for calculated structures

Mn-5

Mn	11.3214780000	13.3052860000	6.5039470000
P	11.4189950000	13.8148240000	8.7032900000
P	11.7815140000	12.5508870000	4.4169340000
O	8.7636370000	14.6434040000	5.9255720000
O	9.9861060000	10.7668500000	7.1201100000
N	13.1788840000	12.3854480000	6.9447800000
N	12.5363830000	15.0540780000	6.0724040000
H	12.5089010000	15.3651950000	5.1068170000
H	13.4989700000	14.7991000000	6.2739980000
H	12.3223450000	15.8644300000	6.6446210000
C	12.7306320000	12.7285650000	9.2900140000

C	13.0124660000	12.4711310000	10.6284820000
H	12.3706750000	12.8857460000	11.3965090000
C	14.0896910000	11.6816310000	10.9836720000
H	14.2949980000	11.4630510000	12.0229150000
C	14.9155100000	11.1859590000	9.9786940000
H	15.7825750000	10.5893820000	10.2430030000
C	14.6760290000	11.4398820000	8.6415090000
C	13.5348590000	12.1836140000	8.2609170000
C	15.6337510000	11.0047990000	7.5769950000
H	16.1395290000	10.0793670000	7.8608630000
H	16.4372490000	11.7523260000	7.4963140000
C	14.9688410000	10.8627780000	6.2439680000
C	15.4923970000	10.0478380000	5.2597240000
H	16.3737050000	9.4594950000	5.4937610000
C	14.9304590000	9.9664620000	3.9871700000
H	15.3593250000	9.3168260000	3.2362010000
C	13.8164520000	10.7317700000	3.7066820000
H	13.3541530000	10.6831620000	2.7265930000
C	13.2544250000	11.5571500000	4.6807100000
C	13.8028020000	11.6234340000	5.9830470000
C	12.0016450000	15.5069570000	9.0855630000
C	11.1102460000	16.5703860000	8.9086540000
H	10.0761950000	16.3652280000	8.6497420000
C	11.5332440000	17.8800790000	9.0612130000
H	10.8286420000	18.6912550000	8.9275300000
C	12.8569420000	18.1517090000	9.3834860000
H	13.1879780000	19.1754460000	9.5020730000
C	13.7507380000	17.1062090000	9.5531790000
H	14.7832430000	17.3105680000	9.8074940000
C	13.3282050000	15.7918610000	9.4032610000
H	14.0337440000	14.9802660000	9.5390520000
C	9.9947900000	13.6744590000	9.8344720000
C	8.8121870000	13.0703160000	9.4133860000
H	8.7292410000	12.6958970000	8.4023970000
C	7.7304920000	12.9576020000	10.2740820000
H	6.8190300000	12.4873990000	9.9280190000
C	7.8137930000	13.4511420000	11.5659330000
H	6.9687170000	13.3637440000	12.2369880000
C	8.9797090000	14.0710020000	11.9930730000
H	9.0472450000	14.4713200000	12.9967380000
C	10.0585270000	14.1878080000	11.1332180000
H	10.9550130000	14.6966230000	11.4680120000

C	10.6122680000	11.4558070000	3.5418690000
C	9.7591600000	11.9091750000	2.5373860000
H	9.8193580000	12.9327750000	2.1910720000
C	8.8336970000	11.0538500000	1.9586140000
H	8.1825250000	11.4224750000	1.1760970000
C	8.7452440000	9.7348660000	2.3745420000
H	8.0233570000	9.0680410000	1.9205890000
C	9.5890260000	9.2731760000	3.3741810000
H	9.5296120000	8.2443300000	3.7056630000
C	10.5132570000	10.1252580000	3.9545670000
H	11.1696450000	9.7543550000	4.7323660000
C	12.2032290000	13.8258090000	3.1721010000
C	13.4210780000	13.8579960000	2.4949210000
H	14.1504480000	13.0754010000	2.6607760000
C	13.7128400000	14.8907330000	1.6147830000
H	14.6641400000	14.8987060000	1.0977770000
C	12.7957960000	15.9068970000	1.3974400000
H	13.0267850000	16.7108550000	0.7104170000
C	11.5837720000	15.8942750000	2.0733020000
H	10.8646340000	16.6886590000	1.9186680000
C	11.2947780000	14.8693740000	2.9596960000
H	10.3572140000	14.8807630000	3.5049150000
C	9.7846290000	14.1278440000	6.1435400000
C	10.4964810000	11.7803020000	6.8732180000

Mn-6

Mn	12.3138530000	8.9805010000	11.1483070000
P	10.9790850000	10.5550020000	10.2956470000
P	12.7489170000	6.9157250000	11.8870390000
O	13.0759130000	10.4400370000	13.5886520000
C	10.5413000000	9.9116130000	8.6806390000
C	9.9001230000	10.6230040000	7.6760450000
H	9.6608310000	11.6686970000	7.8309350000
C	9.5785020000	10.0019610000	6.4822710000
H	9.0868250000	10.5492710000	5.6894400000
C	9.9003670000	8.6610650000	6.3210650000
C	12.0268880000	4.5276560000	8.1053930000
H	11.8072340000	3.9889740000	7.1892300000
C	12.7170680000	3.8862780000	9.1248800000
H	13.0395450000	2.8601660000	9.0107980000

C	12.9810550000	4.5855550000	10.2899580000
H	13.5089250000	4.1088520000	11.1080130000
C	12.5736860000	5.9059010000	10.4164120000
C	14.1789640000	6.3331290000	12.8318590000
C	11.3002000000	6.5060070000	12.9220580000
C	10.2628160000	5.6898120000	12.4738130000
H	10.3336420000	5.2121070000	11.5040670000
C	9.1422140000	5.4824790000	13.2651320000
H	8.3454180000	4.8431300000	12.9063550000
C	9.0419520000	6.0879250000	14.5077150000
H	8.1655130000	5.9256050000	15.1221650000
C	10.0670810000	6.9074240000	14.9609480000
H	9.9935840000	7.3870410000	15.9286980000
C	11.1845320000	7.1206530000	14.1730290000
H	11.9806380000	7.7640650000	14.5312180000
C	12.7550830000	9.8557470000	12.6360990000
H	9.6526690000	8.1613840000	5.3900780000
O	14.8703090000	9.6884380000	9.9480490000
C	10.5416140000	7.9252260000	7.3058840000
C	10.8946910000	8.5519390000	8.5173210000
C	10.8614230000	6.4811260000	7.0717140000
H	9.9354000000	5.9242530000	6.8835130000
H	11.4337390000	6.3746390000	6.1434960000
C	11.6089000000	5.8465120000	8.2033530000
C	11.8995010000	6.5781810000	9.3712140000
C	13.8540000000	9.4109220000	10.4404440000
C	15.3802160000	7.0342200000	12.7400970000
H	15.4321410000	7.9285430000	12.1310490000
C	16.5000530000	6.5970350000	13.4292240000
H	17.4275540000	7.1496000000	13.3525240000
C	16.4283650000	5.4610540000	14.2212540000
H	17.3021660000	5.1232160000	14.7637970000
C	15.2342160000	4.7618490000	14.3260640000
H	15.1744900000	3.8783850000	14.9488510000
C	14.1133590000	5.1947340000	13.6366800000
H	13.1790800000	4.6523390000	13.7272860000
C	9.5025030000	10.3252290000	11.3443920000
C	9.4141560000	11.0061480000	12.5625230000
H	10.1653220000	11.7436620000	12.8227270000
C	8.3737330000	10.7471850000	13.4379180000
H	8.3141630000	11.2877700000	14.3739870000
C	7.4170410000	9.7922300000	13.1208300000

H	6.6083740000	9.5847950000	13.8100260000
C	7.5022730000	9.1039630000	11.9206370000
H	6.7600390000	8.3572240000	11.6681970000
C	8.5373490000	9.3665280000	11.0362610000
H	8.5955830000	8.8256000000	10.0991470000
C	11.2171770000	12.3432570000	10.1754130000
C	12.5151400000	12.8498410000	10.1366910000
H	13.3563910000	12.1711260000	10.2097390000
C	12.7303500000	14.2132500000	10.0122820000
H	13.7420360000	14.5967790000	9.9843110000
C	11.6526050000	15.0820410000	9.9332560000
H	11.8217030000	16.1473540000	9.8411110000
C	10.3566620000	14.5875020000	9.9804030000
H	9.5139370000	15.2647330000	9.9249400000
C	10.1375830000	13.2253310000	10.1021610000
H	9.1245420000	12.8424820000	10.1483640000
N	11.5559930000	7.9101900000	9.5543390000

Mn-6-^{i}Pr

Mn	12.0768450000	8.9228210000	11.3245120000
P	10.8549180000	10.5703390000	10.3902540000
P	12.7651310000	6.8845360000	12.0080000000
O	12.6057310000	10.3049440000	13.8729600000
C	10.4843520000	9.8729440000	8.7676410000
C	9.9198830000	10.5674790000	7.7047450000
H	9.7119390000	11.6246570000	7.8036910000
C	9.6271050000	9.9271650000	6.5127440000
H	9.2051100000	10.4747410000	5.6807470000
C	9.8738550000	8.5669130000	6.4139780000
C	12.1201660000	4.5616240000	8.1526440000
H	11.9035320000	4.0311040000	7.2313190000
C	12.9518020000	3.9821150000	9.0989300000
H	13.3987290000	3.0129790000	8.9224270000
C	13.1848670000	4.6644510000	10.2815020000
H	13.8155910000	4.2199990000	11.0428960000
C	12.6315050000	5.9206540000	10.4943840000
C	12.3844760000	9.7573450000	12.8689550000
H	9.6330900000	8.0415070000	5.4957150000
O	14.5617880000	9.9097250000	10.1739580000
C	10.4160830000	7.8393960000	7.4620370000

C	10.7703680000	8.4923920000	8.6597550000
C	10.5836410000	6.3595550000	7.3395000000
H	9.6061980000	5.8738110000	7.4721380000
H	10.8891040000	6.0911020000	6.3249670000
C	11.5440230000	5.8087420000	8.3415550000
C	11.8345080000	6.5427200000	9.5078820000
C	13.5870870000	9.5102240000	10.6688640000
N	11.3891960000	7.8456280000	9.7292480000
C	14.4084020000	6.4712980000	12.7368310000
H	14.4374890000	5.3792600000	12.8144160000
C	15.5243110000	6.9294610000	11.8102570000
H	16.4821940000	6.5372680000	12.1544790000
H	15.6022010000	8.0160700000	11.8028710000
C	14.5794900000	7.0639100000	14.1264950000
H	14.4660570000	8.1486210000	14.1116960000
H	15.5799090000	6.8475780000	14.5041480000
H	13.8684900000	6.6627860000	14.8469060000
C	11.4731720000	6.0810800000	13.0958590000
H	10.6423640000	5.9651140000	12.3904590000
C	11.8648510000	4.7010140000	13.5950810000
H	12.1861430000	4.0443720000	12.7869060000
H	11.0147700000	4.2215200000	14.0834260000
H	12.6687510000	4.7502220000	14.3308470000
C	10.9966770000	6.9817690000	14.2266690000
H	11.7894730000	7.2165530000	14.9366300000
H	10.1974960000	6.4912360000	14.7852710000
H	10.6057530000	7.9303580000	13.8576210000
H	15.3798710000	6.5995290000	10.7815880000
C	10.4704790000	13.3610880000	9.8815540000
H	9.7698140000	13.1175500000	9.0864150000
H	9.8880320000	13.5626910000	10.7809500000
H	10.9598440000	14.2963510000	9.6056480000
C	11.5214800000	12.2863100000	10.1259730000
H	12.1205170000	12.1678160000	9.2169250000
H	13.2752710000	12.0162490000	11.4015170000
H	12.8822250000	13.6892020000	11.0286690000
C	12.4527730000	12.7116890000	11.2537410000
H	11.9256860000	12.8022670000	12.2038640000
C	9.1734340000	10.7728700000	11.1607160000
C	9.2621210000	11.3586600000	12.5610040000
H	9.7782860000	12.3170820000	12.5928150000
H	9.7878780000	10.6832240000	13.2376800000

H	8.2620760000	11.5140610000	12.9691990000
H	8.6163220000	11.4585610000	10.5135470000
H	9.0027920000	8.6971470000	11.7795080000
H	8.3207870000	9.0135740000	10.1869960000
H	7.4670540000	9.5383850000	11.6351110000
C	8.4544520000	9.4322340000	11.1834270000

Mn-7

Mn	11.4456540000	8.6386330000	11.5238910000
P	10.3944620000	10.3242660000	10.4958030000
P	12.2340280000	6.6275760000	12.0791090000
O	11.6246180000	9.8215200000	14.1978580000
C	10.3542620000	9.7739190000	8.7524230000
C	10.1326570000	10.6069090000	7.6590690000
H	9.9716120000	11.6659250000	7.8194650000
C	10.1728260000	10.1031840000	6.3695040000
H	10.0233830000	10.7622570000	5.5244620000
C	10.4168670000	8.7553670000	6.1665920000
C	12.1383590000	4.5084380000	7.9954370000
H	12.1442790000	4.0555910000	7.0105790000
C	12.7263610000	3.8503460000	9.0634400000
H	13.1937890000	2.8854900000	8.9168630000
C	12.7293370000	4.4416210000	10.3148330000
H	13.2141800000	3.9443960000	11.1458170000
C	12.1563960000	5.6953630000	10.5065230000
C	13.9885710000	6.5334560000	12.5718500000
C	11.4473120000	5.5295460000	13.3198040000
C	12.0829940000	4.3704120000	13.7723780000
H	13.0775460000	4.1256050000	13.4185040000
C	11.4636870000	3.5374090000	14.6886160000
H	11.9691650000	2.6420930000	15.0281110000
C	10.2035440000	3.8546010000	15.1767610000
H	9.7222060000	3.2059120000	15.8977530000
C	9.5701030000	5.0093820000	14.7465220000
H	8.5927190000	5.2695210000	15.1330280000
C	10.1879330000	5.8420470000	13.8245730000
H	9.6996460000	6.7486330000	13.4864260000
C	11.5667750000	9.3608510000	13.1319310000
H	10.4446670000	8.3555160000	5.1594440000
O	14.1350680000	9.6027690000	10.7525290000

N	10.9069780000	7.5883050000	9.6474940000
C	10.6292660000	7.8922570000	7.2349360000
C	10.6177820000	8.4176200000	8.5252350000
C	10.8267320000	6.4232670000	7.0216250000
H	9.8464350000	5.9540030000	6.8694250000
H	11.3686320000	6.2457790000	6.0904610000
C	11.5202930000	5.7415200000	8.1601900000
C	11.5306260000	6.3259740000	9.4256970000
C	13.0744470000	9.2186500000	11.0288730000
H	10.1058640000	7.5061050000	10.2923630000
H	9.9479170000	8.0910690000	11.9290530000
C	15.0161320000	6.4067820000	11.6396090000
H	14.7807200000	6.2612260000	10.5920840000
C	16.3424410000	6.4799110000	12.0382390000
H	17.1287670000	6.3764520000	11.3012000000
C	16.6606040000	6.6911810000	13.3705680000
H	17.6962730000	6.7503570000	13.6798940000
C	15.6448060000	6.8352460000	14.3051920000
H	15.8838830000	7.0101940000	15.3464950000
C	14.3200830000	6.7594330000	13.9094920000
H	13.5330300000	6.8777390000	14.6463400000
C	8.6657400000	10.7509070000	10.9323320000
C	8.2145210000	10.4606860000	12.2183920000
H	8.8735760000	9.9433580000	12.9047470000
C	6.9345240000	10.8171800000	12.6159630000
H	6.6022600000	10.5895740000	13.6209130000
C	6.0828940000	11.4548100000	11.7276050000
H	5.0801480000	11.7250930000	12.0336770000
C	6.5192020000	11.7437540000	10.4424310000
H	5.8582160000	12.2391180000	9.7423840000
C	7.8036490000	11.4024650000	10.0505180000
H	8.1315780000	11.6434450000	9.0473100000
C	11.1739460000	11.9724360000	10.4143500000
C	12.2856660000	12.1810940000	9.5965600000
H	12.6332980000	11.3858390000	8.9475890000
C	12.9504850000	13.3956460000	9.6079730000
H	13.8081400000	13.5425080000	8.9637980000
C	12.5240370000	14.4167580000	10.4455080000
H	13.0467820000	15.3646370000	10.4559940000
C	11.4292980000	14.2156160000	11.2714720000
H	11.0931820000	15.0053200000	11.9314110000
C	10.7575060000	13.0027850000	11.2564860000

H	9.9028660000	12.8574630000	11.9066760000

$TS_{6,7}$-PE

Mn	11.2561350000	8.6605090000	11.6330690000
P	10.2878030000	10.3459100000	10.4932220000
P	12.2410140000	6.7332140000	12.2338000000
O	11.4432010000	10.0647240000	14.2028280000
C	10.2998240000	9.7203370000	8.7875000000
C	10.0876270000	10.5111170000	7.6602950000
H	9.8963260000	11.5705340000	7.7825000000
C	10.1502140000	9.9563750000	6.3940640000
H	10.0031460000	10.5775880000	5.5203470000
C	10.3975310000	8.6008640000	6.2511760000
C	12.2767910000	4.5536750000	8.1936930000
H	12.3006570000	4.0848630000	7.2168170000
C	12.9805110000	3.9959690000	9.2476180000
H	13.5612550000	3.0950860000	9.0993800000
C	12.9536840000	4.6111630000	10.4861950000
H	13.5370470000	4.2071810000	11.3047900000
C	12.2223570000	5.7819200000	10.6818150000
C	14.0200970000	6.7642550000	12.6715620000
C	11.5554440000	5.6730060000	13.5559040000
C	11.3782330000	4.2962390000	13.4446150000
H	11.6535580000	3.7825420000	12.5328650000
C	10.8119900000	3.5731420000	14.4837910000
H	10.6676820000	2.5056090000	14.3753360000
C	10.4262360000	4.2116150000	15.6526000000
H	9.9817390000	3.6447420000	16.4604990000
C	10.6040430000	5.5815720000	15.7778210000
H	10.2998600000	6.0912170000	16.6833480000
C	11.1553550000	6.3072350000	14.7339900000
H	11.2632390000	7.3817150000	14.8263710000
C	11.3726940000	9.5017650000	13.1874390000
H	10.4285090000	8.1561220000	5.2632370000
O	13.9003490000	9.6969490000	10.8653250000
C	10.5907100000	7.7856020000	7.3596230000
C	10.5759520000	8.3631760000	8.6261240000
C	10.7481090000	6.3045730000	7.2317480000
H	9.7489280000	5.8501410000	7.2208670000
H	11.1961020000	6.0418580000	6.2726090000

C	11.5335020000	5.7156980000	8.3588520000
C	11.5132410000	6.3332040000	9.6094510000
C	12.8505510000	9.2760260000	11.1302230000
C	14.9849530000	6.8723750000	11.6689450000
H	14.6843320000	6.8733890000	10.6281620000
C	16.3275910000	6.9851310000	11.9890490000
H	17.0608200000	7.0645980000	11.1965040000
C	16.7299030000	7.0028360000	13.3161810000
H	17.7794240000	7.0925230000	13.5656280000
C	15.7797830000	6.9085350000	14.3206910000
H	16.0825850000	6.9240100000	15.3599650000
C	14.4358100000	6.7897530000	14.0020230000
H	13.7088370000	6.7100920000	14.8003290000
C	8.5988850000	10.9387420000	10.8374270000
C	8.3101600000	11.3278020000	12.1481320000
H	9.0466340000	11.1748400000	12.9291660000
C	7.0927790000	11.9099010000	12.4587550000
H	6.8900130000	12.2175030000	13.4766480000
C	6.1298980000	12.0728580000	11.4735250000
H	5.1741240000	12.5194910000	11.7175480000
C	6.3841750000	11.6342770000	10.1834760000
H	5.6259450000	11.7316040000	9.4166170000
C	7.6125210000	11.0744130000	9.8642130000
H	7.7978490000	10.7375170000	8.8526370000
C	11.2287300000	11.9156980000	10.3599450000
C	12.3134410000	11.9930460000	9.4839220000
H	12.5518940000	11.1518340000	8.8452000000
C	13.0966250000	13.1334130000	9.4225960000
H	13.9306430000	13.1714140000	8.7333150000
C	12.8181470000	14.2166460000	10.2423040000
H	13.4311850000	15.1076160000	10.1955940000
C	11.7507970000	14.1489000000	11.1232840000
H	11.5235940000	14.9874020000	11.7693570000
C	10.9626300000	13.0096290000	11.1821780000
H	10.1291880000	12.9838790000	11.8714630000
H	9.6762740000	8.0522460000	12.1259180000
N	10.7601100000	7.5523810000	9.8141810000
H	9.7711820000	7.3101830000	10.0836290000
C	5.2569470000	9.1421070000	13.0618060000
C	5.5918640000	9.3542600000	14.3902130000
C	6.8445490000	8.9706630000	14.8550670000
C	7.7440220000	8.3607820000	13.9979550000

C	7.4131000000	8.1424450000	12.6621860000
C	6.1671200000	8.5493690000	12.1989090000
C	8.3023220000	7.3670980000	11.7267090000
C	8.6257330000	5.9739010000	12.2279920000
O	8.1592700000	7.5120900000	10.4820420000
H	4.2855780000	9.4476340000	12.6919870000
H	4.8833390000	9.8220120000	15.0624950000
H	7.1169620000	9.1458090000	15.8885140000
H	8.7251900000	8.0642640000	14.3543700000
H	5.9252730000	8.3833650000	11.1563550000
H	7.7252200000	5.3696330000	12.0891640000
H	8.8932010000	5.9379780000	13.2803920000
H	9.4192260000	5.5207110000	11.6337010000

$TS_{6,7}$-PE-^{i}Pr

Mn	11.3849850000	8.6566290000	11.6228730000
P	10.4507500000	10.4070690000	10.4986130000
P	12.3562760000	6.6548910000	12.1513810000
O	11.5333480000	9.8850470000	14.2837480000
C	10.4895670000	9.8069930000	8.7784940000
C	10.2804630000	10.6016730000	7.6530870000
H	10.1025280000	11.6624120000	7.7732050000
C	10.3018560000	10.0513310000	6.3830980000
H	10.1639890000	10.6819010000	5.5144900000
C	10.4800590000	8.6874850000	6.2330400000
C	11.8687420000	4.4033840000	8.1808750000
H	11.8027660000	3.9233040000	7.2113860000
C	12.4479920000	3.7385850000	9.2470050000
H	12.8381940000	2.7372820000	9.1189820000
C	12.5484900000	4.3747550000	10.4718400000
H	13.0425140000	3.8740970000	11.2948030000
C	12.0646640000	5.6694330000	10.6455050000
C	11.4765590000	9.4016830000	13.2272030000
H	10.4663120000	8.2427960000	5.2445980000
O	13.9856860000	9.8717390000	11.0317110000
C	10.6418830000	7.8601880000	7.3380030000
C	10.6895830000	8.4349740000	8.6050780000
C	10.6718390000	6.3734840000	7.1907370000
H	9.6370300000	6.0137540000	7.1227770000
H	11.1351320000	6.0878830000	6.2446430000

C	11.3473250000	5.6831110000	8.3299030000
C	11.4369740000	6.3089640000	9.5723230000
C	12.9567180000	9.3488760000	11.2108600000
H	9.7556890000	8.0675060000	12.1139390000
N	10.8336330000	7.6102240000	9.7895850000
H	9.8388410000	7.4289640000	10.0780620000
C	5.3667290000	9.4164670000	12.8681120000
C	5.6434420000	9.6430030000	14.2085040000
C	6.8254270000	9.1628240000	14.7572290000
C	7.7176730000	8.4527100000	13.9714750000
C	7.4508240000	8.2216320000	12.6249160000
C	6.2668960000	8.7135190000	12.0814550000
C	8.3450860000	7.3821370000	11.7459120000
C	8.6396360000	6.0092370000	12.3110410000
O	8.2175630000	7.4737930000	10.4935370000
H	4.4464450000	9.7873380000	12.4336760000
H	4.9439580000	10.1950400000	14.8234690000
H	7.0540020000	9.3468040000	15.7994460000
H	8.6480520000	8.0966300000	14.3990760000
H	6.0688020000	8.5300770000	11.0321920000
H	7.7177010000	5.4258810000	12.2390930000
H	8.9417000000	6.0226470000	13.3551200000
H	9.3936860000	5.5065340000	11.7073190000
C	12.7526740000	4.5438330000	14.1221410000
H	13.1266910000	3.8617070000	13.3607530000
H	13.6122860000	5.0010420000	14.6132110000
H	12.2477880000	3.9346540000	14.8741350000
C	11.7792330000	5.5841710000	13.5879730000
H	10.9277430000	5.0497530000	13.1636550000
H	10.5642400000	7.2016030000	14.3849190000
H	10.7771910000	5.8358380000	15.4842830000
C	11.2720780000	6.4513670000	14.7306060000
H	12.0855740000	6.9807330000	15.2287760000
C	14.2125980000	6.5410110000	12.3059340000
C	14.6899240000	7.4618140000	13.4199240000
H	14.1693190000	7.2767430000	14.3604080000
H	14.5429690000	8.5114840000	13.1683860000
H	15.7557060000	7.3163110000	13.6036980000
H	14.4067410000	5.5098340000	12.6118020000
H	14.7442010000	7.7147430000	10.5350990000
H	14.7514630000	5.9772520000	10.2754230000
H	16.0388450000	6.7384620000	11.1957590000

C	14.9647380000	6.7593930000	11.0024370000
C	11.7332570000	12.4367060000	11.9076760000
H	10.9786590000	12.2757940000	12.6742470000
H	12.6039410000	11.8401820000	12.1780210000
H	12.0313760000	13.4855730000	11.9526880000
C	11.2512380000	12.0957760000	10.5009450000
H	10.4425110000	12.7878100000	10.2410730000
H	12.0731900000	12.2082790000	8.4698690000
H	12.8061680000	13.2926540000	9.6369380000
C	12.3864240000	12.2934070000	9.5065590000
H	13.1948450000	11.5841140000	9.6668230000
C	8.6615010000	10.7986590000	10.7867460000
C	7.9533880000	11.6152800000	9.7212830000
H	7.9453760000	11.1183940000	8.7529950000
H	8.3893600000	12.6082800000	9.5923530000
H	6.9116130000	11.7640390000	10.0147990000
H	8.2225100000	9.7983850000	10.7827870000
H	8.7916600000	12.4574050000	12.1847100000
H	9.0211710000	10.8767750000	12.9395680000
H	7.4168180000	11.3964760000	12.4410720000
C	8.4722300000	11.4136570000	12.1653470000

$TS_{6,7}$-EG

Mn	11.4342120000	8.7076230000	11.6138990000
P	10.4193990000	10.4208960000	10.5726520000
P	12.2997430000	6.7061780000	12.1565240000
O	11.9008440000	10.0331030000	14.1894860000
C	10.2796850000	9.8182430000	8.8639910000
C	9.9807590000	10.6350380000	7.7759550000
H	9.7840670000	11.6879190000	7.9402560000
C	9.9677990000	10.1150520000	6.4937680000
H	9.7517860000	10.7557110000	5.6489330000
C	10.2399960000	8.7712650000	6.2971710000
C	12.3644310000	4.7336260000	8.0137420000
H	12.3711720000	4.3015790000	7.0198350000
C	13.1071980000	4.1517410000	9.0271830000
H	13.7029860000	3.2706670000	8.8281730000
C	13.0887720000	4.7078490000	10.2943140000
H	13.6785560000	4.2695830000	11.0906240000
C	12.3418850000	5.8556170000	10.5500930000

C	14.0226030000	6.6089500000	12.7543270000
C	11.4027880000	5.6046170000	13.3031580000
C	11.1256250000	4.2669810000	13.0308940000
H	11.4632790000	3.8182820000	12.1055660000
C	10.3931510000	3.5049430000	13.9296040000
H	10.1750010000	2.4698530000	13.6991340000
C	9.9364900000	4.0654260000	15.1121830000
H	9.3605720000	3.4695570000	15.8086240000
C	10.2076440000	5.3966500000	15.3940260000
H	9.8398600000	5.8484110000	16.3062630000
C	10.9260280000	6.1624460000	14.4918710000
H	11.1027760000	7.2119710000	14.6993550000
C	11.7119000000	9.5076640000	13.1688200000
H	10.2292060000	8.3569610000	5.2957170000
O	14.0735570000	9.6146690000	10.6754780000
C	10.5178990000	7.9290080000	7.3668720000
C	10.5532740000	8.4666900000	8.6512980000
C	10.7383790000	6.4631640000	7.1744200000
H	9.7639620000	5.9580180000	7.1834220000
H	11.1587980000	6.2621530000	6.1881970000
C	11.5952980000	5.8678450000	8.2446610000
C	11.6046630000	6.4373800000	9.5159370000
C	13.0267720000	9.2436720000	11.0159440000
C	15.0781760000	6.7937380000	11.8595220000
H	14.8748050000	6.9215360000	10.8033810000
C	16.3878720000	6.8132430000	12.3086100000
H	17.1937670000	6.9534780000	11.5995080000
C	16.6653000000	6.6582970000	13.6589040000
H	17.6893410000	6.6758520000	14.0092610000
C	15.6244870000	6.4821950000	14.5568020000
H	15.8307460000	6.3603870000	15.6125500000
C	14.3125500000	6.4566280000	14.1092940000
H	13.5117760000	6.3103540000	14.8232850000
C	8.7486630000	10.9287260000	11.0937300000
C	8.5668340000	11.2765200000	12.4351820000
H	9.4086350000	11.2431010000	13.1186320000
C	7.3196640000	11.6505660000	12.9060160000
H	7.1969330000	11.9123080000	13.9490160000
C	6.2294390000	11.6619840000	12.0478140000
H	5.2510330000	11.9410410000	12.4173590000
C	6.3945770000	11.3009310000	10.7194070000
H	5.5449020000	11.2938540000	10.0485700000

C	7.6459670000	10.9394960000	10.2421160000
H	7.7569010000	10.6474810000	9.2057460000
C	11.3079270000	12.0080420000	10.3749370000
C	12.3902110000	12.0669520000	9.4938220000
H	12.6466880000	11.2006920000	8.8961560000
C	13.1396370000	13.2240930000	9.3703360000
H	13.9725720000	13.2500120000	8.6793010000
C	12.8271580000	14.3421530000	10.1301330000
H	13.4147340000	15.2463000000	10.0352590000
C	11.7580880000	14.2944460000	11.0098730000
H	11.5033500000	15.1622730000	11.6048680000
C	11.0026600000	13.1375270000	11.1317180000
H	10.1646870000	13.1253600000	11.8160750000
H	9.8662160000	8.1773310000	12.1560020000
N	10.8273990000	7.6266310000	9.7998740000
H	9.8876030000	7.3039770000	10.1373250000
C	7.6429600000	8.0473880000	12.8090440000
C	8.5623680000	7.2099050000	11.9546830000
O	8.2089950000	8.4230190000	14.0411560000
O	8.3475510000	7.0477590000	10.7364120000
H	6.7772840000	7.4181550000	13.0428990000
H	7.2689360000	8.8982440000	12.2282740000
H	9.0825080000	6.4248640000	12.5356050000
H	9.0777620000	8.7945020000	13.8255880000

$TS_{6,7}$-IV

Mn	11.1827170000	8.6799320000	11.6017730000
P	10.2833040000	10.4196700000	10.3665210000
P	12.2608150000	6.8068000000	12.1954890000
O	11.5786150000	10.0399620000	14.1740400000
C	10.4193480000	9.7366510000	8.6858900000
C	10.2117130000	10.4883570000	7.5290550000
H	10.0193150000	11.5507530000	7.6132340000
C	10.2321460000	9.8849930000	6.2855410000
H	10.0934370000	10.4783780000	5.3913820000
C	10.4049860000	8.5132800000	6.1906400000
C	12.2457330000	4.5498140000	8.1941950000
H	12.2383730000	4.0526070000	7.2315220000
C	13.0237560000	4.0541430000	9.2269250000
H	13.6318170000	3.1720230000	9.0755280000

C	13.0325580000	4.7025720000	10.4483400000
H	13.6650580000	4.3407810000	11.2495440000
C	12.2634670000	5.8484920000	10.6502060000
C	14.0333020000	6.9312210000	12.6309950000
C	11.6224060000	5.7162640000	13.5150460000
C	11.5537120000	4.3277320000	13.4166060000
H	11.8535000000	3.8279310000	12.5051990000
C	11.0706380000	3.5715710000	14.4725700000
H	11.0104290000	2.4951930000	14.3741720000
C	10.6544130000	4.1874430000	15.6427960000
H	10.2735340000	3.5934110000	16.4636340000
C	10.7147950000	5.5679850000	15.7516680000
H	10.3794370000	6.0633510000	16.6545210000
C	11.1888950000	6.3267940000	14.6935810000
H	11.2117690000	7.4067890000	14.7822640000
C	11.4032470000	9.5115250000	13.1561030000
H	10.3947720000	8.0294370000	5.2211960000
O	13.7697210000	9.7217250000	10.6628510000
C	10.5553980000	7.7337930000	7.3288700000
C	10.5907160000	8.3593490000	8.5739030000
C	10.6223610000	6.2419450000	7.2685170000
H	9.6048820000	5.8422240000	7.3699160000
H	10.9763650000	5.9044350000	6.2946380000
C	11.4721240000	5.6900840000	8.3654810000
C	11.4919820000	6.3432140000	9.5967100000
C	12.7411580000	9.3071990000	11.0053210000
C	15.0073660000	7.0091760000	11.6351830000
H	14.7233930000	6.9267870000	10.5932070000
C	16.3393830000	7.1992010000	11.9646660000
H	17.0815810000	7.2533280000	11.1783960000
C	16.7189090000	7.3261610000	13.2922600000
H	17.7598380000	7.4764060000	13.5484590000
C	15.7575750000	7.2640840000	14.2891480000
H	16.0427950000	7.3663260000	15.3283840000
C	14.4252540000	7.0680300000	13.9624360000
H	13.6880180000	7.0173080000	14.7540450000
C	8.6033860000	11.1601380000	10.2611330000
C	8.2573230000	12.3109270000	10.9681320000
H	9.0036270000	12.8461810000	11.5395650000
C	6.9559780000	12.7891450000	10.9488740000
H	6.7094350000	13.6859000000	11.5031490000
C	5.9777270000	12.1262210000	10.2239930000

H	4.9614480000	12.4991960000	10.2119670000
C	6.3107980000	10.9831650000	9.5117630000
H	5.5550930000	10.4534790000	8.9454090000
C	7.6096420000	10.5027340000	9.5303450000
H	7.8430760000	9.5955190000	8.9896970000
C	11.3624430000	11.8908380000	10.4607940000
C	12.1486640000	12.3524270000	9.4085750000
H	12.1187870000	11.8584490000	8.4469350000
C	13.0103590000	13.4244210000	9.5899150000
H	13.6211490000	13.7621030000	8.7622500000
C	13.1002040000	14.0513290000	10.8217980000
H	13.7762650000	14.8851870000	10.9605930000
C	12.3334570000	13.5910400000	11.8823300000
H	12.4088850000	14.0593580000	12.8552570000
C	11.4812580000	12.5144510000	11.7058740000
H	10.9135010000	12.1425430000	12.5508560000
H	9.6812200000	7.9986870000	12.2596920000
N	10.7121340000	7.5593800000	9.7849100000
H	9.7390560000	7.3102180000	10.0485570000
C	7.3300900000	7.9880670000	13.0217530000
C	8.1928210000	7.3159440000	11.9065960000
C	8.8057920000	5.9405080000	12.1386370000
O	7.9546580000	7.5329790000	10.7020280000
O	8.4396950000	5.1006280000	12.9156620000
H	9.5883480000	5.7152060000	11.3756070000
C	7.4404880000	8.4459620000	15.5404630000
C	7.8199870000	7.5137650000	14.4169630000
O	8.2680990000	9.1844970000	16.0413840000
C	7.5085930000	9.4959560000	12.9238770000
C	6.0252110000	8.4913400000	16.0009510000
C	5.1679020000	7.3951390000	15.9197700000
C	3.8590460000	7.4936540000	16.3631660000
C	3.3861870000	8.6945080000	16.8703320000
C	4.2317900000	9.7937930000	16.9546790000
C	5.5447330000	9.6880990000	16.5351850000
C	5.8777630000	7.5810280000	12.7485240000
C	5.4993420000	6.2382550000	12.8075440000
C	4.1940890000	5.8457630000	12.5640070000
C	3.2259290000	6.7891730000	12.2514900000
C	3.5853600000	8.1243680000	12.1828160000
C	4.8960550000	8.5144680000	12.4227350000
H	7.4728250000	6.5014110000	14.6176010000

H	8.9094230000	7.4876380000	14.3910080000
H	6.8563920000	10.0210020000	13.6228860000
H	7.2716420000	9.8414030000	11.9245540000
H	8.5359600000	9.7744820000	13.1562180000
H	5.5267710000	6.4555120000	15.5216580000
H	3.2050550000	6.6329690000	16.3026820000
H	2.3585030000	8.7749570000	17.2015970000
H	3.8632060000	10.7322410000	17.3489910000
H	6.2222100000	10.5305830000	16.5998320000
H	6.2406390000	5.4900450000	13.0584550000
H	3.9332290000	4.7959380000	12.6186810000
H	2.2041040000	6.4853110000	12.0626870000
H	2.8436820000	8.8757410000	11.9412490000
H	5.1418630000	9.5651910000	12.3597580000

$TS_{6,7}$-VI

Mn	10.9561070000	8.7928470000	11.5758270000
P	10.3147740000	10.5118520000	10.1479080000
P	11.8627100000	6.9030510000	12.3728430000
O	10.9708960000	10.1910560000	14.1566030000
C	10.5955620000	9.7256960000	8.5307890000
C	10.5472150000	10.4210120000	7.3217720000
H	10.3846260000	11.4916680000	7.3313010000
C	10.6872650000	9.7539900000	6.1192560000
H	10.6711840000	10.3042740000	5.1876820000
C	10.8257100000	8.3752640000	6.1120770000
C	12.2902860000	4.4643520000	8.5118700000
H	12.3834080000	3.9187980000	7.5802120000
C	12.8988440000	3.9871640000	9.6611660000
H	13.4768180000	3.0728630000	9.6320640000
C	12.7719420000	4.6918100000	10.8440870000
H	13.2627180000	4.3387560000	11.7430750000
C	12.0417180000	5.8788910000	10.8863990000
C	13.5710110000	6.9793320000	13.0359840000
C	11.0195590000	5.8702230000	13.6231970000
C	10.7654340000	4.5086870000	13.4664680000
H	11.0554030000	4.0009200000	12.5564080000
C	10.1065910000	3.7973230000	14.4585940000
H	9.9065460000	2.7430780000	14.3150800000
C	9.7063910000	4.4295200000	15.6255520000

H	9.1989900000	3.8708370000	16.4018280000
C	9.9504020000	5.7854410000	15.7918520000
H	9.6333460000	6.2901870000	16.6957780000
C	10.5853520000	6.5023300000	14.7913370000
H	10.7404020000	7.5678600000	14.9154380000
C	10.9576180000	9.6544670000	13.1245600000
H	10.9126030000	7.8415030000	5.1729990000
O	13.6986360000	9.7033190000	11.0031780000
C	10.8208620000	7.6526370000	7.2966930000
C	10.7305550000	8.3398070000	8.5067090000
C	10.8568340000	6.1586990000	7.3140210000
H	9.8251170000	5.7818810000	7.3113070000
H	11.3130820000	5.7680890000	6.4045210000
C	11.5539170000	5.6409550000	8.5288060000
C	11.4452150000	6.3534850000	9.7207900000
C	12.6148700000	9.3381320000	11.2013230000
C	14.6582310000	6.9892700000	12.1607910000
H	14.4952190000	6.8875290000	11.0950620000
C	15.9502010000	7.1364400000	12.6378330000
H	16.7790490000	7.1381810000	11.9414030000
C	16.1798830000	7.2870990000	13.9967910000
H	17.1896450000	7.4033660000	14.3690720000
C	15.1076680000	7.2916110000	14.8748410000
H	15.2742900000	7.4120890000	15.9377010000
C	13.8145650000	7.1396370000	14.3994800000
H	12.9934790000	7.1405320000	15.1041560000
C	8.7304290000	11.3823450000	9.8073200000
C	8.4022690000	12.5770220000	10.4473220000
H	9.1093200000	13.0515410000	11.1144240000
C	7.1685700000	13.1754670000	10.2438370000
H	6.9347150000	14.1014000000	10.7536500000
C	6.2408920000	12.5929850000	9.3946930000
H	5.2769050000	13.0597670000	9.2377280000
C	6.5585800000	11.4087850000	8.7457300000
H	5.8428360000	10.9442130000	8.0787590000
C	7.7879310000	10.8048490000	8.9528820000
H	8.0049820000	9.8671460000	8.4609340000
C	11.4895080000	11.9074660000	10.2909520000
C	12.3944400000	12.2760460000	9.2996550000
H	12.4119570000	11.7522970000	8.3536610000
C	13.3125880000	13.2917570000	9.5243110000
H	14.0152930000	13.5558920000	8.7441600000

C	13.3407520000	13.9555040000	10.7396100000
H	14.0606470000	14.7450880000	10.9127530000
C	12.4522370000	13.5895300000	11.7402230000
H	12.4752420000	14.0882590000	12.7006560000
C	11.5441380000	12.5679530000	11.5214380000
H	10.8789460000	12.2680760000	12.3229430000
H	9.3402120000	8.2050380000	11.9556520000
N	10.6984040000	7.6010610000	9.7581220000
H	9.7081600000	7.3823910000	9.9466190000
C	7.0449240000	8.4756030000	12.5876590000
C	7.8694440000	7.6951210000	11.5073290000
O	7.7555270000	7.9971600000	10.2972570000
C	6.7816150000	8.6223150000	15.1779720000
C	7.5067890000	8.0435870000	13.9844960000
O	6.3437180000	9.7551360000	15.1930100000
C	7.2358610000	9.9713460000	12.3803270000
C	6.6720600000	7.7855800000	16.4149910000
C	6.6742080000	6.3902560000	16.4152270000
C	6.5210710000	5.6852210000	17.5992720000
C	6.3929970000	6.3633630000	18.8014920000
C	6.3983110000	7.7524630000	18.8156560000
C	6.5219740000	8.4543700000	17.6317960000
C	5.5837240000	8.0990730000	12.3199400000
C	4.8500150000	7.2701550000	13.1662490000
C	3.5277640000	6.9465000000	12.8941600000
C	2.9082510000	7.4386260000	11.7582310000
C	3.6275460000	8.2550140000	10.8973670000
C	4.9447400000	8.5792380000	11.1741460000
H	7.5338590000	6.9593270000	14.0703610000
H	8.5525010000	8.3561660000	14.0948810000
H	6.5426020000	10.5463060000	12.9861370000
H	7.0825990000	10.2189580000	11.3367020000
H	8.2522110000	10.2648280000	12.6494710000
H	6.7771040000	5.8394490000	15.4883830000
H	6.5026700000	4.6028000000	17.5814720000
H	6.2849880000	5.8112070000	19.7265020000
H	6.3001190000	8.2854750000	19.7527310000
H	6.5095330000	9.5366500000	17.6210840000
H	5.3014630000	6.8600480000	14.0607500000
H	2.9843100000	6.3072460000	13.5787500000
H	1.8764870000	7.1896860000	11.5450280000
H	3.1594210000	8.6452840000	10.0019090000

H	5.4961140000	9.2035760000	10.4840460000
C	8.0466720000	6.1977890000	11.7808700000
O	8.5290760000	5.5481210000	10.6319840000
H	7.0555770000	5.8069140000	12.0662130000
H	8.7262780000	5.9903240000	12.6022570000
H	8.1144910000	6.0454280000	9.9047000000

$TS_{7,6}$

Mn	11.3144590000	8.5881290000	11.5416290000
P	10.3147720000	10.2933590000	10.4676450000
P	12.2559140000	6.6205210000	12.0867270000
O	10.8906520000	9.5695990000	14.2738460000
C	10.1471750000	9.6692250000	8.7705160000
C	9.6105710000	10.4112920000	7.7219270000
H	9.2434220000	11.4130080000	7.9121660000
C	9.5682190000	9.8891680000	6.4428210000
H	9.1648040000	10.4720080000	5.6256000000
C	10.0555190000	8.6085460000	6.2171350000
C	12.2545820000	4.5051570000	8.0246860000
H	12.2588000000	4.0362120000	7.0466030000
C	12.8081380000	3.8367620000	9.1087150000
H	13.2445210000	2.8552100000	8.9804840000
C	12.7985610000	4.4425080000	10.3511390000
H	13.2339630000	3.9369270000	11.2050280000
C	12.2556470000	5.7141790000	10.5131450000
C	13.9936290000	6.6766440000	12.6288920000
C	11.5060370000	5.4872300000	13.3112960000
C	12.2227410000	4.4183830000	13.8537270000
H	13.2603690000	4.2690100000	13.5787970000
C	11.6240190000	3.5539070000	14.7551320000
H	12.1921380000	2.7291950000	15.1665810000
C	10.3037310000	3.7490360000	15.1352580000
H	9.8385620000	3.0760890000	15.8443710000
C	9.5866540000	4.8138640000	14.6128160000
H	8.5601650000	4.9787320000	14.9148050000
C	10.1842840000	5.6781600000	13.7079970000
H	9.6268800000	6.5156240000	13.3051430000
C	11.0727210000	9.1884180000	13.1925880000
H	10.0275200000	8.1930410000	5.2155470000
O	13.9573710000	9.8936650000	11.3541690000

N	11.1026980000	7.6285250000	9.6176800000
C	10.5673840000	7.8279030000	7.2417540000
C	10.6159180000	8.3640670000	8.5368090000
C	11.0345000000	6.4279470000	6.9752110000
H	10.1840660000	5.8237980000	6.6379250000
H	11.7245300000	6.4233540000	6.1255780000
C	11.6757570000	5.7578650000	8.1537150000
C	11.6780910000	6.3741870000	9.4137280000
C	12.9233070000	9.3773360000	11.4122230000
H	10.2615470000	7.5930010000	10.5490930000
H	9.8124580000	7.7920470000	11.5340900000
C	15.0592720000	6.4930010000	11.7520630000
H	14.8703820000	6.2098930000	10.7236940000
C	16.3645250000	6.6818340000	12.1838810000
H	17.1834310000	6.5347610000	11.4911040000
C	16.6196050000	7.0610150000	13.4917430000
H	17.6383440000	7.2103540000	13.8258270000
C	15.5626740000	7.2565460000	14.3708550000
H	15.7534900000	7.5606840000	15.3921720000
C	14.2601350000	7.0693680000	13.9427450000
H	13.4386560000	7.2346320000	14.6320010000
C	8.6562210000	10.8997140000	10.9427100000
C	7.8539270000	10.1221520000	11.7750140000
H	8.2375600000	9.1805530000	12.1498420000
C	6.5823340000	10.5467720000	12.1285290000
H	5.9731090000	9.9332370000	12.7800310000
C	6.0972340000	11.7565260000	11.6569740000
H	5.1058220000	12.0905220000	11.9356900000
C	6.8902190000	12.5443040000	10.8345620000
H	6.5200080000	13.4941830000	10.4699890000
C	8.1617010000	12.1221450000	10.4829530000
H	8.7803030000	12.7541410000	9.8565120000
C	11.2694190000	11.8383260000	10.3328740000
C	12.0854290000	12.1171370000	9.2397210000
H	12.0885110000	11.4521400000	8.3845730000
C	12.9056170000	13.2363140000	9.2426430000
H	13.5347280000	13.4408320000	8.3855770000
C	12.9239010000	14.0854750000	10.3373330000
H	13.5663110000	14.9567280000	10.3391280000
C	12.1201600000	13.8109210000	11.4358030000
H	12.1344760000	14.4659250000	12.2976550000
C	11.3019600000	12.6946970000	11.4358740000

H	10.6847900000	12.4802870000	12.3020890000

TS$_{7,6}$-IV

Mn	11.3713720000	8.6777920000	11.5604230000
P	10.1842070000	10.1351060000	10.3292450000
P	12.4762960000	6.8654730000	12.3074450000
O	11.5256210000	10.2770670000	14.0158070000
C	10.2400470000	9.3915780000	8.6717660000
C	9.9074970000	10.0723030000	7.5026870000
H	9.5714680000	11.1006560000	7.5642760000
C	10.0354780000	9.4526580000	6.2721110000
H	9.7932630000	9.9900210000	5.3646730000
C	10.4805840000	8.1425850000	6.2078750000
C	12.9764380000	4.5594730000	8.3716630000
H	13.0882900000	4.0487370000	7.4223470000
C	13.7314770000	4.1656160000	9.4635840000
H	14.4399660000	3.3530290000	9.3709380000
C	13.5817160000	4.8237100000	10.6714690000
H	14.1812360000	4.5359860000	11.5269040000
C	12.6871090000	5.8851830000	10.7908080000
C	14.1795870000	7.0602410000	12.9393990000
C	11.7047470000	5.7580400000	13.5373100000
C	11.5885790000	4.3791180000	13.3734070000
H	11.9787450000	3.9015670000	12.4839250000
C	10.9538070000	3.6083660000	14.3367740000
H	10.8618780000	2.5396130000	14.1906800000
C	10.4342290000	4.2018820000	15.4764780000
H	9.9341530000	3.5985040000	16.2232950000
C	10.5391090000	5.5747490000	15.6475090000
H	10.1162790000	6.0508800000	16.5229550000
C	11.1601690000	6.3470090000	14.6807340000
H	11.2162710000	7.4227030000	14.8075710000
C	11.4627810000	9.6416860000	13.0438350000
H	10.5787710000	7.6496270000	5.2477080000
O	13.9008420000	9.8678210000	10.6340670000
C	10.7968120000	7.4328130000	7.3599190000
C	10.6930650000	8.0748700000	8.5913430000
C	11.2011150000	5.9948040000	7.3098680000
H	10.2949780000	5.3754930000	7.3198560000
H	11.6965060000	5.7656590000	6.3654890000

C	12.0630570000	5.6024980000	8.4663900000
C	11.9349560000	6.2743490000	9.6803300000
C	12.9019050000	9.3797480000	10.9700530000
C	15.2232750000	7.3048950000	12.0449400000
H	15.0314050000	7.3066690000	10.9789130000
C	16.5063540000	7.5450660000	12.5067010000
H	17.3036190000	7.7287070000	11.7977810000
C	16.7675720000	7.5543060000	13.8690640000
H	17.7705180000	7.7442240000	14.2292710000
C	15.7375560000	7.3199500000	14.7659790000
H	15.9313480000	7.3245160000	15.8310540000
C	14.4529000000	7.0737320000	14.3062150000
H	13.6621790000	6.8843860000	15.0209840000
C	8.4244860000	10.4338650000	10.7037180000
C	8.0966920000	10.8540800000	11.9962930000
H	8.8765030000	10.9566500000	12.7431400000
C	6.7836580000	11.1303490000	12.3377860000
H	6.5474260000	11.4545840000	13.3433290000
C	5.7730740000	10.9630700000	11.4000950000
H	4.7434040000	11.1681200000	11.6677380000
C	6.0824740000	10.5090210000	10.1275990000
H	5.2965730000	10.3594340000	9.3974160000
C	7.4003700000	10.2490700000	9.7782840000
H	7.6262260000	9.8932620000	8.7814540000
C	10.8442150000	11.8182470000	10.0449170000
C	11.9518520000	11.9735460000	9.2080380000
H	12.3610470000	11.1150370000	8.6897680000
C	12.5337900000	13.2164400000	9.0276290000
H	13.3894810000	13.3152640000	8.3719230000
C	12.0254340000	14.3270550000	9.6854500000
H	12.4818170000	15.2986470000	9.5460120000
C	10.9299780000	14.1846510000	10.5214250000
H	10.5235340000	15.0450800000	11.0376180000
C	10.3422960000	12.9410860000	10.7001270000
H	9.4808420000	12.8549660000	11.3491640000
H	9.8597190000	8.0112000000	12.0955140000
N	11.0026390000	7.3744960000	9.8219090000
H	10.0909910000	6.9708430000	10.1411280000
C	7.6921190000	7.4776530000	12.8076170000
C	8.7252470000	6.8159050000	11.9138080000
O	8.2718010000	7.8988940000	14.0219410000
O	8.5256480000	6.6297690000	10.6984210000

H	7.2519790000	8.3226620000	12.2592630000
H	9.3995270000	6.1443680000	12.4778500000
H	9.1167420000	8.3088580000	13.7831120000
C	4.4111350000	5.5656690000	12.1296090000
O	4.3371940000	4.4158620000	12.5221410000
C	7.1098870000	5.1958480000	13.7231170000
C	3.1730530000	6.3826720000	11.9761130000
C	3.0917940000	7.4964090000	11.1395290000
C	1.9178740000	8.2263210000	11.0518200000
C	0.8174300000	7.8667240000	11.8160840000
C	0.8868910000	6.7596470000	12.6518690000
C	2.0509250000	6.0168820000	12.7205550000
C	5.6215710000	7.1977600000	14.1586430000
C	4.9837520000	8.3806980000	13.7867170000
C	4.1192960000	9.0380630000	14.6440720000
C	5.3649060000	6.6971890000	15.4311830000
H	6.3195320000	4.5139400000	14.0292630000
H	7.7553590000	5.3937020000	14.5782560000
H	7.7005370000	4.6748460000	12.9698980000
H	3.9438550000	7.7902610000	10.5404900000
H	1.8627900000	9.0803340000	10.3881890000
H	-0.0946990000	8.4473210000	11.7597660000
H	0.0303130000	6.4791350000	13.2514500000
H	2.1235000000	5.1501050000	13.3652590000
H	3.6337620000	9.9519460000	14.3216400000
H	5.8426450000	5.7852260000	15.7618700000
C	4.4961840000	7.3507920000	16.2956690000
H	3.1903340000	9.0300870000	16.5821400000
C	3.8683520000	8.5224590000	15.9079530000
H	4.3132570000	6.9374000000	17.2797450000
H	5.1624980000	8.7898120000	12.7998610000
C	5.7526850000	6.1995930000	11.8505060000
H	5.6509180000	7.1273900000	11.2896840000
H	6.3386600000	5.5181000000	11.2344510000
C	6.5386470000	6.4906470000	13.1605580000

$TS_{7,6}$-V

Mn	11.3389430000	8.4538390000	11.6835920000
P	10.2265970000	10.2280820000	10.8502720000
P	12.3751250000	6.4738670000	11.9634120000

O	11.6624480000	9.3925920000	14.4442330000
C	9.9969820000	9.7747630000	9.1016700000
C	9.5803120000	10.6751680000	8.1237120000
H	9.3117610000	11.6847080000	8.4106970000
C	9.5550930000	10.3018870000	6.7918180000
H	9.2435290000	11.0093780000	6.0347100000
C	9.9488660000	9.0244240000	6.4314360000
C	12.4869660000	5.0079410000	7.6185990000
H	12.5003410000	4.6929530000	6.5816110000
C	13.2716390000	4.3517890000	8.5516330000
H	13.9088240000	3.5321120000	8.2466380000
C	13.2371980000	4.7503870000	9.8768330000
H	13.8520410000	4.2483640000	10.6144740000
C	12.4359030000	5.8193420000	10.2689680000
C	14.1318840000	6.4589760000	12.4772460000
C	11.6202920000	5.1855280000	13.0144900000
C	11.2558600000	3.9248670000	12.5495650000
H	11.4603310000	3.6452860000	11.5243130000
C	10.5936190000	3.0328610000	13.3819830000
H	10.3042480000	2.0617680000	13.0003470000
C	10.2981850000	3.3817410000	14.6912740000
H	9.7809570000	2.6831540000	15.3377360000
C	10.6553900000	4.6369530000	15.1660690000
H	10.4220420000	4.9241320000	16.1839930000
C	11.2959970000	5.5364780000	14.3293270000
H	11.5242640000	6.5327020000	14.6920080000
C	11.5299220000	9.0298320000	13.3478880000
H	9.9388050000	8.7274070000	5.3891660000
O	13.9184150000	9.6922680000	10.9971340000
C	10.3566900000	8.1003550000	7.3860400000
C	10.3851040000	8.4889520000	8.7232850000
C	10.7303050000	6.7038770000	7.0064980000
H	9.8162640000	6.0994020000	6.9460720000
H	11.1559660000	6.6807930000	6.0021880000
C	11.6573500000	6.0612310000	7.9871920000
C	11.6590390000	6.4778310000	9.3146170000
C	12.8958410000	9.1889760000	11.2218060000
C	15.1107160000	6.8379370000	11.5560930000
H	14.8303070000	7.0694110000	10.5359520000
C	16.4407180000	6.9223350000	11.9308310000
H	17.1836960000	7.2159680000	11.2003200000
C	16.8177860000	6.6372900000	13.2349200000

H	17.8574070000	6.7059290000	13.5285200000
C	15.8551670000	6.2648310000	14.1591630000
H	16.1383100000	6.0384070000	15.1792470000
C	14.5229610000	6.1752030000	13.7845790000
H	13.7897340000	5.8724170000	14.5203020000
C	8.6194850000	10.7837060000	11.5100380000
C	8.5595310000	11.0740850000	12.8760680000
H	9.4128540000	10.8615650000	13.5105330000
C	7.4273270000	11.6537970000	13.4268350000
H	7.4042880000	11.8816580000	14.4842670000
C	6.3232320000	11.9130100000	12.6278610000
H	5.4354130000	12.3593580000	13.0574050000
C	6.3497330000	11.5686260000	11.2849280000
H	5.4791890000	11.7363980000	10.6634430000
C	7.4912600000	11.0137200000	10.7260100000
H	7.4964240000	10.7520020000	9.6760890000
C	11.1287660000	11.8215010000	10.6857280000
C	12.1460720000	11.9165230000	9.7329500000
H	12.3528340000	11.0769270000	9.0799490000
C	12.8989120000	13.0711550000	9.6101310000
H	13.6805730000	13.1222140000	8.8628070000
C	12.6561660000	14.1541630000	10.4427020000
H	13.2456730000	15.0571250000	10.3481080000
C	11.6544060000	14.0710080000	11.3953600000
H	11.4536760000	14.9099080000	12.0496580000
C	10.8956320000	12.9157390000	11.5161420000
H	10.1112500000	12.8796940000	12.2600000000
H	9.8188730000	7.7643530000	12.1898090000
N	10.7982020000	7.5594760000	9.7554540000
H	9.9456950000	7.0780040000	10.0914590000
C	7.5657990000	7.1481070000	12.0260660000
C	8.8354740000	6.3025540000	11.9326010000
H	9.2565210000	5.9541880000	12.8839160000
O	9.1193030000	5.7467850000	10.8701750000
C	6.8190470000	7.1848790000	13.3821890000
C	7.2064260000	7.9035640000	15.7971780000
C	7.8574690000	7.4038540000	14.5280180000
O	7.2402170000	9.0843520000	16.0855370000
C	5.8499100000	8.3549090000	13.3399810000
C	6.4611860000	6.9422430000	16.6546270000
C	6.7687350000	5.5837050000	16.7172140000
C	6.0361930000	4.7343450000	17.5300730000

C	4.9765760000	5.2291520000	18.2750030000
C	4.6617030000	6.5812910000	18.2214340000
C	5.4061390000	7.4323250000	17.4265480000
C	6.1042180000	5.8346360000	13.5039570000
C	6.8265700000	4.6411480000	13.5856940000
C	6.1912300000	3.4152640000	13.6876000000
C	4.8068430000	3.3470020000	13.7164430000
C	4.0733920000	4.5190100000	13.6331400000
C	4.7131800000	5.7446930000	13.5179300000
H	8.4129810000	6.4842620000	14.7169960000
H	5.2156920000	8.3860960000	14.2257310000
H	6.4054490000	9.2877210000	13.2991260000
H	7.5922870000	5.1859990000	16.1387130000
H	6.2885210000	3.6823890000	17.5742950000
H	4.3951680000	4.5619520000	18.8986680000
H	3.8335180000	6.9682050000	18.8012590000
H	5.1805720000	8.4902260000	17.3757080000
H	7.9093700000	4.6574150000	13.5993880000
H	6.7864800000	2.5121990000	13.7489780000
H	4.3059210000	2.3913070000	13.8016820000
H	2.9913880000	4.4853380000	13.6549510000
H	4.1113840000	6.6399200000	13.4506500000
H	5.2137860000	8.3104730000	12.4595120000
H	8.5736150000	8.1640580000	14.2112270000
O	7.1267860000	7.6797660000	11.0374590000

H_2

H	-5.8397390000	-0.7819130000	0.0000000000
H	-5.0989760000	-0.8555830000	0.0000000000

NH_3

N	0.9077520000	0.0482530000	0.0788800000
H	0.6005360000	0.8636800000	-0.4375100000
H	0.6005390000	-0.7502410000	-0.4633300000
H	1.9186430000	0.0492680000	0.0155680000

H_2O

O	-8.2263680000	-1.6377580000	6.2519170000
H	-7.2659870000	-1.5903900000	6.2468900000
H	-8.5015820000	-0.7215660000	6.1546970000

PE

C	-0.7993550000	0.6919760000	0.8839490000
C	-1.3569820000	-0.0095900000	-0.1751390000
C	-0.5376660000	-0.7509970000	-1.0139000000
C	0.8301390000	-0.7899040000	-0.7912760000
C	1.3992160000	-0.0882520000	0.2669240000
C	0.5693900000	0.6548400000	1.1017740000
C	2.8948180000	-0.1006310000	0.4755370000
C	3.5695970000	1.0674530000	-0.2088010000
O	3.2502290000	-0.0167800000	1.8517440000
H	-1.4335490000	1.2719650000	1.5428840000
H	-2.4259810000	0.0174950000	-0.3436810000
H	-0.9660040000	-1.3057250000	-1.8393380000
H	1.4692790000	-1.3761160000	-1.4432040000
H	1.0086020000	1.2003820000	1.9286590000
H	3.2893180000	-1.0335810000	0.0475250000
H	3.1962670000	2.0095500000	0.1932830000
H	3.3752770000	1.0536930000	-1.2800300000
H	4.6469350000	1.0313200000	-0.0528100000
H	2.8010470000	-0.7321130000	2.3151600000

Intermediate I

C	-0.7317450000	1.1786430000	0.1249510000
C	-1.3530150000	-0.0609130000	0.1516440000
C	-0.5968510000	-1.2200340000	0.0289530000
C	0.7743170000	-1.1373070000	-0.1197000000
C	1.4122850000	0.1037850000	-0.1486150000
C	0.6430670000	1.2610640000	-0.0244190000
C	2.8922460000	0.1422030000	-0.3112260000
C	3.5701630000	1.4816470000	-0.3472640000
O	3.5321500000	-0.8865410000	-0.4120340000
H	-1.3196030000	2.0824610000	0.2203400000
H	-2.4275360000	-0.1249010000	0.2681930000

H	-1.0820050000	-2.1875260000	0.0498610000
H	1.3830460000	-2.0272200000	-0.2168330000
H	1.1180600000	2.2335550000	-0.0443590000
H	3.1856450000	2.0937880000	-1.1638670000
H	4.6399420000	1.3487030000	-0.4760310000
H	3.3886890000	2.0366010000	0.5739980000

EG

C	1.0333290000	0.0109790000	-0.1110570000
C	0.4787180000	0.2732200000	-1.4952970000
O	2.4462230000	0.0322310000	-0.0859640000
O	-0.9341610000	0.3026620000	-1.5123170000
H	0.6377540000	-0.9412890000	0.2727250000
H	0.6991270000	0.7898860000	0.5758550000
H	0.8750460000	-0.4728310000	-2.2003970000
H	0.8135800000	1.2493770000	-1.8492490000
H	2.7617940000	-0.6867550000	-0.6435030000
H	-1.2504980000	-0.5705200000	-1.2577320000

Intermediate III

C	1.0313820000	0.1167920000	-0.0706040000
C	0.4455930000	1.2622860000	-0.8544490000
O	2.4356420000	0.1131220000	-0.0470050000
O	-0.7219060000	1.3766960000	-1.1104240000
H	0.6012400000	-0.8232640000	-0.4362010000
H	0.6927410000	0.2222160000	0.9641720000
H	1.1995930000	2.0200430000	-1.1791310000
H	2.7526290000	-0.1553990000	-0.9151090000

Intermediate IX

C	0.1217250000	-0.9435930000	0.2901900000
C	0.1930180000	0.4646100000	0.8336430000
O	-0.1681160000	-1.8595100000	1.0395530000
C	-1.1849320000	1.1514620000	0.9562470000
C	-2.0575170000	0.4650510000	1.9931570000
O	-0.8477460000	2.4612630000	1.4263440000

C	0.3675060000	-1.2044980000	-1.1544400000
C	0.7608370000	-0.2168770000	-2.0587340000
C	0.9265270000	-0.5173880000	-3.4008940000
C	0.7030510000	-1.8069120000	-3.8575980000
C	0.3168260000	-2.8008890000	-2.9663330000
C	0.1519490000	-2.5013640000	-1.6285350000
C	-1.8511260000	1.2343450000	-0.4121930000
C	-1.5887800000	2.3160640000	-1.2496190000
C	-2.1262980000	2.3746510000	-2.5264690000
C	-2.9327910000	1.3472440000	-2.9925550000
C	-3.2007420000	0.2646030000	-2.1691900000
C	-2.6668700000	0.2101590000	-0.8903350000
H	0.8279720000	1.1125720000	0.2320780000
H	0.6223660000	0.4110640000	1.8341380000
H	-2.2342780000	-0.5839170000	1.7674100000
H	-3.0254200000	0.9633750000	2.0669240000
H	-1.5696050000	0.5125780000	2.9652900000
H	-1.6755300000	2.9318860000	1.5816010000
H	0.9327020000	0.7965860000	-1.7228440000
H	1.2274260000	0.2594920000	-4.0919370000
H	0.8293660000	-2.0391140000	-4.9075590000
H	0.1421390000	-3.8085720000	-3.3211940000
H	-0.1558000000	-3.2587580000	-0.9192200000
H	-0.9516080000	3.1137990000	-0.8888650000
H	-1.9112990000	3.2260070000	-3.1603790000
H	-3.3507990000	1.3905940000	-3.9901150000
H	-3.8285000000	-0.5441790000	-2.5215420000
H	-2.8811380000	-0.6493420000	-0.2668000000

Intermediate II

C	-0.8815320000	0.6526500000	1.0563500000
C	-1.4263260000	-0.5371710000	0.5954120000
C	-0.6331440000	-1.4429650000	-0.0979010000
C	0.7009980000	-1.1607020000	-0.3252640000
C	1.2541040000	0.0415160000	0.1164130000
C	0.4497160000	0.9446930000	0.8109040000
C	2.7032670000	0.2907550000	-0.1075200000
C	3.2583500000	1.6566810000	-0.0509560000
O	3.4679120000	-0.6468390000	-0.2839000000
C	2.8238560000	2.7726040000	-0.6662820000

C	3.5972100000	4.0264140000	-0.5635830000
C	1.5834100000	2.8242920000	-1.5027320000
C	4.9729570000	4.0326990000	-0.3061920000
C	5.6740010000	5.2190180000	-0.1906930000
C	5.0217340000	6.4362110000	-0.3299700000
C	3.6621560000	6.4513230000	-0.5982370000
C	2.9621080000	5.2630030000	-0.7241190000
H	-1.4953760000	1.3521060000	1.6092620000
H	-2.4692370000	-0.7618110000	0.7801930000
H	-1.0587080000	-2.3716890000	-0.4559740000
H	1.3398590000	-1.8606430000	-0.8488320000
H	0.8804700000	1.8699720000	1.1739850000
H	4.2057850000	1.6913380000	0.4756270000
H	1.2061580000	1.8382960000	-1.7541460000
H	1.7748660000	3.3581930000	-2.4341120000
H	0.7777900000	3.3619470000	-0.9981530000
H	5.5057480000	3.0941140000	-0.2247850000
H	6.7398100000	5.1933500000	-0.0022820000
H	5.5719410000	7.3640770000	-0.2413260000
H	3.1415150000	7.3934680000	-0.7140860000
H	1.9006740000	5.2977510000	-0.9322130000

Intermediate IV

C	-1.7116920000	-0.4055800000	0.7222960000
C	-1.6295950000	-1.0341210000	-0.5141630000
C	-0.4244570000	-1.0616290000	-1.1992710000
C	0.6937070000	-0.4463730000	-0.6610030000
C	0.6233470000	0.1876460000	0.5790860000
C	-0.5912820000	0.1905350000	1.2687710000
C	1.7846440000	0.8698710000	1.2074030000
C	2.9010440000	1.4047700000	0.3416600000
O	1.8112560000	1.0523530000	2.4109970000
C	2.5601800000	2.7850610000	-0.2900020000
C	1.5903430000	2.6351100000	-1.4598470000
C	2.0338620000	3.7201760000	0.7946100000
C	1.9673780000	1.9067590000	-2.5923900000
C	1.1132720000	1.7649540000	-3.6728770000
C	-0.1506570000	2.3359490000	-3.6458480000
C	-0.5430600000	3.0553700000	-2.5301020000
C	0.3200720000	3.2086600000	-1.4535380000

C	3.9151090000	3.3845380000	-0.7908160000
O	4.7407540000	2.4468260000	-1.4143920000
C	3.7020630000	4.5602410000	-1.7055770000
O	4.2614810000	4.6375600000	-2.7711880000
H	-2.6524120000	-0.3828300000	1.2573940000
H	-2.5061820000	-1.5037600000	-0.9421960000
H	-0.3557700000	-1.5570190000	-2.1593110000
H	1.6284450000	-0.4735150000	-1.2047810000
H	-0.6326280000	0.6799590000	2.2337240000
H	3.1779930000	0.7117590000	-0.4511670000
H	3.7707440000	1.5288430000	0.9874770000
H	1.0841180000	3.3797240000	1.2032090000
H	1.8815250000	4.7342650000	0.4258890000
H	2.7379840000	3.7665490000	1.6246540000
H	2.9429650000	1.4385140000	-2.6198160000
H	1.4355280000	1.1990860000	-4.5380710000
H	-0.8228880000	2.2187750000	-4.4860650000
H	-1.5273210000	3.5047470000	-2.4909350000
H	-0.0158050000	3.7780220000	-0.5974430000
H	4.4204980000	3.7820730000	0.1065500000
H	4.9951550000	2.8497100000	-2.2617330000
H	3.0220220000	5.3581380000	-1.3497100000

Intermediate VII

C	-1.6482050000	-0.8442500000	0.4169040000
C	-1.2330680000	-1.5423150000	-0.7108530000
C	0.0414110000	-1.3421870000	-1.2176200000
C	0.8992230000	-0.4415990000	-0.6067720000
C	0.4937030000	0.2678920000	0.5243960000
C	-0.7911880000	0.0483990000	1.0293340000
C	1.3517320000	1.2634030000	1.2229560000
C	2.6473160000	1.7261700000	0.5991960000
O	0.9991660000	1.7331730000	2.2911080000
C	2.4927950000	3.0577190000	-0.1805100000
C	1.4883530000	2.8724520000	-1.3202880000
C	2.1048600000	4.1966250000	0.7631290000
C	1.8689130000	2.1995350000	-2.4816790000
C	0.9585040000	1.9566940000	-3.4970880000
C	-0.3574460000	2.3741180000	-3.3664190000
C	-0.7485580000	3.0430040000	-2.2178250000

C	0.1674280000	3.2974140000	-1.2070230000
C	3.8438960000	3.3888700000	-0.8165400000
O	4.7557580000	2.5926650000	-0.8846310000
C	4.0428910000	4.7637300000	-1.4070160000
O	5.2227190000	4.8359570000	-2.1419530000
H	-2.6432520000	-0.9986970000	0.8141800000
H	-1.9037110000	-2.2425180000	-1.1926790000
H	0.3695590000	-1.8872830000	-2.0933290000
H	1.8898390000	-0.2997250000	-1.0166580000
H	-1.0953020000	0.6026670000	1.9078150000
H	3.0768860000	0.9837550000	-0.0691520000
H	3.3616790000	1.8859340000	1.4074430000
H	1.2245840000	3.9425380000	1.3481670000
H	1.8945890000	5.1165580000	0.2190600000
H	2.9077240000	4.3929160000	1.4735510000
H	2.8897280000	1.8490870000	-2.5909770000
H	1.2787720000	1.4365190000	-4.3909050000
H	-1.0723850000	2.1809500000	-4.1556140000
H	-1.7734180000	3.3725530000	-2.1033290000
H	-0.1613720000	3.8177800000	-0.3167290000
H	4.0469700000	5.4856250000	-0.5769720000
H	3.1794150000	5.0250800000	-2.0303600000
H	5.6865300000	4.0035460000	-1.9554730000

Intermediate VIII

C	-0.8838880000	1.2416190000	-0.0193100000
C	-1.5343850000	0.1246400000	-0.5171460000
C	-0.8025350000	-1.0123070000	-0.8325070000
C	0.5684530000	-1.0279090000	-0.6450510000
C	1.2338780000	0.0889640000	-0.1388380000
C	0.4914840000	1.2255210000	0.1683550000
C	2.7322380000	0.0430140000	0.0004170000
C	3.2755410000	-1.1533050000	0.7870970000
O	3.3617510000	-0.0703980000	-1.2919920000
C	4.7480250000	-0.8155520000	1.1342470000
C	5.0573120000	-1.0792030000	2.6051930000
C	5.7464910000	-1.5798000000	0.2650670000
C	5.7727580000	-0.1882700000	3.4014530000
C	6.0534410000	-0.4902980000	4.7275310000
C	5.6334930000	-1.6889250000	5.2789380000

C	4.9274900000	-2.5893160000	4.4935980000
C	4.6428190000	-2.2857680000	3.1732620000
C	4.8427960000	0.6840170000	0.8018500000
O	5.8588110000	1.3127510000	0.6703150000
C	3.4202330000	1.2252660000	0.6831260000
O	2.9370670000	1.5335760000	1.9832890000
H	-1.4468180000	2.1330750000	0.2267260000
H	-2.6070870000	0.1383980000	-0.6620160000
H	-1.3023510000	-1.8877160000	-1.2275620000
H	1.1384200000	-1.9115720000	-0.9069570000
H	0.9850030000	2.1009670000	0.5689310000
H	3.1832480000	-2.0687690000	0.2036480000
H	2.6759930000	-1.2913760000	1.6882750000
H	3.0862620000	0.6879350000	-1.8224410000
H	5.7011310000	-2.6465040000	0.4842950000
H	6.7613780000	-1.2354910000	0.4587910000
H	5.5287610000	-1.4339040000	-0.7920740000
H	6.1195900000	0.7492110000	2.9867730000
H	6.6052480000	0.2207800000	5.3292090000
H	5.8535250000	-1.9221950000	6.3126960000
H	4.5965310000	-3.5322910000	4.9103140000
H	4.0925490000	-2.9985230000	2.5692800000
H	3.3724750000	2.1480260000	0.1044270000
H	3.0328660000	0.7540340000	2.5477880000

Table S3. Energy data for calculated complexes, transition states, substrates and products based on standard state corrections using ethylene glycol as an implicit solvent.

**Structure**	**$E^{\omega B97M-V}{}_{Ethylene\ glycol}$**	**$G^{o\omega B97M-V}{}_{(Ethylene\ glycol,\ 403.15K)}$**	**Imaginary frequency**	**G T = 403.15K**
Energy Unit	Hartree	Hartree	cm^{-1}	kcal
**Mn-5**	-3598.612556	-3598.132458	-	-2257825.555
**Mn-6**	-3542.012724	-3541.570818	-	-2222333.126
**Mn-7**	-3543.186982	-3542.72506	-	-2223057.413
**$TS_{6,7}$-PE**	-3928.088809	-3927.499697	-454.0711	-2464503.497
**$TS_{6,7}$-EG**	-3772.26791	-3771.750433	-403.3695	-2366770.834
**$TS_{6,7}$-IV**	-4464.437674	-4463.715502	-367.1056	-2800978.915
**$TS_{6,7}$-VI**	-4465.648112	-4464.905509	-518.4346	-2801725.644
**$TS_{7,6}$**	-3543.172425	-3542.716149	-1366.9493	-2223051.821

**$TS_{7,6}$-IV**	-4465.655854	-4464.907418	-355.2428	-2801726.842
**$TS_{7,6}$-V**	-4464.447744	-4463.723984	-70.5610	-2800984.238
**H_2**	-1.160427309	-1.167436309	-	-730.0037001
**NH_3**	-56.55663025	-56.54970325	-	-35482.3762
**H_2O**	-76.44438187	-76.44951287	-	-47969.50674
**PE**	-386.0911183	-385.9790473	-	-242199.2896
**Intermediate I**	-384.8963049	-384.8067299	-	-241463.6604
**EG**	-230.2735846	-230.2278276	-	-144465.3993
**Intermediate III**	-229.0667101	-229.0451861	-	-143723.2917
**Intermediate IX**	-769.8074019	-769.5931089	-	-482917.1133
**Intermediate II**	-693.3405024	-693.1518854		-434950.2455
**Intermediate IV**	-922.4503745	-922.2053595	-	-578681.3005
**Intermediate VII**	-922.459033	-922.213289	-	-578686.2763
**Intermediate VIII**	-922.4605197	-922.2119537	-	-578685.4384
**Product 1**	-846.0242128	-845.8009088	-	-530737.5077
**Intermediate V**	-921.2341002	-921.0123542	-	-577932.6896
**Intermediate VI**	-923.6515365	-923.3828375	-	-579420.168

Cartesian coordinates for calculated structures

Mn-5

Mn	11.3751450000	13.3189240000	6.4922620000
P	11.4413710000	13.8248780000	8.6926650000
P	11.8424460000	12.5702540000	4.4113470000
O	8.7899000000	14.5846690000	5.9070180000
O	10.0893360000	10.7520300000	7.0558970000
N	13.2255080000	12.4003210000	6.9406620000
N	12.5573000000	15.0923100000	6.1111810000
H	12.7317450000	15.2885370000	5.1304510000
H	13.4674850000	14.9758520000	6.5466950000
H	12.1578170000	15.9403580000	6.5013320000
C	12.7746980000	12.7662480000	9.2831170000
C	13.0666690000	12.5319720000	10.6245460000
H	12.4323540000	12.9604800000	11.3915910000
C	14.1485290000	11.7507180000	10.9855610000
H	14.3635080000	11.5536650000	12.0275840000
C	14.9691130000	11.2377460000	9.9835850000
H	15.8387050000	10.6465790000	10.2519410000

C	14.7223690000	11.4727180000	8.6443150000
C	13.5801680000	12.2137850000	8.2586600000
C	15.6723060000	11.0220830000	7.5802270000
H	16.1943120000	10.1113040000	7.8797150000
H	16.4622610000	11.7796090000	7.4692830000
C	14.9903060000	10.8420100000	6.2614210000
C	15.4917800000	9.9908030000	5.2959820000
H	16.3672180000	9.3966620000	5.5374820000
C	14.9160450000	9.8853100000	4.0307200000
H	15.3275200000	9.2085470000	3.2935460000
C	13.8170290000	10.6678850000	3.7352870000
H	13.3518130000	10.6069330000	2.7572370000
C	13.2773680000	11.5285580000	4.6913270000
C	13.8328580000	11.6114560000	5.9897570000
C	11.9578260000	15.5280040000	9.0896180000
C	11.0210420000	16.5540770000	8.9337860000
H	9.9960480000	16.3109500000	8.6700600000
C	11.3906630000	17.8769550000	9.1077700000
H	10.6536670000	18.6614290000	8.9889260000
C	12.7036480000	18.1965430000	9.4305390000
H	12.9928020000	19.2310970000	9.5655490000
C	13.6419490000	17.1867450000	9.5763320000
H	14.6671050000	17.4299010000	9.8267340000
C	13.2735870000	15.8589970000	9.4046150000
H	14.0158100000	15.0766150000	9.5165320000
C	10.0039090000	13.6205990000	9.7954790000
C	8.8429130000	12.9937840000	9.3462170000
H	8.7768020000	12.6389260000	8.3264270000
C	7.7536290000	12.8352530000	10.1909230000
H	6.8583930000	12.3492400000	9.8240030000
C	7.8074520000	13.3049780000	11.4936060000
H	6.9566870000	13.1815580000	12.1518970000
C	8.9504860000	13.9486750000	11.9473130000
H	8.9942800000	14.3319590000	12.9590490000
C	10.0365190000	14.1123490000	11.1039330000
H	10.9133390000	14.6399430000	11.4612850000
C	10.6011500000	11.5194870000	3.5863520000
C	9.6338920000	12.0657660000	2.7427390000
H	9.6588100000	13.1196860000	2.4940280000
C	8.6334110000	11.2689760000	2.2076930000
H	7.8943000000	11.7090430000	1.5498980000
C	8.5800080000	9.9164130000	2.5100010000

H	7.7996940000	9.2948920000	2.0892440000
C	9.5321460000	9.3646240000	3.3543600000
H	9.4988940000	8.3097750000	3.5969940000
C	10.5333230000	10.1587920000	3.8912710000
H	11.2698870000	9.7171190000	4.5520700000
C	12.2924430000	13.8013270000	3.1361180000
C	13.4564330000	13.7143430000	2.3737570000
H	14.1339130000	12.8811300000	2.5093250000
C	13.7643200000	14.6952900000	1.4425530000
H	14.6732260000	14.6121130000	0.8598000000
C	12.9171860000	15.7773270000	1.2591910000
H	13.1603570000	16.5417790000	0.5321110000
C	11.7627900000	15.8837300000	2.0214400000
H	11.1021320000	16.7322030000	1.8952630000
C	11.4575530000	14.9098740000	2.9588680000
H	10.5672400000	15.0157500000	3.5697090000
C	9.8328240000	14.1045830000	6.1273840000
C	10.5808070000	11.7870670000	6.8331190000

Mn-6

Mn	12.3353440000	8.9999890000	11.1134610000
P	10.9971590000	10.5720160000	10.2607360000
P	12.7457070000	6.9282290000	11.8548890000
O	13.0708350000	10.4674390000	13.5499050000
C	10.5181860000	9.9137980000	8.6647430000
C	9.8389570000	10.6146130000	7.6763110000
H	9.5797820000	11.6547250000	7.8384390000
C	9.4999350000	9.9872680000	6.4905660000
H	8.9740360000	10.5244900000	5.7124330000
C	9.8477040000	8.6531520000	6.3163390000
C	12.0228090000	4.5330460000	8.0795060000
H	11.7953540000	3.9901950000	7.1676640000
C	12.7042820000	3.8900600000	9.1052780000
H	13.0098200000	2.8574770000	8.9997130000
C	12.9765780000	4.5926710000	10.2669720000
H	13.4904360000	4.1113820000	11.0916940000
C	12.5856180000	5.9193930000	10.3834980000
C	14.1370170000	6.3364360000	12.8429740000
C	11.2530120000	6.5682170000	12.8422560000
C	10.2513780000	5.7008870000	12.4089800000

H	10.3706760000	5.1559860000	11.4798420000
C	9.1005050000	5.5262550000	13.1652350000
H	8.3309630000	4.8476500000	12.8188130000
C	8.9360090000	6.2137460000	14.3577010000
H	8.0354480000	6.0782300000	14.9435710000
C	9.9252680000	7.0854630000	14.7943380000
H	9.8002280000	7.6304880000	15.7217930000
C	11.0724080000	7.2673970000	14.0406790000
H	11.8412240000	7.9489840000	14.3889240000
C	12.7638290000	9.8756700000	12.5917970000
H	9.5883580000	8.1508180000	5.3899060000
O	14.9076150000	9.6992070000	9.9573350000
C	10.5304600000	7.9299240000	7.2819790000
C	10.8941060000	8.5608830000	8.4887370000
C	10.8931000000	6.4992390000	7.0283670000
H	9.9895650000	5.9237630000	6.7976010000
H	11.4970100000	6.4286820000	6.1166890000
C	11.6229650000	5.8582900000	8.1676390000
C	11.9204710000	6.5919990000	9.3324080000
C	13.8762080000	9.4205270000	10.4295080000
C	15.3466750000	7.0279150000	12.7908040000
H	15.4307280000	7.9192420000	12.1795020000
C	16.4365530000	6.5829530000	13.5224550000
H	17.3721010000	7.1258460000	13.4778040000
C	16.3248290000	5.4508090000	14.3162700000
H	17.1753250000	5.1072820000	14.8915750000
C	15.1212260000	4.7625080000	14.3805830000
H	15.0311470000	3.8819420000	15.0040180000
C	14.0299530000	5.2020520000	13.6489720000
H	13.0891240000	4.6660150000	13.7052130000
C	9.6013130000	10.2753490000	11.3938870000
C	9.5219600000	10.9904250000	12.5925480000
H	10.2125860000	11.8052050000	12.7814170000
C	8.5681640000	10.6635520000	13.5416890000
H	8.5113050000	11.2317660000	14.4617160000
C	7.6936120000	9.6083630000	13.3180100000
H	6.9543660000	9.3483860000	14.0653410000
C	7.7749640000	8.8834680000	12.1382180000
H	7.0971080000	8.0580870000	11.9591670000
C	8.7228050000	9.2119160000	11.1805890000
H	8.7770360000	8.6403220000	10.2607750000
C	11.2117810000	12.3579420000	10.1494940000

C	12.5055220000	12.8752290000	10.0946220000
H	13.3552870000	12.2038170000	10.1443290000
C	12.7054150000	14.2420240000	9.9796880000
H	13.7126460000	14.6364890000	9.9374380000
C	11.6173180000	15.1005000000	9.9273780000
H	11.7746860000	16.1683340000	9.8420180000
C	10.3265360000	14.5934030000	9.9909850000
H	9.4769520000	15.2634800000	9.9552510000
C	10.1212280000	13.2280790000	10.1025650000
H	9.1125290000	12.8350310000	10.1582390000
N	11.5871510000	7.9267800000	9.5076480000

Mn-7

Mn	11.3971570000	8.6367440000	11.5344690000
P	10.3664650000	10.3261710000	10.5019980000
P	12.2137910000	6.6347930000	12.0995510000
O	11.9382250000	10.0028120000	14.0625780000
C	10.2569010000	9.7425580000	8.7836150000
C	10.0146420000	10.5649770000	7.6856430000
H	9.8546430000	11.6258830000	7.8365630000
C	10.0158620000	10.0389580000	6.4049650000
H	9.8446050000	10.6846240000	5.5533980000
C	10.2477920000	8.6868500000	6.2164910000
C	12.2272120000	4.5924140000	7.9854970000
H	12.2271960000	4.1459690000	6.9980000000
C	12.9312930000	3.9946830000	9.0180030000
H	13.4860760000	3.0825750000	8.8394240000
C	12.9296680000	4.5715300000	10.2764140000
H	13.4860120000	4.1134280000	11.0859180000
C	12.2417860000	5.7608070000	10.5011990000
C	13.9346780000	6.6477640000	12.6904730000
C	11.4237500000	5.4200040000	13.2207070000
C	12.1363150000	4.3324590000	13.7319600000
H	13.1912560000	4.2218370000	13.5096310000
C	11.5060880000	3.3913850000	14.5295080000
H	12.0714520000	2.5545600000	14.9204020000
C	10.1567100000	3.5215540000	14.8287290000
H	9.6668470000	2.7869500000	15.4555710000
C	9.4408460000	4.5969460000	14.3262300000
H	8.3890130000	4.7069740000	14.5591530000

C	10.0711710000	5.5405470000	13.5282120000
H	9.5145590000	6.3862220000	13.1397890000
C	11.7182820000	9.4542900000	13.0531580000
H	10.2511250000	8.2704600000	5.2159580000
O	14.0438140000	9.4415240000	10.5103470000
N	10.7637110000	7.5449730000	9.7256360000
C	10.4774020000	7.8391560000	7.2943020000
C	10.4952040000	8.3827300000	8.5752810000
C	10.6775240000	6.3707840000	7.1048960000
H	9.6957780000	5.8813540000	7.0838530000
H	11.1206120000	6.1666820000	6.1297560000
C	11.4973560000	5.7571780000	8.1926130000
C	11.5177930000	6.3380320000	9.4573670000
C	12.9866030000	9.1067680000	10.8789950000
H	9.8748780000	7.2809710000	10.1516740000
H	9.9166220000	8.2183490000	12.0988220000
C	15.0237740000	6.5951930000	11.8230900000
H	14.8655340000	6.4411440000	10.7621400000
C	16.3153560000	6.7475100000	12.3065400000
H	17.1521890000	6.6980820000	11.6210070000
C	16.5348180000	6.9624980000	13.6586020000
H	17.5435740000	7.0798110000	14.0338820000
C	15.4551020000	7.0314880000	14.5287630000
H	15.6176190000	7.2054010000	15.5851240000
C	14.1650250000	6.8787990000	14.0488410000
H	13.3269570000	6.9385670000	14.7354350000
C	8.6827420000	10.8334410000	10.9977470000
C	8.3862400000	10.8636340000	12.3616370000
H	9.1401610000	10.5606450000	13.0799110000
C	7.1391210000	11.2741480000	12.8043590000
H	6.9271600000	11.2982790000	13.8659330000
C	6.1632790000	11.6416730000	11.8889860000
H	5.1846850000	11.9519910000	12.2331490000
C	6.4435800000	11.6035880000	10.5315090000
H	5.6841470000	11.8821720000	9.8115950000
C	7.6972540000	11.2090920000	10.0873190000
H	7.8978830000	11.1863350000	9.0237080000
C	11.2445250000	11.9208000000	10.3739620000
C	12.3200110000	12.0519360000	9.4927950000
H	12.5741760000	11.2371690000	8.8245850000
C	13.0657010000	13.2187240000	9.4602940000
H	13.8908460000	13.3074730000	8.7646240000

C	12.7579090000	14.2684180000	10.3147800000
H	13.3418890000	15.1797210000	10.2890490000
C	11.6993530000	14.1438010000	11.2012060000
H	11.4524680000	14.9574190000	11.8717730000
C	10.9465030000	12.9788950000	11.2314770000
H	10.1199350000	12.8980390000	11.9278820000

$TS_{6,7}$-PE

Mn	11.2392810000	8.6464910000	11.6051050000
P	10.2720660000	10.3248460000	10.4591220000
P	12.2326790000	6.7224490000	12.2191280000
O	11.4152130000	10.0441560000	14.1724960000
C	10.2483040000	9.6838850000	8.7634380000
C	9.9992500000	10.4663570000	7.6375760000
H	9.7806500000	11.5208100000	7.7579720000
C	10.0608490000	9.9076580000	6.3731920000
H	9.8826640000	10.5207010000	5.4992980000
C	10.3533770000	8.5611740000	6.2302210000
C	12.3753800000	4.5722210000	8.1730420000
H	12.4194720000	4.1074850000	7.1950110000
C	13.0783440000	4.0258420000	9.2334210000
H	13.6783670000	3.1369450000	9.0883950000
C	13.0239930000	4.6324200000	10.4756630000
H	13.5978980000	4.2307610000	11.3022160000
C	12.2664480000	5.7871870000	10.6639650000
C	13.9930730000	6.8255430000	12.7030090000
C	11.5241380000	5.6520070000	13.5144330000
C	11.3317360000	4.2803260000	13.3664350000
H	11.6293600000	3.7806420000	12.4536490000
C	10.7250190000	3.5442830000	14.3734390000
H	10.5708200000	2.4809580000	14.2387070000
C	10.3119960000	4.1645050000	15.5432080000
H	9.8350000000	3.5873470000	16.3251990000
C	10.5056410000	5.5289580000	15.7039820000
H	10.1818110000	6.0234480000	16.6113500000
C	11.0995000000	6.2685400000	14.6938870000
H	11.2205760000	7.3386840000	14.8179370000
C	11.3493040000	9.4828330000	13.1507060000
H	10.3935950000	8.1162470000	5.2428200000
O	13.8702850000	9.7003950000	10.8402510000

C	10.5844030000	7.7532990000	7.3374860000
C	10.5544630000	8.3327770000	8.6021100000
C	10.8112890000	6.2835060000	7.2010960000
H	9.8354780000	5.7825940000	7.1673490000
H	11.2865310000	6.0526420000	6.2474000000
C	11.6027380000	5.7164600000	8.3335940000
C	11.5591040000	6.3254760000	9.5855150000
C	12.8221320000	9.2634160000	11.1058500000
C	14.9859680000	6.9226990000	11.7266510000
H	14.7221910000	6.8620600000	10.6772890000
C	16.3121270000	7.1037520000	12.0848430000
H	17.0694730000	7.1703740000	11.3137180000
C	16.6677650000	7.2012730000	13.4222740000
H	17.7043900000	7.3421850000	13.7010000000
C	15.6876690000	7.1184340000	14.3995750000
H	15.9543830000	7.1949120000	15.4462430000
C	14.3601890000	6.9327620000	14.0440390000
H	13.6099380000	6.8661430000	14.8224430000
C	8.5997590000	10.9397620000	10.8402540000
C	8.3427720000	11.3491720000	12.1527250000
H	9.0999790000	11.2190950000	12.9182340000
C	7.1288060000	11.9266290000	12.4854490000
H	6.9515500000	12.2535140000	13.5025020000
C	6.1378400000	12.0647760000	11.5238290000
H	5.1853800000	12.5086730000	11.7856130000
C	6.3616630000	11.6080510000	10.2342940000
H	5.5835130000	11.6889430000	9.4855460000
C	7.5862420000	11.0523310000	9.8909140000
H	7.7469400000	10.7073060000	8.8773900000
C	11.2536850000	11.8658950000	10.3249350000
C	12.2833800000	11.9547910000	9.3859200000
H	12.4491880000	11.1461020000	8.6844550000
C	13.1088110000	13.0670330000	9.3434360000
H	13.8975270000	13.1179060000	8.6031410000
C	12.9280620000	14.1067900000	10.2433280000
H	13.5732410000	14.9755540000	10.2094400000
C	11.9166820000	14.0242650000	11.1879020000
H	11.7671060000	14.8283130000	11.8975910000
C	11.0873350000	12.9136990000	11.2302930000
H	10.3048150000	12.8739170000	11.9768000000
H	9.6596100000	8.0453580000	12.0845170000
N	10.7674300000	7.5250050000	9.7915890000

H	9.8081680000	7.2418300000	10.0675440000
C	5.2893660000	9.1932020000	13.1932700000
C	5.6975060000	9.4060800000	14.5005680000
C	6.9633500000	8.9952830000	14.9039330000
C	7.8055260000	8.3610520000	14.0073550000
C	7.4029130000	8.1464400000	12.6898770000
C	6.1416690000	8.5751540000	12.2892990000
C	8.2326730000	7.3621180000	11.7122280000
C	8.5734500000	5.9661170000	12.1757840000
O	8.0556490000	7.5379750000	10.4737920000
H	4.3065620000	9.5160010000	12.8714730000
H	5.0359660000	9.8954640000	15.2045670000
H	7.2909320000	9.1682660000	15.9217020000
H	8.7955400000	8.0434400000	14.3197090000
H	5.8295550000	8.4039790000	11.2657440000
H	7.6687820000	5.3637190000	12.0514530000
H	8.8639460000	5.9170480000	13.2216270000
H	9.3524340000	5.5227160000	11.5549020000

TS$_{6,7}$-EG

Mn	11.2312290000	8.8278960000	11.4205090000
P	10.4835800000	10.4570680000	10.0663400000
P	11.9093740000	6.8726860000	12.2923660000
O	11.3414950000	10.4409220000	13.8606330000
C	10.6765290000	9.7173070000	8.4207010000
C	10.6523540000	10.4390150000	7.2296760000
H	10.4875570000	11.5096440000	7.2569350000
C	10.8677960000	9.7977770000	6.0217650000
H	10.8661260000	10.3635170000	5.0991730000
C	11.0882130000	8.4308250000	5.9989030000
C	12.4741740000	4.4333670000	8.4527630000
H	12.6310480000	3.9009660000	7.5219070000
C	12.9445040000	3.9041080000	9.6423920000
H	13.4744310000	2.9605070000	9.6459130000
C	12.7509930000	4.5964480000	10.8252380000
H	13.1461180000	4.2051880000	11.7551430000
C	12.0860230000	5.8203550000	10.8229160000
C	13.5737200000	6.8259330000	13.0471190000
C	10.8833610000	5.9933040000	13.5153200000
C	10.5932420000	4.6326670000	13.4486660000

H	10.9783540000	4.0307570000	12.6355540000
C	9.7805500000	4.0411440000	14.4044320000
H	9.5519590000	2.9852070000	14.3315140000
C	9.2625940000	4.7950310000	15.4470040000
H	8.6297910000	4.3291780000	16.1918670000
C	9.5513880000	6.1496890000	15.5274410000
H	9.1461680000	6.7471850000	16.3342930000
C	10.3460860000	6.7470740000	14.5622540000
H	10.5428190000	7.8121030000	14.6148410000
C	11.2867900000	9.7881370000	12.8936770000
H	11.2495600000	7.9226500000	5.0553480000
O	14.0098250000	9.5966300000	10.8576230000
C	11.0947760000	7.6819000000	7.1705470000
C	10.9114550000	8.3414350000	8.3812980000
C	11.2465520000	6.1961070000	7.1455710000
H	10.2616170000	5.7481580000	6.9623740000
H	11.8624510000	5.8869530000	6.3004550000
C	11.7941300000	5.6459620000	8.4218790000
C	11.6104300000	6.3381050000	9.6154660000
C	12.9058920000	9.2704580000	11.0473890000
C	14.7038270000	6.8229860000	12.2265130000
H	14.5906560000	6.7714430000	11.1497670000
C	15.9741930000	6.8847410000	12.7749570000
H	16.8390550000	6.8737720000	12.1233890000
C	16.1378920000	6.9606450000	14.1509070000
H	17.1312660000	7.0082230000	14.5787970000
C	15.0224860000	6.9748690000	14.9739640000
H	15.1398800000	7.0335370000	16.0488190000
C	13.7490480000	6.9084360000	14.4277630000
H	12.8900950000	6.9151470000	15.0877820000
C	8.7683970000	11.0590670000	10.2021410000
C	8.2902560000	11.3698350000	11.4771530000
H	8.9350180000	11.2407890000	12.3400210000
C	6.9934080000	11.8266730000	11.6522800000
H	6.6393660000	12.0686250000	12.6464690000
C	6.1488660000	11.9537530000	10.5592160000
H	5.1313400000	12.2976040000	10.6959700000
C	6.6088880000	11.6288290000	9.2914500000
H	5.9507100000	11.7150900000	8.4359460000
C	7.9119360000	11.1897670000	9.1111440000
H	8.2521180000	10.9312040000	8.1162020000
C	11.4666680000	11.9931550000	9.9895520000

C	12.6618050000	12.0197180000	9.2679860000
H	12.9633730000	11.1559450000	8.6874310000
C	13.4701440000	13.1447530000	9.2839280000
H	14.3894250000	13.1503440000	8.7116910000
C	13.1045340000	14.2573370000	10.0279530000
H	13.7367140000	15.1361820000	10.0393620000
C	11.9250670000	14.2372470000	10.7563200000
H	11.6309390000	15.1004860000	11.3401230000
C	11.1112500000	13.1142980000	10.7387850000
H	10.1908460000	13.1195870000	11.3098460000
H	9.5772620000	8.3867970000	11.7502460000
N	10.9038290000	7.6048660000	9.6319500000
H	9.9091840000	7.4091180000	9.8189840000
H	7.6224690000	8.7982040000	11.6744580000
C	8.0439390000	7.9272470000	11.1477160000
O	8.1123350000	7.9062070000	9.9013530000
C	7.9231180000	6.6132200000	11.8684640000
O	8.5773370000	5.5814260000	11.1635090000
H	6.8419800000	6.4149770000	11.9482180000
H	8.3171880000	6.6525880000	12.8821560000
H	8.4057460000	5.7532780000	10.2238680000

$TS_{6,7}$-IV

Mn	11.1140710000	8.6065640000	11.5529610000
P	10.2714430000	10.3747050000	10.3139090000
P	12.2166610000	6.7358850000	12.1404230000
O	11.2662730000	9.8643270000	14.1946550000
C	10.5041530000	9.7364110000	8.6319070000
C	10.3564150000	10.5187380000	7.4862430000
H	10.1649900000	11.5802510000	7.5854210000
C	10.4388010000	9.9441110000	6.2316100000
H	10.3469800000	10.5595220000	5.3461170000
C	10.6174500000	8.5749370000	6.1114310000
C	12.4124720000	4.5915960000	8.0919980000
H	12.4545600000	4.1203890000	7.1172650000
C	13.1565730000	4.0836670000	9.1440530000
H	13.7867540000	3.2164560000	8.9959060000
C	13.1037260000	4.6988360000	10.3812410000
H	13.7058230000	4.3244810000	11.2001040000
C	12.3040320000	5.8249650000	10.5771420000

C	13.9609410000	6.9523880000	12.6415050000
C	11.5732370000	5.5928730000	13.4079540000
C	11.4846850000	4.2124990000	13.2345550000
H	11.7970910000	3.7539670000	12.3057570000
C	10.9710110000	3.4100960000	14.2422940000
H	10.9024710000	2.3404090000	14.0888660000
C	10.5353130000	3.9720540000	15.4327560000
H	10.1280030000	3.3428700000	16.2140650000
C	10.6152690000	5.3448150000	15.6144320000
H	10.2687430000	5.7965600000	16.5359720000
C	11.1277900000	6.1487350000	14.6092090000
H	11.1721220000	7.2212910000	14.7593050000
C	11.2008870000	9.3755390000	13.1387390000
H	10.6594040000	8.1147360000	5.1314290000
O	13.7305780000	9.7489070000	10.8724390000
C	10.7128020000	7.7667010000	7.2359900000
C	10.6815900000	8.3625370000	8.4945720000
C	10.7996400000	6.2791530000	7.1413920000
H	9.7839510000	5.8666530000	7.1935100000
H	11.1972160000	5.9703570000	6.1755560000
C	11.6096510000	5.7122900000	8.2590160000
C	11.5672970000	6.3306970000	9.5064010000
C	12.6859640000	9.2853450000	11.0991790000
C	14.9757170000	7.0225150000	11.6866470000
H	14.7476460000	6.8711140000	10.6384720000
C	16.2802800000	7.2998020000	12.0639050000
H	17.0559020000	7.3447960000	11.3096180000
C	16.5899970000	7.5224990000	13.3970730000
H	17.6094050000	7.7391360000	13.6903030000
C	15.5856860000	7.4702310000	14.3522440000
H	15.8159590000	7.6476800000	15.3952420000
C	14.2807240000	7.1895380000	13.9785790000
H	13.5101240000	7.1554460000	14.7391800000
C	8.5887570000	11.0887650000	10.1657810000
C	8.1845660000	12.1845110000	10.9283550000
H	8.8919660000	12.7025060000	11.5623430000
C	6.8699020000	12.6233950000	10.8939610000
H	6.5768140000	13.4764550000	11.4931260000
C	5.9355690000	11.9747600000	10.1002220000
H	4.9078290000	12.3147190000	10.0795470000
C	6.3290750000	10.8931950000	9.3254070000
H	5.6108230000	10.3846880000	8.6941520000

C	7.6432050000	10.4544660000	9.3553510000
H	7.9271830000	9.6018140000	8.7513810000
C	11.3376370000	11.8406750000	10.5226470000
C	12.1955280000	12.3186370000	9.5352100000
H	12.2154480000	11.8590710000	8.5560810000
C	13.0618080000	13.3690590000	9.8036640000
H	13.7264560000	13.7227840000	9.0254350000
C	13.0845790000	13.9562230000	11.0585640000
H	13.7627350000	14.7745220000	11.2649170000
C	12.2433470000	13.4795730000	12.0541880000
H	12.2620120000	13.9212250000	13.0425300000
C	11.3862690000	12.4238810000	11.7923770000
H	10.7573970000	12.0412940000	12.5882870000
H	9.5994570000	7.9081290000	12.1334290000
N	10.7516630000	7.5291510000	9.6913510000
H	9.7833040000	7.2344050000	9.8841510000
C	7.2797580000	8.0142730000	12.9981920000
C	8.0358350000	7.3011990000	11.8381800000
C	8.6786010000	5.9444690000	12.0453470000
O	7.7302330000	7.5462840000	10.6566090000
O	8.4588820000	5.1724730000	12.9446420000
H	9.3284120000	5.6611870000	11.1859710000
C	7.5594410000	8.6335380000	15.4434740000
C	7.8958870000	7.6332780000	14.3739760000
O	8.3942710000	9.4469890000	15.8185550000
C	7.4220270000	9.5152390000	12.7882790000
C	6.1781300000	8.6786180000	15.9765140000
C	5.3760590000	7.5411890000	16.0764960000
C	4.0755850000	7.6427520000	16.5414070000
C	3.5573580000	8.8814990000	16.8920060000
C	4.3492860000	10.0192560000	16.7991040000
C	5.6551120000	9.9164420000	16.3561970000
C	5.8146420000	7.5759210000	12.8941510000
C	5.4666380000	6.2349540000	13.0714480000
C	4.1465470000	5.8205520000	13.0137760000
C	3.1342670000	6.7393590000	12.7755130000
C	3.4638580000	8.0707790000	12.5828440000
C	4.7890990000	8.4835550000	12.6331400000
H	7.5806700000	6.6347750000	14.6708500000
H	8.9809480000	7.6263950000	14.2690280000
H	6.8380860000	10.0806200000	13.5153510000
H	7.0738880000	9.7959540000	11.7999720000

H	8.4651980000	9.8168650000	12.8895840000
H	5.7742610000	6.5714130000	15.8084440000
H	3.4637530000	6.7534830000	16.6264150000
H	2.5350160000	8.9610620000	17.2397570000
H	3.9441890000	10.9859780000	17.0696930000
H	6.2819280000	10.7961460000	16.2718370000
H	6.2398430000	5.5061970000	13.2796790000
H	3.9082150000	4.7743370000	13.1616500000
H	2.1006940000	6.4190420000	12.7372020000
H	2.6873780000	8.8014720000	12.3919510000
H	5.0076270000	9.5316700000	12.4787240000

$TS_{6,7}$-VI

Mn	10.9546880000	8.7784400000	11.5359610000
P	10.3431010000	10.5116200000	10.1131100000
P	11.8719140000	6.8833260000	12.3249140000
O	10.9542970000	10.1753040000	14.1103580000
C	10.6370200000	9.7346640000	8.4998260000
C	10.6116800000	10.4391490000	7.2953180000
H	10.4727440000	11.5133440000	7.3081540000
C	10.7482630000	9.7740060000	6.0913010000
H	10.7505320000	10.3289010000	5.1621290000
C	10.8614150000	8.3926250000	6.0778100000
C	12.2905350000	4.4677140000	8.4541260000
H	12.3773380000	3.9248910000	7.5203730000
C	12.9059710000	3.9874310000	9.5988600000
H	13.4818370000	3.0718180000	9.5636150000
C	12.7883630000	4.6877470000	10.7857210000
H	13.2794610000	4.3275430000	11.6818370000
C	12.0591530000	5.8750910000	10.8337340000
C	13.5728150000	6.9989440000	12.9911330000
C	11.0248000000	5.8533320000	13.5686680000
C	10.7631670000	4.4943790000	13.4007690000
H	11.0742830000	3.9830140000	12.4993770000
C	10.0709300000	3.7878490000	14.3738060000
H	9.8642950000	2.7357260000	14.2228040000
C	9.6450030000	4.4217270000	15.5313220000
H	9.1078060000	3.8663800000	16.2901450000
C	9.9050200000	5.7731140000	15.7105400000
H	9.5756980000	6.2786410000	16.6097270000

C	10.5734130000	6.4867770000	14.7299400000
H	10.7374850000	7.5492180000	14.8673180000
C	10.9492530000	9.6324200000	13.0772760000
H	10.9463830000	7.8618280000	5.1369930000
O	13.6941070000	9.7083930000	11.0287350000
C	10.8366480000	7.6636040000	7.2584200000
C	10.7486750000	8.3469270000	8.4700770000
C	10.8542660000	6.1706460000	7.2701000000
H	9.8171400000	5.8099040000	7.2803890000
H	11.2969020000	5.7759970000	6.3563960000
C	11.5549050000	5.6452820000	8.4782160000
C	11.4566570000	6.3533200000	9.6729400000
C	12.6041580000	9.3262370000	11.1879960000
C	14.6666190000	6.9931780000	12.1239090000
H	14.5149520000	6.8530440000	11.0603500000
C	15.9525030000	7.1749240000	12.6071950000
H	16.7884280000	7.1608750000	11.9189920000
C	16.1678360000	7.3750450000	13.9626650000
H	17.1729760000	7.5156610000	14.3395700000
C	15.0874730000	7.3957540000	14.8314300000
H	15.2435110000	7.5539250000	15.8911900000
C	13.8001480000	7.2106530000	14.3507450000
H	12.9728830000	7.2274930000	15.0486650000
C	8.7557980000	11.3756420000	9.7861930000
C	8.4037250000	12.5359670000	10.4759440000
H	9.1003160000	12.9985350000	11.1627130000
C	7.1549500000	13.1121380000	10.3015290000
H	6.9027350000	14.0130790000	10.8468010000
C	6.2344100000	12.5399280000	9.4365040000
H	5.2583050000	12.9891510000	9.3037510000
C	6.5762320000	11.3917020000	8.7369690000
H	5.8685220000	10.9389890000	8.0534170000
C	7.8236260000	10.8132620000	8.9096590000
H	8.0640930000	9.9096190000	8.3639300000
C	11.5232030000	11.8948850000	10.2949200000
C	12.4610000000	12.2456910000	9.3271080000
H	12.4851870000	11.7331070000	8.3748120000
C	13.4016830000	13.2336820000	9.5830040000
H	14.1280780000	13.4865280000	8.8207780000
C	13.4194700000	13.8860130000	10.8052350000
H	14.1559130000	14.6548160000	11.0019080000
C	12.4958410000	13.5381420000	11.7809780000

H	12.5074900000	14.0320480000	12.7444130000
C	11.5646620000	12.5439310000	11.5324900000
H	10.8702090000	12.2607880000	12.3152340000
H	9.3485700000	8.1846920000	11.9593000000
N	10.7010880000	7.5983210000	9.7182760000
H	9.7121340000	7.3607040000	9.8846070000
C	7.0191190000	8.4526590000	12.6243830000
C	7.8314460000	7.6329900000	11.5630910000
O	7.6905020000	7.8956380000	10.3467570000
C	6.5976270000	8.5791960000	15.1725240000
C	7.4343610000	8.0435520000	14.0417010000
O	5.9778790000	9.6291890000	15.0889860000
C	7.2590670000	9.9396760000	12.3942480000
C	6.5773290000	7.8210220000	16.4515700000
C	6.7080470000	6.4329260000	16.5160350000
C	6.6341280000	5.7777310000	17.7349640000
C	6.4556500000	6.5012900000	18.9041140000
C	6.3298080000	7.8842190000	18.8524600000
C	6.3749830000	8.5359630000	17.6348150000
C	5.5545970000	8.0961090000	12.3438720000
C	4.8777940000	7.1123900000	13.0656360000
C	3.5587660000	6.7888140000	12.7803390000
C	2.8844440000	7.4359180000	11.7571970000
C	3.5457550000	8.4085060000	11.0223480000
C	4.8621530000	8.7324040000	11.3116230000
H	7.5077830000	6.9643240000	14.1471980000
H	8.4542750000	8.4046330000	14.2281080000
H	6.5128650000	10.5489350000	12.8976940000
H	7.2222140000	10.1658030000	11.3348980000
H	8.2435440000	10.2312110000	12.7629990000
H	6.8395760000	5.8504300000	15.6125290000
H	6.7165700000	4.6988800000	17.7704560000
H	6.4107750000	5.9883750000	19.8566200000
H	6.1934710000	8.4509800000	19.7646600000
H	6.2693130000	9.6124340000	17.5812620000
H	5.3745480000	6.5765960000	13.8657990000
H	3.0600670000	6.0250320000	13.3642060000
H	1.8549140000	7.1852870000	11.5347550000
H	3.0351270000	8.9232190000	10.2175230000
H	5.3542580000	9.4935270000	10.7198190000
C	8.0623000000	6.1657540000	11.8959850000
O	8.6897180000	5.4927590000	10.8290690000

H	7.0707090000	5.7325480000	12.1038560000
H	8.6727840000	6.0176430000	12.7805250000
H	8.2563580000	5.8176490000	10.0232880000

TS$_{7,6}$

Mn	11.2738570000	8.5579950000	11.5222390000
P	10.2882940000	10.2698010000	10.4486550000
P	12.2315850000	6.5994300000	12.0659640000
O	10.8450300000	9.5286760000	14.2506350000
C	10.1323380000	9.6541460000	8.7516980000
C	9.6129560000	10.4047750000	7.6995620000
H	9.2549080000	11.4107960000	7.8856940000
C	9.5792010000	9.8829980000	6.4199070000
H	9.1904960000	10.4708310000	5.5987310000
C	10.0568950000	8.5975560000	6.1969060000
C	12.2662040000	4.5042070000	7.9993030000
H	12.2750690000	4.0391190000	7.0194460000
C	12.8360190000	3.8421410000	9.0797180000
H	13.2915490000	2.8698060000	8.9454070000
C	12.8195130000	4.4411810000	10.3254610000
H	13.2689360000	3.9409310000	11.1754900000
C	12.2518830000	5.7022190000	10.4921090000
C	13.9618680000	6.7167800000	12.6128090000
C	11.4986060000	5.4735480000	13.3009370000
C	12.2243380000	4.4054120000	13.8333430000
H	13.2539260000	4.2463430000	13.5339440000
C	11.6407560000	3.5491560000	14.7526270000
H	12.2135670000	2.7235820000	15.1561490000
C	10.3293110000	3.7525360000	15.1598610000
H	9.8765680000	3.0851370000	15.8823950000
C	9.6044730000	4.8170180000	14.6465440000
H	8.5846750000	4.9866850000	14.9685400000
C	10.1861060000	5.6738810000	13.7237400000
H	9.6202270000	6.5096400000	13.3280410000
C	11.0263980000	9.1482670000	13.1637100000
H	10.0344060000	8.1830310000	5.1948240000
O	13.8990980000	9.8837110000	11.3773330000
N	11.0741230000	7.6064900000	9.6029030000
C	10.5525190000	7.8111540000	7.2251270000
C	10.5947780000	8.3462810000	8.5207790000

C	11.0042220000	6.4065320000	6.9633350000
H	10.1410590000	5.8051380000	6.6550030000
H	11.6725110000	6.3857580000	6.0977400000
C	11.6659740000	5.7461730000	8.1344040000
C	11.6629750000	6.3582110000	9.3964000000
C	12.8652640000	9.3517750000	11.4134100000
H	10.2158980000	7.5594770000	10.5345650000
H	9.7727130000	7.7580400000	11.5049250000
C	15.0333080000	6.5769410000	11.7349890000
H	14.8577640000	6.2794500000	10.7078560000
C	16.3293230000	6.8258140000	12.1653950000
H	17.1543760000	6.7092640000	11.4737560000
C	16.5674240000	7.2235040000	13.4716290000
H	17.5790110000	7.4178000000	13.8049530000
C	15.5029300000	7.3762170000	14.3509200000
H	15.6812090000	7.6921090000	15.3712080000
C	14.2092900000	7.1290480000	13.9244660000
H	13.3811370000	7.2634890000	14.6132860000
C	8.6465380000	10.8964400000	10.9363170000
C	7.8489250000	10.1395740000	11.7923120000
H	8.2216440000	9.1948830000	12.1715900000
C	6.5902490000	10.5882880000	12.1632900000
H	5.9824780000	9.9914870000	12.8317680000
C	6.1162390000	11.8006450000	11.6859780000
H	5.1350520000	12.1532740000	11.9781800000
C	6.9060680000	12.5668580000	10.8396810000
H	6.5431450000	13.5177220000	10.4700050000
C	8.1645790000	12.1214880000	10.4697990000
H	8.7796450000	12.7348190000	9.8214040000
C	11.2846810000	11.7874500000	10.3471450000
C	12.1287390000	12.0496080000	9.2713790000
H	12.1221120000	11.3976560000	8.4057250000
C	12.9889760000	13.1383550000	9.3040270000
H	13.6375640000	13.3335800000	8.4591140000
C	13.0198770000	13.9709740000	10.4118340000
H	13.6916650000	14.8197010000	10.4356080000
C	12.1890210000	13.7100710000	11.4940260000
H	12.2123220000	14.3531630000	12.3649230000
C	11.3306520000	12.6241260000	11.4647000000
H	10.6940130000	12.4177940000	12.3193220000

TS$_{7,6}$-IV

Mn	11.1735040000	8.8152390000	11.4019720000
P	10.4634580000	10.4148780000	9.9931010000
P	11.8167480000	6.8811690000	12.3338270000
O	11.1723530000	10.4588770000	13.8242030000
C	10.7346460000	9.6527600000	8.3676920000
C	10.7419050000	10.3560920000	7.1656960000
H	10.5502790000	11.4225950000	7.1696000000
C	11.0216090000	9.7026260000	5.9775090000
H	11.0432960000	10.2546760000	5.0469150000
C	11.2759150000	8.3414270000	5.9843700000
C	12.6117310000	4.3955410000	8.5614040000
H	12.8211440000	3.8533470000	7.6466770000
C	13.0299110000	3.8887400000	9.7795070000
H	13.5739850000	2.9541900000	9.8216980000
C	12.7691110000	4.5925410000	10.9426530000
H	13.1362850000	4.2218670000	11.8917250000
C	12.0847410000	5.8053190000	10.8937840000
C	13.4434580000	6.8998320000	13.1703420000
C	10.7935520000	6.0054610000	13.5697070000
C	10.7418590000	4.6173930000	13.6893150000
H	11.2201670000	3.9859930000	12.9517870000
C	10.0854350000	4.0282720000	14.7599740000
H	10.0574580000	2.9487370000	14.8400050000
C	9.4719080000	4.8140410000	15.7254620000
H	8.9675070000	4.3505100000	16.5644370000
C	9.4846630000	6.1954340000	15.5965380000
H	8.9951600000	6.8174330000	16.3357350000
C	10.1323790000	6.7865700000	14.5212990000
H	10.1385810000	7.8669030000	14.4269200000
C	11.1602760000	9.7949940000	12.8630410000
H	11.4872770000	7.8239970000	5.0558310000
O	13.9743560000	9.5758680000	10.9444440000
C	11.2514880000	7.6093730000	7.1667000000
C	11.0025330000	8.2818530000	8.3580150000
C	11.4345770000	6.1266950000	7.1701860000
H	10.4704910000	5.6564820000	6.9383360000
H	12.1019390000	5.8202470000	6.3639990000
C	11.9214860000	5.6000000000	8.4807610000
C	11.6689800000	6.3030570000	9.6548910000
C	12.8621280000	9.2579810000	11.0949050000

C	14.6233140000	6.8225110000	12.4284430000
H	14.5811790000	6.6616940000	11.3575730000
C	15.8557470000	6.9562540000	13.0479520000
H	16.7606810000	6.8867820000	12.4572440000
C	15.9307610000	7.1799540000	14.4149630000
H	16.8943560000	7.2836840000	14.8975000000
C	14.7642470000	7.2728950000	15.1588690000
H	14.8114570000	7.4511740000	16.2258870000
C	13.5298870000	7.1349820000	14.5425620000
H	12.6293000000	7.2113410000	15.1403120000
C	8.7320580000	10.9817670000	10.0637900000
C	8.2069310000	11.2975250000	11.3195420000
H	8.8324480000	11.2123310000	12.2017920000
C	6.8858630000	11.6954930000	11.4528980000
H	6.4946300000	11.9383170000	12.4329230000
C	6.0648250000	11.7596780000	10.3363790000
H	5.0281070000	12.0539750000	10.4406040000
C	6.5743480000	11.4373540000	9.0868480000
H	5.9359480000	11.4775550000	8.2131610000
C	7.9010530000	11.0571700000	8.9483340000
H	8.2793720000	10.7952720000	7.9679870000
C	11.4228910000	11.9656260000	9.9294460000
C	12.6534170000	11.9942220000	9.2698310000
H	12.9974960000	11.1215260000	8.7273740000
C	13.4416600000	13.1330420000	9.2977580000
H	14.3889360000	13.1402720000	8.7732110000
C	13.0200200000	14.2576590000	9.9926510000
H	13.6365600000	15.1473590000	10.0134330000
C	11.8044400000	14.2359870000	10.6588070000
H	11.4663320000	15.1089620000	11.2029710000
C	11.0104650000	13.0991520000	10.6287990000
H	10.0610720000	13.1036970000	11.1500480000
H	9.5151130000	8.3515590000	11.6571520000
N	10.9501520000	7.5620220000	9.6170460000
H	9.9520720000	7.3554230000	9.7561230000
H	7.5999690000	8.7966480000	11.4070190000
C	8.0048090000	7.8945570000	10.9276360000
O	8.1658360000	7.8473380000	9.6933590000
C	7.7852660000	6.5867810000	11.6617170000
O	8.3283300000	5.5312890000	10.8992840000
H	8.3043880000	6.5997580000	12.6198460000
H	8.2872360000	5.8252260000	9.9726350000

C	5.8897120000	7.3890320000	13.0899690000
H	6.7080210000	7.4199050000	13.8100290000
H	5.7747400000	8.3867070000	12.6637670000
C	6.2605450000	6.3854050000	11.9499720000
C	5.4300210000	6.6834070000	10.7093800000
H	5.5599850000	7.7177620000	10.3919670000
H	5.7153190000	6.0374920000	9.8775440000
H	4.3668010000	6.5422140000	10.8939820000
C	4.5976420000	6.9937800000	13.7491430000
O	3.5264250000	7.2400910000	13.2104900000
C	4.6428500000	6.2080450000	15.0065490000
C	5.6831880000	6.3233920000	15.9302820000
C	5.7026030000	5.5293730000	17.0642130000
C	4.7003490000	4.5919510000	17.2735960000
C	3.6619300000	4.4683470000	16.3592480000
C	3.6250050000	5.2817880000	15.2412460000
C	6.8420490000	4.4738390000	13.4933580000
H	6.4730980000	7.0458880000	15.7719900000
H	6.5045000000	5.6378340000	17.7837870000
H	4.7278440000	3.9571170000	18.1503150000
H	2.8829830000	3.7336250000	16.5191670000
H	2.8243300000	5.1893710000	14.5172030000
C	6.0535530000	4.9576570000	12.4494380000
C	6.6234320000	3.2236960000	14.0451590000
C	5.0464750000	4.1307150000	11.9592560000
H	7.2448530000	2.8825480000	14.8650090000
H	4.4131910000	4.4608630000	11.1469800000
C	4.8240210000	2.8728520000	12.5077580000
H	5.4273240000	1.4381130000	13.9864220000
C	5.6053310000	2.4154990000	13.5556260000
H	4.0304800000	2.2519440000	12.1104120000
H	7.6251210000	5.1010770000	13.9016430000

$TS_{7,6}$-V

Mn	11.3235360000	8.3975040000	11.7110580000
P	10.1724550000	10.1696420000	10.9122760000
P	12.4075270000	6.4335080000	11.9414450000
O	11.5494890000	9.2415090000	14.5043300000
C	9.9093730000	9.7346380000	9.1667980000
C	9.4404440000	10.6407360000	8.2179810000

H	9.1237190000	11.6280620000	8.5324490000
C	9.4220250000	10.3001870000	6.8770440000
H	9.0671930000	11.0102350000	6.1414100000
C	9.8801380000	9.0557780000	6.4782290000
C	12.5761440000	5.0982000000	7.5598420000
H	12.6055230000	4.8156370000	6.5139650000
C	13.3722710000	4.4361600000	8.4791340000
H	14.0318290000	3.6419900000	8.1543310000
C	13.3223290000	4.7947840000	9.8152510000
H	13.9449540000	4.2857360000	10.5416050000
C	12.4905090000	5.8310550000	10.2314540000
C	14.1525620000	6.4769100000	12.4764070000
C	11.6667660000	5.1097800000	12.9524500000
C	11.3467160000	3.8504870000	12.4501850000
H	11.6046000000	3.5873120000	11.4319710000
C	10.6633820000	2.9336210000	13.2378160000
H	10.4075620000	1.9643930000	12.8281860000
C	10.3074760000	3.2547480000	14.5389850000
H	9.7713750000	2.5381570000	15.1491660000
C	10.6303460000	4.5037500000	15.0532670000
H	10.3533830000	4.7660620000	16.0668040000
C	11.2908600000	5.4292280000	14.2618850000
H	11.4932550000	6.4189220000	14.6565970000
C	11.4632170000	8.9163770000	13.3867810000
H	9.8796400000	8.7881280000	5.4280560000
O	13.8857810000	9.7126330000	11.1393810000
C	10.3418260000	8.1266010000	7.4036820000
C	10.3557300000	8.4805900000	8.7495700000
C	10.7871690000	6.7662160000	6.9776870000
H	9.9028830000	6.1269570000	6.8625910000
H	11.2396270000	6.8063890000	5.9860810000
C	11.7152990000	6.1179790000	7.9518700000
C	11.6981010000	6.4924000000	9.2910590000
C	12.8631910000	9.1779670000	11.3090580000
C	15.1358140000	6.8784990000	11.5694700000
H	14.8689280000	7.0910110000	10.5409300000
C	16.4548970000	7.0087150000	11.9709600000
H	17.2047140000	7.3147360000	11.2522170000
C	16.8132200000	6.7492930000	13.2861790000
H	17.8445150000	6.8514490000	13.5994180000
C	15.8436780000	6.3574370000	14.1960130000
H	16.1134230000	6.1508130000	15.2240990000

C	14.5224650000	6.2215940000	13.7959470000
H	13.7828560000	5.9065890000	14.5209640000
C	8.5867980000	10.7197440000	11.6226090000
C	8.5765590000	11.0530360000	12.9810870000
H	9.4595870000	10.8810250000	13.5869750000
C	7.4574470000	11.6353710000	13.5566600000
H	7.4742260000	11.9075060000	14.6043980000
C	6.3190920000	11.8551910000	12.7942680000
H	5.4439380000	12.3080800000	13.2427610000
C	6.2977790000	11.4678280000	11.4624550000
H	5.4024840000	11.6082180000	10.8697560000
C	7.4242690000	10.9082780000	10.8765440000
H	7.3933510000	10.6223410000	9.8328340000
C	11.0797520000	11.7582160000	10.7335540000
C	12.0946580000	11.8287810000	9.7747160000
H	12.2945010000	10.9759210000	9.1353000000
C	12.8526390000	12.9772410000	9.6267950000
H	13.6302840000	13.0113220000	8.8739340000
C	12.6161270000	14.0788770000	10.4377840000
H	13.2080870000	14.9779170000	10.3217130000
C	11.6154380000	14.0201400000	11.3935310000
H	11.4178380000	14.8745540000	12.0287660000
C	10.8516540000	12.8702030000	11.5408050000
H	10.0688630000	12.8562200000	12.2874130000
H	9.8336800000	7.6553150000	12.1947130000
N	10.8074610000	7.5402160000	9.7574890000
H	9.9659040000	7.0476810000	10.0824790000
C	7.6115590000	7.0122780000	11.9757830000
C	8.8006970000	6.0698800000	11.8597460000
H	9.2564590000	5.7132710000	12.7890690000
O	9.0723590000	5.5644110000	10.7742200000
C	6.9003270000	7.1401460000	13.3332020000
C	7.3457500000	7.9400310000	15.6971940000
C	7.9694490000	7.3708590000	14.4505530000
O	7.4649060000	9.1299800000	15.9548390000
C	5.9783400000	8.3453150000	13.2555910000
C	6.5134620000	7.0645100000	16.5518140000
C	6.7692970000	5.7010230000	16.7005840000
C	5.9406330000	4.9172970000	17.4858990000
C	4.8391850000	5.4823510000	18.1127170000
C	4.5780990000	6.8398760000	17.9730280000
C	5.4169890000	7.6284850000	17.2077110000

C	6.1353010000	5.8255120000	13.5339110000
C	6.8101770000	4.6112460000	13.6866600000
C	6.1246600000	3.4232760000	13.8787360000
C	4.7392380000	3.4177470000	13.9319870000
C	4.0536860000	4.6126930000	13.7817660000
C	4.7420060000	5.7997550000	13.5743880000
H	8.5006070000	6.4445840000	14.6762280000
H	5.3743430000	8.4519490000	14.1564630000
H	6.5768260000	9.2448230000	13.1442200000
H	7.6311520000	5.2544590000	16.2213760000
H	6.1532760000	3.8624350000	17.6058300000
H	4.1833310000	4.8646860000	18.7133290000
H	3.7169630000	7.2797380000	18.4595480000
H	5.2219800000	8.6870360000	17.0845300000
H	7.8920070000	4.5792870000	13.7062260000
H	6.6820540000	2.5022660000	13.9998400000
H	4.2000930000	2.4926470000	14.0916880000
H	2.9717400000	4.6279290000	13.8253880000
H	4.1757640000	6.7134810000	13.4596760000
H	5.3053040000	8.2833800000	12.4036900000
H	8.6995020000	8.0979120000	14.0932460000
O	7.2060040000	7.5608620000	10.9735270000

H_2

H	-5.8397390000	-0.7819130000	0.0000000000
H	-5.0989760000	-0.8555830000	0.0000000000

NH_3

N	0.9062540000	0.0481900000	0.0830050000
H	0.6018410000	0.8623090000	-0.4386160000
H	0.6018470000	-0.7488360000	-0.4643920000
H	1.9175280000	0.0492970000	0.0136110000

H_2O

O	-8.2269170000	-1.6385340000	6.2519990000
H	-7.2656140000	-1.5900720000	6.2468560000

H	-8.5014060000	-0.7211080000	6.1546480000

PE

C	-0.8024300000	0.6971140000	0.8799150000
C	-1.3573300000	-0.0056070000	-0.1804690000
C	-0.5350980000	-0.7491770000	-1.0152120000
C	0.8322730000	-0.7896680000	-0.7872540000
C	1.3977700000	-0.0874410000	0.2726720000
C	0.5658890000	0.6584030000	1.1029380000
C	2.8924550000	-0.1007600000	0.4783210000
C	3.5614360000	1.0668880000	-0.2099200000
O	3.2497440000	-0.0150300000	1.8581190000
H	-1.4382960000	1.2787600000	1.5362080000
H	-2.4259600000	0.0225940000	-0.3529810000
H	-0.9606790000	-1.3043970000	-1.8421060000
H	1.4736270000	-1.3766740000	-1.4362230000
H	0.9975760000	1.2066790000	1.9322120000
H	3.2873360000	-1.0346350000	0.0582500000
H	3.1887800000	2.0103190000	0.1919280000
H	3.3602870000	1.0500610000	-1.2798880000
H	4.6408030000	1.0324010000	-0.0637910000
H	2.8523950000	-0.7648450000	2.3165410000

Intermediate I

C	-0.7260420000	1.1799060000	0.1212530000
C	-1.3486670000	-0.0593910000	0.1523600000
C	-0.5946760000	-1.2204780000	0.0325630000
C	0.7762780000	-1.1398670000	-0.1183710000
C	1.4152040000	0.1016550000	-0.1515730000
C	0.6481340000	1.2615790000	-0.0294620000
C	2.8875720000	0.1496840000	-0.3163180000
C	3.5650900000	1.4807310000	-0.3421270000
O	3.5340650000	-0.8838830000	-0.4293270000
H	-1.3129610000	2.0846250000	0.2142690000
H	-2.4232490000	-0.1219950000	0.2704630000
H	-1.0809620000	-2.1873670000	0.0575190000
H	1.3751510000	-2.0370770000	-0.2124530000
H	1.1240900000	2.2332350000	-0.0528600000

H	3.1774010000	2.0964650000	-1.1544740000
H	4.6358760000	1.3518450000	-0.4698850000
H	3.3765510000	2.0283420000	0.5820140000

EG

C	1.0334570000	0.0146230000	-0.1099430000
C	0.4786110000	0.2757650000	-1.4917320000
O	2.4521690000	0.0413420000	-0.0982130000
O	-0.9398780000	0.3079720000	-1.4957090000
H	0.6516070000	-0.9407480000	0.2712310000
H	0.7016260000	0.7935480000	0.5788910000
H	0.8599220000	-0.4761350000	-2.1938820000
H	0.8134730000	1.2510520000	-1.8495730000
H	2.7664740000	-0.7012120000	-0.6269180000
H	-1.2565490000	-0.5792460000	-1.2910870000

Intermediate III

C	1.0276870000	0.1169920000	-0.0695050000
C	0.4538360000	1.2586220000	-0.8517280000
O	2.4359250000	0.1162930000	-0.0364050000
O	-0.7250940000	1.3926360000	-1.0747220000
H	0.6105030000	-0.8207360000	-0.4534860000
H	0.6736460000	0.2140250000	0.9608690000
H	1.2029210000	1.9965080000	-1.2161250000
H	2.7574890000	-0.1418500000	-0.9076510000

Intermediate IX

C	0.2278530000	-0.8917870000	0.3495440000
C	0.2222500000	0.5175010000	0.8769240000
O	0.0641830000	-1.8234710000	1.1279740000
C	-1.1942040000	1.1351250000	0.9603150000
C	-2.0601070000	0.3976290000	1.9656840000
O	-0.9354770000	2.4556540000	1.4524190000
C	0.3689430000	-1.1467370000	-1.1032770000
C	0.7494750000	-0.1590790000	-2.0147120000
C	0.8021570000	-0.4383930000	-3.3700150000

C	0.4745510000	-1.7040710000	-3.8334000000
C	0.1031610000	-2.6977570000	-2.9354950000
C	0.0539930000	-2.4216440000	-1.5832670000
C	-1.8139360000	1.1959640000	-0.4289080000
C	-1.5355260000	2.2711780000	-1.2693800000
C	-2.0049870000	2.2955160000	-2.5739060000
C	-2.7593950000	1.2395640000	-3.0645680000
C	-3.0484580000	0.1660710000	-2.2355640000
C	-2.5831670000	0.1465980000	-0.9286940000
H	0.8358340000	1.1833240000	0.2720150000
H	0.6324390000	0.4985220000	1.8867900000
H	-2.1995240000	-0.6515620000	1.7131380000
H	-3.0446840000	0.8620750000	2.0293170000
H	-1.5936230000	0.4431450000	2.9489190000
H	-1.7849040000	2.9038700000	1.5535840000
H	1.0097660000	0.8328460000	-1.6713320000
H	1.0980700000	0.3358570000	-4.0664280000
H	0.5090280000	-1.9179430000	-4.8943060000
H	-0.1539520000	-3.6856460000	-3.2958120000
H	-0.2483080000	-3.1837130000	-0.8761030000
H	-0.9338520000	3.0910640000	-0.8974020000
H	-1.7751660000	3.1408600000	-3.2109310000
H	-3.1217020000	1.2544400000	-4.0848380000
H	-3.6384630000	-0.6635030000	-2.6057270000
H	-2.8077530000	-0.7090040000	-0.3033370000

Intermediate II

C	-0.8736050000	0.6700330000	1.0406890000
C	-1.4231110000	-0.5044960000	0.5461570000
C	-0.6275610000	-1.4079720000	-0.1477820000
C	0.7148820000	-1.1394610000	-0.3410200000
C	1.2721480000	0.0486090000	0.1342170000
C	0.4651270000	0.9510300000	0.8278820000
C	2.7215490000	0.2905290000	-0.0481920000
C	3.2798150000	1.6444550000	-0.0374610000
O	3.4926250000	-0.6669430000	-0.1474840000
C	2.8207620000	2.7569010000	-0.6475410000
C	3.5919660000	4.0119320000	-0.5617020000
C	1.5673380000	2.8056480000	-1.4604260000
C	4.9726610000	4.0238420000	-0.3305520000

C	5.6696100000	5.2143830000	-0.2304930000
C	5.0073680000	6.4279170000	-0.3568800000
C	3.6418510000	6.4362220000	-0.5969590000
C	2.9452420000	5.2445710000	-0.7097280000
H	-1.4902110000	1.3674570000	1.5931610000
H	-2.4727640000	-0.7186470000	0.7039130000
H	-1.0578370000	-2.3227430000	-0.5350760000
H	1.3475950000	-1.8388900000	-0.8735640000
H	0.8982620000	1.8642480000	1.2175800000
H	4.2428090000	1.6864860000	0.4603960000
H	1.1977580000	1.8198120000	-1.7237710000
H	1.7392830000	3.3588100000	-2.3842170000
H	0.7676790000	3.3282220000	-0.9314120000
H	5.5140180000	3.0893590000	-0.2571550000
H	6.7392340000	5.1947480000	-0.0630680000
H	5.5544150000	7.3589500000	-0.2792840000
H	3.1138400000	7.3758320000	-0.7009900000
H	1.8793260000	5.2738080000	-0.8943490000

Intermediate IV

C	-1.6986380000	-0.1334020000	0.6117130000
C	-1.6360390000	-0.7611250000	-0.6260330000
C	-0.4150950000	-0.9139270000	-1.2680990000
C	0.7406000000	-0.4198820000	-0.6872400000
C	0.6873270000	0.2159370000	0.5537520000
C	-0.5426330000	0.3412050000	1.2033440000
C	1.8820830000	0.8056330000	1.1982050000
C	2.9750810000	1.3805720000	0.3408390000
O	1.9417000000	0.9019640000	2.4174210000
C	2.5688750000	2.7551000000	-0.2703010000
C	1.5945910000	2.5682320000	-1.4303490000
C	2.0095000000	3.6471620000	0.8331630000
C	2.0006100000	1.8862540000	-2.5813340000
C	1.1309340000	1.6825030000	-3.6398120000
C	-0.1759290000	2.1439970000	-3.5705680000
C	-0.5936110000	2.8241950000	-2.4381680000
C	0.2843140000	3.0415070000	-1.3844840000
C	3.8902630000	3.4242730000	-0.7738030000
O	4.7747150000	2.5269590000	-1.3930150000
C	3.5926380000	4.5708080000	-1.6932110000

O	4.1038600000	4.6548050000	-2.7867170000
H	-2.6514700000	-0.0112590000	1.1107560000
H	-2.5408460000	-1.1332880000	-1.0900340000
H	-0.3638980000	-1.4149710000	-2.2265280000
H	1.6893140000	-0.5427380000	-1.1931080000
H	-0.5776240000	0.8413810000	2.1637230000
H	3.2515450000	0.6974900000	-0.4617110000
H	3.8489600000	1.5263460000	0.9764090000
H	1.0818300000	3.2527280000	1.2464740000
H	1.8021040000	4.6566030000	0.4800360000
H	2.7254230000	3.7194960000	1.6518320000
H	3.0066730000	1.4907900000	-2.6399190000
H	1.4734530000	1.1492340000	-4.5181340000
H	-0.8612430000	1.9741630000	-4.3914620000
H	-1.6098890000	3.1924100000	-2.3686150000
H	-0.0720110000	3.5775560000	-0.5149680000
H	4.3756470000	3.8558810000	0.1155250000
H	4.9681640000	2.8977750000	-2.2708140000
H	2.8904630000	5.3393510000	-1.3232870000

Intermediate VII

C	-1.7062230000	-0.2713850000	0.5106690000
C	-1.4964680000	-0.9362040000	-0.6910140000
C	-0.2233310000	-0.9939350000	-1.2406180000
C	0.8365450000	-0.3709210000	-0.6040290000
C	0.6353940000	0.3045810000	0.6010110000
C	-0.6458820000	0.3355460000	1.1569860000
C	1.7162360000	1.0358150000	1.2988840000
C	2.8660340000	1.5991770000	0.5111660000
O	1.6416670000	1.2468340000	2.5031540000
C	2.4948190000	2.9296450000	-0.2023840000
C	1.5526140000	2.6626330000	-1.3801630000
C	1.9189400000	3.9302510000	0.7976520000
C	2.0395990000	2.0222240000	-2.5204350000
C	1.1981840000	1.7028540000	-3.5735590000
C	-0.1528090000	2.0102340000	-3.5030290000
C	-0.6470930000	2.6522510000	-2.3780900000
C	0.1998150000	2.9874930000	-1.3309140000
C	3.7773280000	3.4977460000	-0.8034830000
O	4.7842240000	2.8340260000	-0.9521160000

C	3.7822150000	4.9300480000	-1.2604030000
O	4.9341630000	5.2242130000	-2.0075870000
H	-2.6996790000	-0.2235640000	0.9379550000
H	-2.3265070000	-1.4112240000	-1.1987920000
H	-0.0562540000	-1.5218710000	-2.1710230000
H	1.8252930000	-0.4220840000	-1.0406250000
H	-0.7978390000	0.8669940000	2.0885190000
H	3.2313600000	0.8906230000	-0.2316460000
H	3.6784560000	1.8005610000	1.2090340000
H	1.0307830000	3.5340090000	1.2874800000
H	1.6342230000	4.8647540000	0.3168740000
H	2.6481230000	4.1513950000	1.5766420000
H	3.0880890000	1.7506290000	-2.5793690000
H	1.5991320000	1.2062630000	-4.4483020000
H	-0.8148270000	1.7531680000	-4.3202480000
H	-1.6998700000	2.8976690000	-2.3107050000
H	-0.2116330000	3.4855590000	-0.4628730000
H	3.7226590000	5.5663440000	-0.3671750000
H	2.8816800000	5.1379110000	-1.8477240000
H	5.5178790000	4.4544920000	-1.9008740000

Intermediate VIII

C	-0.7641130000	1.3621650000	-0.5662940000
C	-1.5291990000	0.2127560000	-0.4291390000
C	-0.9090650000	-0.9925000000	-0.1368450000
C	0.4679620000	-1.0484770000	0.0201050000
C	1.2461330000	0.0979530000	-0.1150760000
C	0.6115520000	1.3040590000	-0.4104530000
C	2.7484020000	0.0452030000	-0.0090840000
C	3.3223910000	-1.1472930000	0.7557680000
O	3.3530480000	-0.0263670000	-1.3167030000
C	4.7779260000	-0.7742120000	1.1318500000
C	5.0845400000	-1.1353950000	2.5810050000
C	5.8136940000	-1.4313300000	0.2166910000
C	5.4874210000	-0.2070810000	3.5365480000
C	5.7430100000	-0.6008210000	4.8446130000
C	5.6017720000	-1.9263880000	5.2189460000
C	5.1976320000	-2.8608180000	4.2746580000
C	4.9420240000	-2.4682200000	2.9719650000
C	4.8170690000	0.7353850000	0.9004750000

O	5.8165550000	1.4151070000	0.8953000000
C	3.4002010000	1.2491360000	0.6752350000
O	2.8267170000	1.6151390000	1.9211360000
H	-1.2402360000	2.3082280000	-0.7918760000
H	-2.6044450000	0.2571970000	-0.5484850000
H	-1.4985750000	-1.8944220000	-0.0286970000
H	0.9388390000	-1.9970140000	0.2474120000
H	1.1994460000	2.2083710000	-0.5204180000
H	3.2697200000	-2.0565080000	0.1578430000
H	2.7278620000	-1.3190430000	1.6537970000
H	3.0894930000	0.7589650000	-1.8154760000
H	5.7940960000	-2.5132650000	0.3420970000
H	6.8160050000	-1.0783820000	0.4586190000
H	5.6139280000	-1.2069700000	-0.8301340000
H	5.6044230000	0.8366080000	3.2732190000
H	6.0531570000	0.1394350000	5.5717040000
H	5.8014290000	-2.2312570000	6.2384270000
H	5.0794620000	-3.9005280000	4.5540410000
H	4.6214870000	-3.2062550000	2.2451010000
H	3.3925420000	2.1446630000	0.0543860000
H	2.7456920000	0.8345750000	2.4867390000

Product 1

O	0.3562080000	5.7757170000	10.2578770000
O	1.5440670000	3.3961280000	11.1120380000
H	0.7343910000	3.8446190000	11.4129890000
C	1.3960820000	5.3748520000	9.7460920000
C	2.1983810000	6.0650530000	8.6530700000
C	3.3542900000	5.0690140000	8.4118900000
H	3.3450970000	4.6809200000	7.3907170000
H	4.3239790000	5.5576690000	8.5362320000
C	3.1893280000	3.9524020000	9.4008770000
C	2.0631240000	4.1573790000	10.1319770000
C	1.3110410000	6.2445790000	7.4331120000
C	0.7802730000	5.1119410000	6.8112600000
H	1.0247550000	4.1277590000	7.1986860000
C	-0.0531250000	5.2233570000	5.7122380000
H	-0.4529320000	4.3303290000	5.2479510000
C	-0.3764640000	6.4765730000	5.2078480000
H	-1.0270380000	6.5673190000	4.3471650000

C	0.1396300000	7.6078470000	5.8171760000
H	-0.1052360000	8.5907750000	5.4337790000
C	0.9727760000	7.4936480000	6.9237770000
H	1.3618560000	8.3928120000	7.3830890000
C	4.1352240000	2.8605330000	9.4892410000
C	3.9987220000	1.8276670000	10.4312860000
H	3.1656300000	1.8359820000	11.1189680000
C	4.9202700000	0.7988110000	10.4907040000
H	4.7963140000	0.0130860000	11.2253840000
C	5.9993620000	0.7682990000	9.6172850000
H	6.7181330000	-0.0398280000	9.6674120000
C	6.1501780000	1.7801460000	8.6794710000
H	6.9881810000	1.7646190000	7.9940260000
C	5.2323040000	2.8124880000	8.6155740000
H	5.3644410000	3.5933150000	7.8777600000
C	2.7181300000	7.3791550000	9.2266430000
H	3.3209130000	7.9156350000	8.4944780000
H	3.3438300000	7.1855140000	10.0979480000
H	1.8994880000	8.0263030000	9.5408650000

Intermediate V

C	0.6310240000	-0.6281480000	0.6657540000
C	0.1949990000	0.7978480000	0.8718400000
O	0.9758180000	-1.3120300000	1.6197780000
C	-1.3568300000	0.9303140000	0.8457970000
C	-1.9855060000	-0.1370030000	1.7417840000
C	-1.7841190000	2.2762760000	1.4385940000
C	-0.8366340000	3.4607470000	1.6070620000
O	-2.9236610000	2.4959140000	1.7838900000
O	0.0189640000	3.7646580000	0.8188500000
C	0.5434670000	-1.2175110000	-0.6886790000
C	0.7032760000	-0.4604620000	-1.8502790000
C	0.5596580000	-1.0523720000	-3.0935070000
C	0.2344160000	-2.3983760000	-3.1892610000
C	0.0779530000	-3.1610840000	-2.0388650000
C	0.2440830000	-2.5772910000	-0.7965710000
C	-1.8888160000	0.8899090000	-0.5874930000
C	-1.5499360000	1.9128510000	-1.4746410000
C	-1.9657820000	1.8776710000	-2.7951510000
C	-2.7264230000	0.8133180000	-3.2578220000

C	-3.0785260000	-0.2023340000	-2.3829440000
C	-2.6739440000	-0.1595060000	-1.0557420000
H	0.6322220000	1.4518150000	0.1186090000
H	0.5631830000	1.1135390000	1.8491230000
H	-1.7685840000	-1.1405530000	1.3788970000
H	-3.0660170000	-0.0211230000	1.7884800000
H	-1.5888620000	-0.0604970000	2.7539060000
H	-1.0956000000	4.0869000000	2.4846120000
H	0.9547180000	0.5899540000	-1.7834500000
H	0.6953230000	-0.4607130000	-3.9900390000
H	0.1045250000	-2.8549230000	-4.1624900000
H	-0.1782590000	-4.2101380000	-2.1138870000
H	0.1152850000	-3.1573420000	0.1093230000
H	-0.9315170000	2.7364160000	-1.1338940000
H	-1.6887170000	2.6816350000	-3.4655800000
H	-3.0449110000	0.7780620000	-4.2919890000
H	-3.6729800000	-1.0381700000	-2.7308820000
H	-2.9604280000	-0.9668210000	-0.3952700000

Intermediate VI

C	0.4580290000	-1.0451080000	-0.0913330000
C	0.0530350000	-0.3081130000	1.1554200000
O	0.7146820000	-2.2417560000	-0.0525980000
C	-1.4872930000	-0.0604350000	1.2140830000
C	-2.1817730000	-1.3458830000	0.7599140000
C	-1.9106530000	0.1470910000	2.7026550000
C	-1.3789040000	1.3449590000	3.4765450000
O	-3.3294070000	0.1913630000	2.8197470000
O	0.0283350000	1.4856600000	3.5144720000
C	0.4572000000	-0.3281290000	-1.3878300000
C	0.7758020000	1.0253430000	-1.4941760000
C	0.7301440000	1.6591910000	-2.7247420000
C	0.3468720000	0.9532880000	-3.8561170000
C	0.0318190000	-0.3965670000	-3.7611800000
C	0.0992910000	-1.0362400000	-2.5372080000
C	-1.8888400000	1.1428030000	0.3695620000
C	-1.2515520000	2.3763850000	0.5391060000
C	-1.5976700000	3.4800260000	-0.2223140000
C	-2.5923680000	3.3812550000	-1.1849230000
C	-3.2392940000	2.1699960000	-1.3650130000
C	-2.8960170000	1.0679350000	-0.5919980000

H	0.5946880000	0.6316720000	1.2373770000
H	0.3372390000	-0.9303770000	2.0051990000
H	-1.9980290000	-1.5613040000	-0.2930850000
H	-3.2581490000	-1.2962560000	0.9035750000
H	-1.8077360000	-2.1918230000	1.3380800000
H	-1.6108650000	-0.7656260000	3.2290620000
H	-1.7910760000	1.2725300000	4.4893890000
H	-1.7734150000	2.2687390000	3.0474640000
H	-3.6398020000	1.0007800000	2.3907220000
H	0.3955060000	0.7090360000	3.9527770000
H	1.0819520000	1.5788220000	-0.6162660000
H	0.9893370000	2.7077920000	-2.7998650000
H	0.2952740000	1.4545850000	-4.8144900000
H	-0.2693840000	-0.9468330000	-4.6434550000
H	-0.1490930000	-2.0870460000	-2.4477090000
H	-0.4455160000	2.4710860000	1.2568190000
H	-1.0786330000	4.4184570000	-0.0700750000
H	-2.8578720000	4.2401120000	-1.7884850000
H	-4.0182220000	2.0748960000	-2.1115700000
H	-3.4191810000	0.1358460000	-0.7585130000

9. References

(1) Espinosa-Jalapa, N. A.; Kumar, A.; Leitus, G.; Diskin-Posner, Y.; Milstein, D. Synthesis of Cyclic Imides by Acceptorless Dehydrogenative Coupling of Diols and Amines Catalyzed by a Manganese Pincer Complex. *J. Am. Chem. Soc.* **2017,** *139*, 11722-11725.

(2) Nerush, A.; Vogt, M.; Gellrich, U.; Leitus, G.; Ben-David, Y.; Milstein, D. Template Catalysis by Metal-Ligand Cooperation. C-C Bond Formation Via Conjugate Addition of Non-Activated Nitriles under Mild, Base-Free Conditions Catalyzed by a Manganese Pincer Complex. *J. Am. Chem. Soc.* **2016,** *138*, 6985-6997.

(3) Das, U. K.; Ben-David, Y.; Leitus, G.; Diskin-Posner, Y.; Milstein, D. Dehydrogenative Cross-Coupling of Primary Alcohols to Form Cross-Esters Catalyzed by a Manganese Pincer Complex. *ACS Catal.* **2018,** *9*, 479-484.

(4) Lu, L.; Luo, J.; Montag, M.; Diskin-Posner, Y.; Milstein, D. Polyoxymethylene Upcycling into Methanol and Methyl Groups Catalyzed by a Manganese Pincer Complex. *J. Am. Chem. Soc.* **2024,** *146*, 22017-22026.

(5) Daw, P.; Kumar, A.; Espinosa-Jalapa, N. A.; Diskin-Posner, Y.; Ben-David, Y.; Milstein, D. Synthesis of Pyrazines and Quinoxalines Via Acceptorless Dehydrogenative Coupling Routes Catalyzed by Manganese Pincer Complexes. *ACS Catal.* **2018,** *8*, 7734-7741.

(6) Meng, X.; Chen, D.; Cao, X.; Luo, J.; Wang, F.; Huang, S. Synthesis of Polysubstituted Cyclic 1,2-Diketones Enabled by Iterative Sulfoxide-Mediated Arylation. *Chem. Commun.* **2019,** *55*, 12495-12498.

(7) Sheldrick, G. M. Shelxt - Integrated Space-Group and Crystal-Structure Determination. *Acta Cryst.* **2015,** *A71*, 3-8.

(8) Sheldrick, G. M. A Short History of Shelx. *Acta Cryst.* **2008,** *A64*, 112-122.

(9) Dolomanov, O. V.; Bourhis, L. J.; Gildea, R. J.; Howard, J. A. K.; Puschmann, H. Olex2: A Complete Structure Solution, Refinement and Analysis Program. *J. Appl. Cryst.* **2009,** *42*, 339-341.

(10) M. J. Frisch; G. W. Trucks; H. B. Schlegel; G. E. Scuseria; M. A. Robb; J. R. Cheeseman; G. Scalmani; V. Barone; G. A. Petersson; H. Nakatsuji; X. Li; M. Caricato; A. V. Marenich; J. Bloino; B. G. Janesko; R. Gomperts; B. Mennucci; H. P. Hratchian; J. V. Ortiz; A. F. Izmaylov; J. L. Sonnenberg; D. Williams-Young; F. Ding; F. Lipparini; F. Egidi; J. Goings; B. Peng; A. Petrone; T. Henderson; D. Ranasinghe; V. G. Zakrzewski; J. Gao; N. Rega; G. Zheng; W. Liang; M. Hada; M. Ehara; K. Toyota; R. Fukuda; J. Hasegawa; M. Ishida; T. Nakajima; Y. Honda; O. Kitao; H. Nakai; T. Vreven; K. Throssell; J. A. Montgomery; Jr.; J. E. Peralta; F. Ogliaro; M. J. Bearpark; J. J. Heyd; E. N. Brothers; K. N. Kudin; V. N. Staroverov; T. A. Keith; R. Kobayashi; J. Normand; K. Raghavachari; A. P. Rendell; J. C. Burant; S. S. Iyengar; J. Tomasi; M. Cossi; J. M. Millam; M. Klene; C. Adamo; R. Cammi; J. W. Ochterski; R. L. Martin; K. Morokuma; O. Farkas; J. B. Foresman; Fox, D. J., Gaussian 16, Revision C.01. Gaussian, Inc., Wallingford CT, 2016.

(11) Zhao, Y.; Truhlar, D. G. A New Local Density Functional for Main-Group Thermochemistry, Transition Metal Bonding, Thermochemical Kinetics, and Noncovalent Interactions. *J. Chem. Phys.* **2006,** *125*, 194101.

(12) Weigend, F.; Ahlrichs, R. Balanced Basis Sets of Split Valence, Triple Zeta Valence and Quadruple Zeta Valence Quality for H to Rn: Design and Assessment of Accuracy. *Phys. Chem. Chem. Phys.* **2005,** *7*, 3297-3305.

(13) Weigend, F. Accurate Coulomb-Fitting Basis Sets for H to Rn. *Phys. Chem. Chem. Phys.* **2006,** *8*, 1057-1065.
(14) Grimme, S.; Antony, J.; Ehrlich, S.; Krieg, H. A Consistent and Accurate Ab Initio Parametrization of Density Functional Dispersion Correction (Dft-D) for the 94 Elements H-Pu. *J. Chem. Phys.* **2010,** *132*, 154104.
(15) Neese, F. Software Update: The Orca Program System—Version 5.0. *WIREs Comput. Mol. Sci.* **2022,** *12*, e1606.
(16) Mardirossian, N.; Head-Gordon, M. Omegab97x-V: A 10-Parameter, Range-Separated Hybrid, Generalized Gradient Approximation Density Functional with Nonlocal Correlation, Designed by a Survival-of-the-Fittest Strategy. *Phys. Chem. Chem. Phys.* **2014,** *16*, 9904-9924.
(17) Vydrov, O. A.; Van Voorhis, T. Nonlocal Van Der Waals Density Functional: The Simpler the Better. *J. Chem. Phys.* **2010,** *133*, 244103.
(18) Hujo, W.; Grimme, S. Performance of the Van Der Waals Density Functional Vv10 and (Hybrid)Gga Variants for Thermochemistry and Noncovalent Interactions. *J. Chem. Theory Comput.* **2011,** *7*, 3866-3871.
(19) Hellweg, A.; Hättig, C.; Höfener, S.; Klopper, W. Optimized Accurate Auxiliary Basis Sets for Ri-Mp2 and Ri-Cc2 Calculations for the Atoms Rb to Rn. *Theor. Chem. Acc.* **2007,** *117*, 587-597.
(20) Iron, M. A.; Janes, T. Evaluating Transition Metal Barrier Heights with the Latest Density Functional Theory Exchange-Correlation Functionals: The Mobh35 Benchmark Database. *J. Phys. Chem. A* **2019,** *123*, 3761-3781.
(21) Marenich, A. V.; Cramer, C. J.; Truhlar, D. G. Universal Solvation Model Based on Solute Electron Density and on a Continuum Model of the Solvent Defined by the Bulk Dielectric Constant and Atomic Surface Tensions. *J. Phys. Chem. B* **2009,** *113*, 6378-6396.
(22) Cramer, C. J., *Essentials of Computational Chemistry: Theories and Models*. 2nd ed.; John Wiley & Sons Ltd: West Sussex, England, 2004.
(23) Sparta, M.; Riplinger, C.; Neese, F. Mechanism of Olefin Asymmetric Hydrogenation Catalyzed by Iridium Phosphino-Oxazoline: A Pair Natural Orbital Coupled Cluster Study. *J. Chem. Theory Comput.* **2014,** *10*, 1099-1108.
(24) Hopmann, K. H. How Accurate Is Dft for Iridium-Mediated Chemistry? *Organometallics* **2016,** *35*, 3795-3807.
(25) Gusev, D. G. Revised Mechanisms of the Catalytic Alcohol Dehydrogenation and Ester Reduction with the Milstein Pnn Complex of Ruthenium. *Organometallics* **2020,** *39*, 258-270.
